# Cytomegalovirus Generates Assembly Compartment in the Early Phase of Infection by Perturbation of Host-Cell Factors Recruitment at the Early Endosome/Endosomal Recycling Compartment/Trans-Golgi Interface

**DOI:** 10.3389/fcell.2020.563607

**Published:** 2020-09-11

**Authors:** Pero Lučin, Natalia Jug Vučko, Ljerka Karleuša, Hana Mahmutefendić Lučin, Gordana Blagojević Zagorac, Berislav Lisnić, Valentino Pavišić, Marina Marcelić, Kristina Grabušić, Ilija Brizić, Silvija Lukanović Jurić

**Affiliations:** ^1^Department of Physiology and Immunology, Faculty of Medicine, University of Rijeka, Rijeka, Croatia; ^2^University North, University Center Varaždin, Varaždin, Croatia; ^3^Department of Histology and Embryology, Faculty of Medicine, University of Rijeka, Rijeka, Croatia

**Keywords:** cytomegalovirus, virion assembly compartment, endosomal recycling compartment, Rab proteins, Rab cascades

## Abstract

Beta-herpesviruses develop a unique structure within the infected cell known as an assembly compartment (AC). This structure, as large as the nucleus, is composed of host-cell-derived membranous elements. The biogenesis of the AC and its contribution to the final stages of beta-herpesvirus assembly are still unclear. In this study, we performed a spatial and temporal analysis of the AC in cells infected with murine CMV (MCMV), a member of the beta-herpesvirus family, using a panel of markers that characterize membranous organelle system. Out of 64 markers that were analyzed, 52 were cytosolic proteins that are recruited to membranes as components of membrane-shaping regulatory cascades. The analysis demonstrates that MCMV infection extensively reorganizes interface between early endosomes (EE), endosomal recycling compartment (ERC), and the trans-Golgi network (TGN), resulting in expansion of various EE-ERC-TGN intermediates that fill the broad area of the inner AC. These intermediates are displayed as over-recruitment of host-cell factors that control membrane flow at the EE-ERC-TGN interface. Most of the reorganization is accomplished in the early (E) phase of infection, indicating that the AC biogenesis is controlled by MCMV early genes. Although it is known that CMV infection affects the expression of a large number of host-cell factors that control membranous system, analysis of the host-cell transcriptome and protein expression in the E phase of infection demonstrated no sufficiently significant alteration in expression levels of analyzed markers. Thus, our study demonstrates that MCMV-encoded early phase function targets recruitment cascades of host cell-factors that control membranous flow at the EE-ERC-TGN interface in order to initiate the development of the AC.

## Introduction

Beta-herpesviruses infect almost all the human population, cause asymptomatic infections, and are associated with a wide range of pathologic conditions (rev in. Britt and Prichard, 2018). Despite the extensive and long-lasting efforts, the development of effective vaccines and antiviral therapies against beta-herpesviruses remains of outstanding importance (Britt and Prichard, 2018). Among several, potential viral and cellular targets of antiviral research are those that control critical points in the beta-herpesvirus life cycle, the assembly and egress of infectious virions.

The assembly of beta-herpesviruses occurs as a complex set of events in the nucleus and cytoplasm of the infected cell (rev in Tandon and Mocarski, 2012; Close et al., 2018a). The cytoplasmic stage requires a transformation of the membranous system of the cell, the establishment of the virus-encoded tegument matrix, and the accumulation of envelope glycoproteins at appropriate membranous organelle. Once the preparatory events in the membranous system are completed, pre-assembled nucleocapsids migrate from the nucleus, move through the cytoplasm, and acquire most of the tegument components and membranous envelope harboring viral glycoproteins by budding into the membranous organelle(s). All these cytoplasmic events take place within a sizeable cytoplasmic structure known as the assembly compartment (AC).

The beta-herpesvirus AC has been mainly studied during human cytomegalovirus (HCMV) infection (rev in Tandon and Mocarski, 2012; Close et al., 2018a). The AC develops in CMV infected cells as a juxtanuclear cylindrical aggregate of membranous structures delimited at its periphery by the Golgi complex. Given that the AC area is large, approximately the size of the nucleus (Close et al., 2018a), it contains a large number of membranous intermediates that may serve as a site for final CMV envelopment. The AC is fully formed at late stages of infection after late viral genes encoding tegument, and envelope proteins are expressed. However, the reorganization of the host-cell organelles could be initiated earlier in the infection, delineated as preAC (PrAC; Taisne et al., 2019).

Electron microscopy (EM) studies of the HCMV AC demonstrated displacement of the Golgi stacks into a vacuolar ring that surrounds centrally accumulated aggregate of vesicular, vacuolar, and tubular membranous structures (Homman-Loudiyi et al., 2003; Buser et al., 2007; Maninger et al., 2011; Tandon and Mocarski, 2012; Schauflinger et al., 2013; Archer et al., 2017). Non-enveloped capsids were randomly spread throughout the AC area in contact with membranous intermediates and were not localized in specifically defined structures (Schauflinger et al., 2013), whereas. enveloped capsids are relatively rare. Accordingly, the release of newly enveloped infectious virions is a rare event that occurs with the frequency of 1 infectious virion per hour (Sampaio et al., 2005). Immunofluorescence studies (Homman-Loudiyi et al., 2003; Das et al., 2007; Krzyzaniak et al., 2009; Cepeda et al., 2010; Das and Pellett, 2011; Hook et al., 2014) demonstrated that HCMV AC represents an extensive reorganization of the Golgi, early endosomes (EE), and recycling endosomes (RE), leading to the proposal that the cellular secretory pathway machinery is used for virion envelopment and egress (Close et al., 2018b). Systems studies of the host-cell transcriptome and proteome demonstrated that HCMV infection alters a large number of host-cell proteins that may be associated with membranous organelle organization and thereby might drive the AC biogenesis (Weekes et al., 2014; Tirosh et al., 2015; Jean Beltran et al., 2016). However, spatial and temporal proteomic analysis (Jean Beltran et al., 2016) could not sufficiently distinguish membranous compartments or intermediates reorganized by CMV infection. Thus, the composition, organization, and the sequence of events that characterize the development of the AC remained mostly unclear, and consequently, the mechanisms underlying these extensive organelle remodeling events remained unknown (Jean Beltran et al., 2016; Close et al., 2018b).

The analysis of the membranous organization of the AC was mainly restricted to the classical steady-state organelles. However, many publications in the last decade demonstrate much more complexity in the organization and function of the membranous system of the cell (Goldenring, 2015; Villaseñor et al., 2016; Naslavsky and Caplan, 2018). Accordingly, almost all classical organelles can be further subdivided into various functional subsets or maturation intermediates. Furthermore, membranous organelles display the domain organization that is dynamically shaped by the cascade-like recruitment of regulatory and effector proteins (rev. in Cherfils and Zeghouf, 2013; Wandinger-Ness and Zerial, 2014; Pfeffer, 2017). The domain organization is mostly governed by the recruitment of a small GTPase from Rab and ARF family, followed by membrane lipid modification and mobilization of various effector proteins that define the phenotypic and functional identity of membrane domains. According to the Rab/ARF cascade hypothesis, membranous system dynamics is based on the flow of domains in which upstream Rab/ARF recruits a guanine exchange factor (GEF) to activate downstream Rab/ARF, which in turn recruit a GTPase activating (GAP) protein to inactivate upstream Rab/ARF. Thus, all the cascades form highly dynamic spatially and temporarily organized functional networks that can be reconfigured in the course of CVM infection. Accordingly, it is debatable whether CMV infection reorganizes spatial and temporal domain dynamics of classical steady-state organelles or modifies the order within regulatory cascades and thereby reorganizes the endomembrane system into a new organelle composition. To address these questions, it is essential to analyze further reorganized membranous organelles within the AC, especially endogenous recruitment of host-cell regulatory proteins that shape membrane domains, and to determine the sequence of the membrane system reorganization during the CMV replication cycle.

At the current stage of the AC understanding, it remains unclear what is the site of final CMV secondary envelopment, and how are newly formed CMV virions released from the infected cell. Despite several efforts to label CMV virion particles as a tool for visualization of the final stages of CMV maturation (Rupp et al., 2005; Sampaio et al., 2005), it seems that the use of stable fluorescent virions is a tedious task. Thus, to make further progress in the understanding of CMV maturation processes it is essential: (1) to establish precise composition and reveal the biogenesis of the AC; (2) to analyze CMV maturation processes using visualizable CMV capsids within the resolved composition of the AC; and (3) to establish a temporal and spatial functional network of regulatory and effector host-cell proteins that drive these processes using proteomic and interatomic data. In the present study, we attempted to address the first issue. We analyzed the composition and the biogenesis of the AC in cells infected with murine CMV (MCMV) at four stages of infection, including two stages of the PrAC and two stages of the AC.

Murine CMV is a member of the beta-herpesvirus family, with many similarities to HCMV and other members of the family (Brizić et al., 2018). MCMV replication cycle is much shorter in tissue culture conditions and, thus, more suitable for studies of the role of host-cell factors that require long-term perturbation of host-cell functions, such as host-cell gene silencing. Therefore, understanding the AC composition, its biogenesis, and contribution to the final MCMV assembly may be beneficial for a more in-depth understanding of these processes during HCMV infection as well as infections with other members of the beta-herpesvirus family.

## Materials and Methods

### Cell Lines, Viruses, and Infection Conditions

All the procedures in the cell culture laboratory, as well as the production of MCMV stocks and infection of cells with MCMV, have been performed according to standard procedures (Brizić et al., 2018). Balb 3T3 fibroblasts and murine dendritic cell line DC2.4 were obtained from American Type Culture Collection (ATCC), and primary murine embryonic fibroblasts (MEFs) were generated from 17 days embryos of BALB/c mice. Balb 3T3 cells were grown in DMEM, DC2.4 cells in RPMI, and MEFs in minimal essential medium (MEM), supplemented with 10% (v/v) of fetal bovine serum (FBS), 2 mM L-glutamine, 100 mg/ml of streptomycin and 100 U/ml penicillin (all reagents from Gibco/Invitrogen, Grand Island, NY, United States). The cells were grown in Petri dishes as adherent cell lines and used for infection when they were 90% confluent.

The recombinant virus Δm138-MCMV (ΔMC95.15), with the deletion of the fcr1 (m138) gene (Crnković-Mertens et al., 1998), was regularly used for infection. In some experiments, we used Δ9-MCMV, a recombinant MCMV with deletion of M23-M26 genes generated on the wild-type MCMV background, and MCMV wild-type strain Smith (ATCC VR-194; American Type Culture Collection [ATCC]).

Cells were infected at a multiplicity of infection (MOI) of 10 with an enhancement of infectivity by centrifugation (Brizić et al., 2018), and the efficiency of infection was monitored by the immunofluorescent detection of the intracellular immediate-early 1 (IE1) protein, as described previously (Karleuša et al., 2018).

### Antibodies and Reagents

Antibody reagents to host-cell factors and MCMV-encoded proteins were used either as monoclonal (MAbs) or polyclonal antibodies. MAbs to MHC class I (clone SF1-1.1.1 for H2-K^d^), murine transferrin (Tf) receptor (TfR) (clone R17 217.1.3), CD44 (clone IM7), and RAE1 (clone Rae1γ0.01) were used as hybridoma culture supernatant purified by affinity chromatography. Monoclonal antibodies to MCMV proteins are produced by the University of Rijeka Center for Proteomics. Other Ab reagents were purchased from different distributors. The sources of primary antibody reagents and references are presented in Supplementary Table S1. Alexa Fluor (AF)^488^- and AF^555^-conjugated secondary antibody reagents to mouse IgG_2__a_, mouse IgG_2__b_, mouse IgG_1_, rat IgG, rabbit IgG, and chicken IgG were from Molecular Probes (Leiden, Netherlands), and AF^647^-conjugated IgG_1_ and IgG_2__a_ were from Jacksons Laboratory (Bar Harbor, ME, United States).

### Immunofluorescence and Confocal Analysis

Cells grown on coverslips were fixed with 4% formaldehyde (20 min at r.t.) and permeabilized. All primary Ab reagents to membranous organelle markers were tested, in addition to optimal reagent concentration, for optimal detergent (Triton X-100, Tween 20, saponin, and methanol) concentration and permeabilization temperature. In most cases, we used permeabilization at 37^o^C for 20 min with 0.5–1% Tween 20. After permeabilization, cells were incubated with primary Ab reagents for 60 min. Unbound Ab reagents were washed with PBS, and cells were incubated for 60 min with an appropriate fluorochrome-conjugated secondary reagent. All secondary Ab reagents were tested for cross-reactivity against primary Abs and secondary Ab reagents used in combination with double and triple immunofluorescence staining. After the three washes in PBS, cells were embedded in Mowiol (Fluka Chemicals, Selzee, Germany)-DABCO (Sigma Chemical Co, Steinheim, Germany) in PBS containing 50% glycerol and analyzed by confocal microscopy.

Imaging was performed on an Olympus Fluoview FV300 confocal microscope (Olympus Optical Co., Tokyo, Japan) equipped with Ar 488, He/Ne 543, and He/Ne 633 lasers. Images were acquired using Fluoview software, version 4.3 FV 300 (Olympus Optical Co., Tokyo, Japan), PLAPO60xO objective and the appropriate filters, NA = 2, PMT 600–800, beam splitter at 570 nm, without Kalman filtering.

The z-series of 0.5 μm optical sections were acquired sequentially with medium scan speed (1,65s/scan), resulting in either 8–10 slices or 10–16 slices in uninfected and MCMV infected cell samples (cell rounding), respectively. All acquisition parameters were adjusted on uninfected cells, with the offset below 5%, and the z-series of infected cell samples were acquired under identical condition without any correction. The images are acquired using the 2x zoom at areas with at least three infected cells with representative staining patterns (images shown in Supplementary) and 4x and 8x zoom to display as much as possible distinct structures within individual infected cells.

The images (515 × 512 pixels) were exported as a TIFF and analyzed using ImageJ software and available plugins (Plot Profile and JACoP) without any image rendition and additional processing. Volume Viewer plugin was used for the reconstruction of 3D images of the entire z-series. Focus plane (usually fourth or fifth section) images were used for image presentation and colocalization presentation by plotting profiles along the line.

Colocalization events were quantitatively evaluated on 8x zoomed images using a global statistic approach that performs intensity correlation coefficient-based (ICCB) analyzes. We used ImageJ 1.47v software, utilizing the JACoP plugin^1^ (Bolte and Cordeliéres, 2006) to calculate Manders’ overlap coefficients (M1 and M2) within the entire z-stack for three dimensional (3D) analysis of colocalization. The background was partially eliminated during the image acquisition process by adjusting detector settings in order to detect the maximal fluorescence intensity in red and green channels. The best-fit lower threshold to eliminate most of the signal background (Costes automatic thresholding method) was determined using the threshold tool and confirmed by visual inspection. Measures were made on 6–10 cells per experiment on the entire z-series.

### Western-Blot

Cellular extracts for WB analysis were prepared in RIPA lysis buffer supplemented with protease and phosphatase inhibitors, separated by SDS-PAGE, and blotted onto a polyvinylidene difluoride (PVDF-P) WB membrane (Millipore) at 60 to 70 V for 1 h. Membranes were incubated with 1% blocking reagent (Roche Diagnostics GmbH, Mannheim, Germany) for 1 h, followed by 1-h to overnight incubation with primary Abs, three cycles of washing (TBS with 0.05% Tween 20 [TBS-T buffer]), and a 45-min incubation with peroxidase-conjugated secondary reagent diluted in TBS buffer containing 0.5% blocking reagent. After being washed three times with TBS-T buffer (pH 7.5), membranes were incubated for 1 min with ECL Prime substrate (GE Healthcare) and enveloped into plastic wrap. Signals were detected by Transilluminator Alliance 4.7 (Uvitec Ltd., Cambridge, United Kingdom).

### Total RNA Isolation, RNA Sequencing, and the Analysis of the RNA-Seq Data

A total of 2.6 × 10^7^ DC2.4 cells were grown in a 48-well plate in three triplicates in 10% RPMI without β-mercaptoethanol. One triplicate was mock-infected, while the cells in the two remaining triplicates were infected with wild-type MCMV at a MOI = 2. Total RNA isolation from mock-infected cells (3 h after mock infection), and wild-type MCMV infected cells (3 and 18 h after MCMV infection), was then performed using QIAzol Lysis reagent according to manufacturer instructions (QIAGEN, Germany). Following total RNA isolation, samples were treated with DNase I (New England Biolabs, United States) according to manufacturer recommendations in order to remove traces of contaminating chromosomal and/or mitochondrial DNA. Purified RNA was then transferred into RNA transport buffer (Omega Bioservices) and submitted to Omega Bioservices core sequencing facility (United States).

All RNA quality control procedures, polyA selection, cDNA library preparation, and sequencing have been performed at the Omega Bioservices core sequencing facility (United States). Quantification, sample purity assessment, and sample integrity assessment of isolated total RNA, performed on Nanodrop Spectrophotometer and Agilent Tapestation 2200, demonstrated that all RNA samples met the quality and quantity prerequisites required for downstream processing. Subsequently, the sequencing libraries were prepared using the TruSeq Stranded mRNA Prep Kit (Illumina, United States), and sequencing was performed on an Illumina HiSeq 2500 for a total of 51 sequencing cycles.

Quality control and the validation of RNA sequencing data are presented as Supplementary Figures S1–S6. Quality control of raw sequencing reads was performed using FastQC^2^. Additionally, the reads were screened for the most common biological contaminants of laboratory mice and cell cultures, as well as technical contaminants, such as vectors, adapters, and rRNA sequences. Following pre-mapping quality control, STAR aligner v.2.7.3a was used to map raw sequencing reads to a custom genome generated by concatenating the GENCODE nucleotide sequence of the mouse GRCm38.p6 primary genome assembly (release M23), and the wild-type MCMV genome sequence, strain Smith (PubMed accession no. NC_004065.1). Obtained coordinate sorted alignment files in.bam format were indexed using SAMTools v1.9. To identify potential errors, outliers, or other issues that could jeopardize differential expression analysis, visual inspection of mapping results, and comprehensive post-mapping quality controls were then performed in IGV v2.7.2 and QoRTs v1.3.6. Outputs from all supported tools were systematized using MultiQC v1.7. Summarization of reads mapping to exons of mouse genes was performed using featureCounts v2.0, and principal component analysis of the samples, gene-expression estimates, normalization of the expression data and differential expression analysis was performed using DESeq2 v1.26.0 in R programming environment v3.6.1 using RStudio v.1.2.5019 under Canonical Ubuntu v18.04 open-source operating system.

### Statistics

The significance of difference was tested using Student’s *t*-test (*p* < 0.05 was considered significant).

## Results

### Membranous Organelle Markers

**FIGURE 1:**
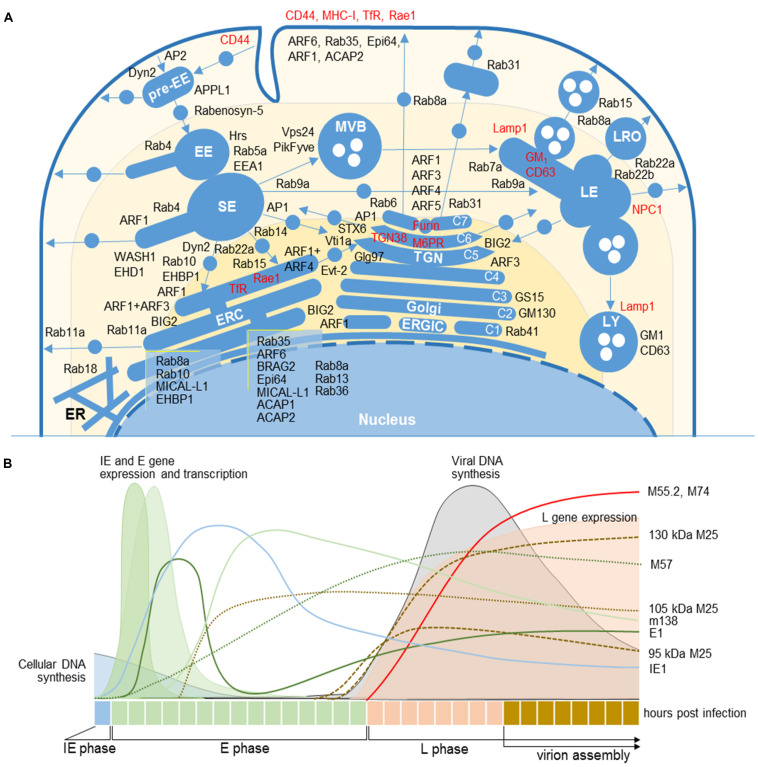
Cellular and MCMV markers used in this study. **(A)** Subcellular distribution of host-cell markers in membranous organelles indicates major sites of their retention or activation/recruitment to membranes (For references see Supplementary Table S2). Markers that circulate within the membranous system are labeled in red. EE, early endosome; ER, endoplasmic reticulum; ERC, endosomal recycling compartment; ERGIC, endoplasmic reticulum-Golgi intermediate compartment; LE, late endosome; LRO, lysosome-related organelles; LY, lysosome; MVB, multivesicular body; SE, sorting endosome; TGN, trans-Golgi network. C1-C7, cisternae of the Golgi stack. **(B)** Organization of the MCMV life cycle and expression kinetics of MCMV genes that encode proteins of interest for this study. The schematic presentation is based on the published data (Scrivano et al., 2010; Marcinowski et al., 2012; Kutle et al., 2017). IE, immediate early phase; E, early phase; L, late phase; 11/2-column fitting image.

To characterize membranous organelle reorganization, we used a selected set of membranous organelle markers for immunofluorescence staining and confocal analysis at four time-points after infection with MCMV. We used 64 cellular markers that specifically characterize compartmentalization of membranous organelle systems with focus on markers that can dissect subsets of the endosomal system and the Golgi. The sites of their principal localization or activation in unperturbed cells are defined by the literature survey and depicted in Figure 1A. Detailed description and classification of markers are provided in Supplementary Table S2 and Supplementary Figure S7.

Markers that are integral membrane components (i.e., transferrin receptor or MHC class I proteins) and migrate with the membrane flow (Type A markers, Supplementary Figure S7) display the entire trafficking route and primary retention localization in the cell. Markers that are cytoplasmic proteins which transiently recruit to membranes display the specific membrane domain and imply biochemical reaction that is behind their recruitment and activation (i.e., the lipid composition of the membrane, interacting effectors, or a slot in the regulatory cascade). These markers either migrate between two steady-state compartments (Type B markers) or transiently recruit to localized sites at membranes and do not migrate with the membrane flow (Type C markers). The interactome maps of these markers are not complete, but those that are available (i.e., https://www.genecards.org/and https://thebiogrid.org/) suggest complex interacting networks and require more sophisticated approaches in the reconstruction of the biochemistry of membranous domains. Thus, for the analysis in this study, we followed known functional interactions published in the literature (listed in Supplementary Tables S2, S3).

### Analysis of the AC

The composition of the MCMV AC was analyzed by double or triple immunofluorescence staining of 64 cellular markers and three viral proteins that are required for the cytoplasmic envelopment of MCMV. This approach has been used in several studies of HCMV AC (Homman-Loudiyi et al., 2003; Cepeda et al., 2010; Fraile-Ramos et al., 2010; Das and Pellett, 2011). We used monoclonal antibodies (mAbs) against murine M55 (glycoprotein B) and M74 (glycoprotein O) gene products, two well-known components of the virion envelope (Kattenhorn et al., 2004), and against M25, the most abundant component of the virion tegument (Kattenhorn et al., 2004; Kutle et al., 2017). Previously characterized expression kinetics of these proteins, which is schematically depicted in Figure 1B, was confirmed by transcriptome (Marcinowski et al., 2012; Juranić Lisnić et al., 2013), biochemical, and immunofluorescence analysis (data not shown). Using visualization of these three proteins, we confined the AC boundaries, as described in HCMV studies.

In fibroblasts and fibroblast-like Balb 3T3 cells, the immediate-early (IE) and early (E) phase of infection is executed within the first 16 h (Figure 1B). Thus, for the analysis of PrAC, membranous organelle reorganizations in the E phase of infection, we performed studies on cells at 6 hpi, the earliest time with consistently observed landmarks of membranous system reorganization (Karleuša et al., 2018), and at 16 hpi, a time when the PrAC is fully developed (Lučin et al., 2018). The AC was analyzed in the late (L) phase, 30 and 48 h post-infection (p.i.). The L phase is initiated by viral DNA replication followed by the expression of late genes, including structural components of the virion envelope and the tegument (Marcinowski et al., 2012). The assembly of virion progeny was observed 20–24 hpi, and the first peak of released extracellular virions was detected between 24 and 48 hpi (Bosse et al., 2012). As an indicator of the infection in the E phase, we used immunofluorescence visualization of immediate-early 1 (IE1; m123) and M57, two MCMV proteins expressed in the IE and E phase of infection (depicted in Figure 1B; Marcinowski et al., 2012).

To avoid unspecific capture of Ab reagents on infected cells, we used Δ138-MCMV for infection, a recombinant virus devoid of m138 gene which encodes a protein with Fc-receptor properties in the E phase of infection (Crnković-Mertens et al., 1998), as described in our previous studies (Ilić Tomaš et al., 2010; Karleuša et al., 2018; Lučin et al., 2018).

Using 64 cellular markers and five viral proteins, we performed localization analysis in three dimensions (3D), as described in the HCMV study (Das and Pellett, 2011). Although localization analysis displayed a high level of complexity, as expected, we classified expression patterns according to primary/principal localization of cellular markers, which is presented in Figure 7. The 3D colocalization analysis across the entire z-stack of confocal images and detailed colocalization analysis of typical patterns in the L-phase of infection is presented in Figures 3–5 and Supplementary Figures S8–S18. Staining patterns of uninfected cells, as well as images displaying multiple cells and patterns within infected cell populations, are presented in Supplementary Figures S8–S16.

### Expression Pattern of MCMV-Encoded Proteins

In the first set of experiments, we extensively characterized the expression pattern of virion structural proteins (M55, M74, and M25) that should localize within the AC. M55 and M74 build MCMV envelope glycoprotein complexes and are expected at membranous organelles of the secretory pathway (Scrivano et al., 2010). M25 is expressed in two main forms: 105 kDa M25 that is expressed in the nucleus during the E and L phase of infection, and 130 kDa M25 that is expressed only in the L phase of infection in both the nucleus and cytoplasm (Kutle et al., 2017), and incorporates into virions as a dominant tegument protein (Kattenhorn et al., 2004; Kutle et al., 2017).

**FIGURE 2:**
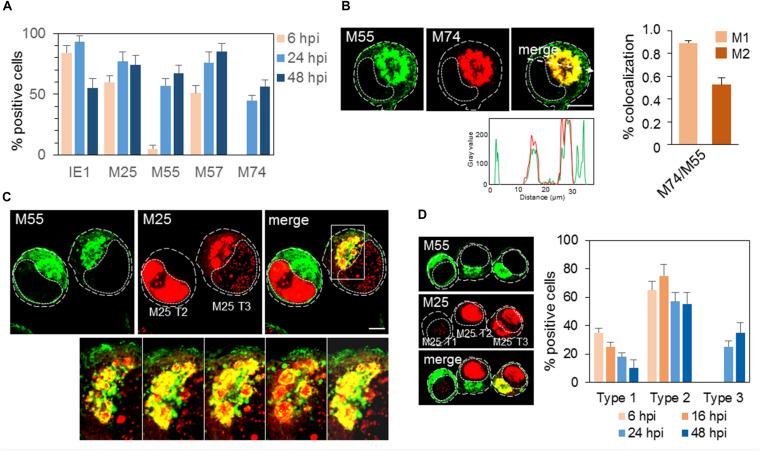
Expression pattern of MCMV-encoded envelope glycoproteins and tegument protein. **(A)** Percentage of cells that express MCMV-encoded proteins at 6, 24, and 48 hpi determined by immunofluorescence staining with specific mAbs (Supplementary Table S1). The data represent mean ± SEM from four experiments. **(B)** *Colocalization analysis of M55 and M74*. Cells were infected with Δm138-MCMV (MOI 10) and 48 hpi and stained for expression of M55 and M74. The pixel overlaps coefficients of M74 with M55 (M1), and M55 with M74 (M2) measured across the Costes-algorythm thresholded z-stacks of confocal images are shown in the right. Data represent mean ± SEM per cell (*n* = 20). Fluorescence intensity profiles along white dashed lines are shown below images. **(C)** *Colocalization analysis of M55 and M25*. Cells treated as described above were stained with specific mAbs (Supplementary Table S1) and corresponding isotype-specific secondary Abs. Serial images of the boxed area acquired at higher magnification are shown at the bottom. **(D)** *Patterns of M55 and M25 expression*. Cells stained for M55 and M25 as described in C were classified according to the pattern of M25 expression (M25 T1, T2, and T3) and the percentage of cells that express one of these patterns determined at 6, 16, 24, and 48 hpi. T1, cells that express M55 but not M25; T2, cells that express M55 and M25 in the nucleus; and T3, cells that express M55 and either M25 in both nucleus and cytoplasm or only in the cytoplasm. The data represent mean ± SEM from three experiments. Cell borders are indicated by fine dashed lines and nuclei by fine dotted lines. Bars, 10 μm. 2-column fitting image.

M55 protein was detected at 6 hpi as a cytoplasmic signal in a small number of cells, presumably due to the staining of M55 incorporated with virions during infection, and massive expression was initiated at 16–17 hpi and later (Figure 1B). At 24 and 48 hpi, approx. 60–70% of cells (Figure 2A) displayed a typical perinuclear staining pattern of M55 in the bell- or ring-formed cytoplasmic cisternal/tubular structure that surrounded the empty juxtanuclear area (Figure 2B). Also, M55 was found in tubulo-vesicular structures in the cortical area of the cell, including subplasmalemmal accumulation and expression at the cell surface (Figure 2B). A similar perinuclear pattern was also presented after staining with mAb to M74, which almost entirely colocalized with M55, but with minimal cortical and subplasmalemmal distribution (Figure 2B). M74 staining was not detected in the E phase of infection, and it was present in approximately half of the cells at 48 hpi (Figure 2A).

The progression through the E phase of infection was monitored by expression of IE1 and M57 proteins, which displayed strong nuclear staining at 1–2 and 6 hpi, respectively (data not shown). At 6 hpi, 80–90% of cells expressed IE1, and 50–60% expressed M57 (Figure 2A). At 24 hpi, almost all cells expressed IE1 and approx. 80% expressed M57, whereas, at 48 hpi, IE1 was detected in 50–60% of cells, which is consistent with decreased transcription at the late stages of infection (Marcinowski et al., 2012). On the other hand, M57 was detected in 80–90% of cells at 48 hpi (Figure 2A). These quantifications, together with quantification of M55 and M74 expression, demonstrate that progression through the E phase and consequently through the L phase is not synchronous throughout the entire cell population. Therefore, many cells that were positive for IE1 and M57 did not express M55 and M74. Even more, a fraction of cells that expressed M55 did not express M74 (Figure 2A).

As expected, M25 was expressed in the E phase in approx. 60% of cells (Figure 2A), displaying a nuclear punctate pattern (Kutle et al., 2017), and in the L phase (24 and 48 hpi) in approx. 3/4 of cells. At 30–48 hpi, M25 displayed either pattern of nuclear or cytoplasmic expression and various intermediary forms (Figure 2C). The nuclear pattern was present throughout the MCMV replication cycle (T2 in Figures 2C,D), whereas cytoplasmic forms were present only in a fraction (30–40%) of infected cells (T3 in Figures 2C,D). The enlarged cytoplasmic aggregates of M25 in the perinuclear area appeared to be enveloped by viral glycoprotein-loaded membranous structures, as exemplified by colocalization analysis of M25 and M55 in Figure 2C. Almost all cells expressing cytoplasmic M25 also expressed M55, indicating that only 30–40% of cells can develop AC with proper distribution of viral components required for final assembly. A small fraction of cells that developed M55-loaded perinuclear structure did not express cytoplasmic M25 (T1 in Figure 2D).

Altogether, expression patterns of two glycoproteins (M55 and M74) and the tegument protein (M25) display the site of tegumentation and secondary envelopment and thereby confine the AC boundaries.

### Contribution of Classical Steady-State Organelles to the AC

**FIGURE 3:**
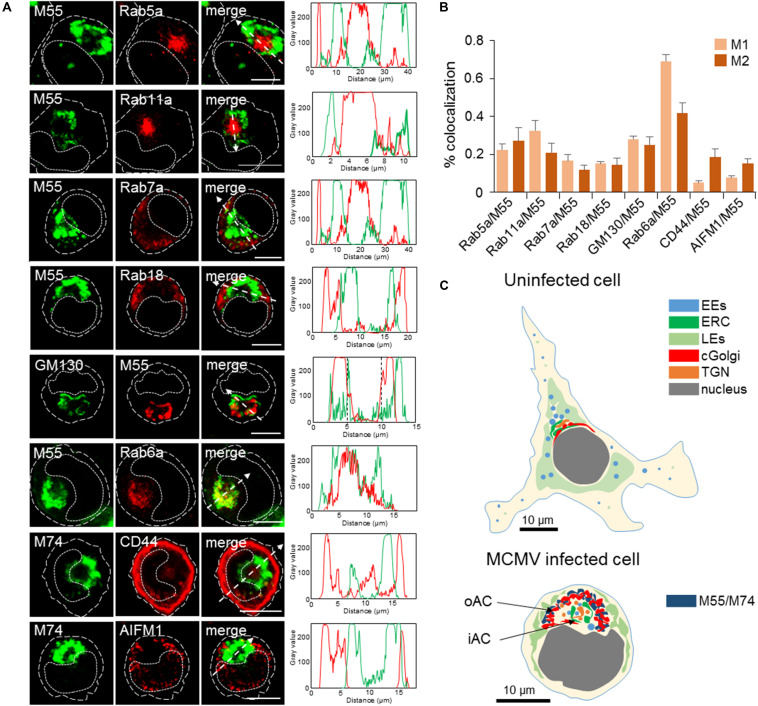
Subcellular distribution patterns of classical steady-state organelles at 48 hpi with MCMV. **(A)** The steady-state organelles were visualized using Ab reagents to cellular proteins that characterize EEs (Rab5), the ERC (Rab11), LEs (Rab7), trans-Golgi and TGN (Rab6), cis/medial-Golgi (GM130), ER (Rab18), cortical endomembrane system (CD44), and mitochondria (AIFM1). The sites of intracellular accumulation of viral envelope glycoproteins were visualized using mAb reagents to MCMV proteins M55 and M74. Antibody reagents used are listed in Supplementary Table S1, and each marker described in Supplementary Table S2. Colocalization analysis was performed by plotting fluorescence intensity profiles along white dashed lines and shown in the right column. Cell borders are indicated by fine dashed lines and nuclei by fine dotted lines. Bars, 10 μm. **(B)** Colocalization analysis of classical steady-state organelle markers with M55/M74 based on M1/M2 coefficients of pixel overlap. Data represent mean ± SEM per cell (*n* = 8–12). **(C)** Schematic presentation of major organelle localization in uninfected and MCMV-infected Balb-3T3 fibroblasts. The cytoplasmic area of an uninfected cell can be divided into three zones according to the distribution of membranous organelles: cortical, perinuclear, and juxtanuclear. The cortical zone of the cell can be confined by visualization of CD44 distribution, perinuclear zone by visualization of LEs using LBPA as a marker, and juxtanuclear zone by simultaneous visualization of LEs (using LBPA as a marker) and internalized TfR (see Supplementary Figure S9). The bottom image presents a schematic outline of cytoplasmic area zones in MCMV infected cells at 48 hpi. 11/2 -column fitting image.

The contribution of classical steady-state organelles to the AC was analyzed by colocalization of MCMV glycoproteins (M55/M74) and host-cell markers on MCMV-infected cells at 48 h p.i. We used antibodies against host-cell proteins that are recruited to the large membrane domains of classically defined membranous organelles and do not significantly migrate during the membrane flow (type C markers). Figure 3 presents images of individual cells acquired at high magnification, representative colocalization analysis by fluorescence intensity profiles along the selected area (dashed arrow), and true degree of colocalization across z-stacks as defined by Menders overlap coefficients. Images of infected cells acquired at lower magnification, as well as staining patterns in uninfected cells, are presented in Supplementary Figure S8.

In infected cells, Rab5 and Rab11 were concentrated in a large aggregate of vesiculo-tubular elements within the M55-loaded ring-shaped structure (Figure 3A). Rab5 and Rab11 coincided with M55 at the boundaries of the ring and in discrete M55-loaded structures within the ring (Figure 3A), typically resulting in the modest degree of colocalization (Figure 3B). These data demonstrate that the inner area of the AC concentrates EE- and the ERC-derived membranous structures which do not accommodate a significant fraction of viral glycoproteins. In contrast, Rab7- and Rab18-positive structures were found outside of the M55-loaded ring (Figure 3A), indicating that LE- and ER-derived membranous elements are dislocated to the outer area of the cell.

GM130-positive cis- and medial-Golgi cisternae were vacuolized and intertwined with the M55-loaded membranes (Figure 3A), resulting in a modest degree of colocalization (Figure 3B). Rab6-stained membranous structures displayed a similar pattern and highly overlapped with M55-loaded membranes (Figure 3A), indicating that viral structural glycoproteins are retained in the TGN cisternae and TGN-derived membranous structure. However, Rab6 and M55 never fully overlapped (Figure 3B), and M55 was found on membranous structures outside and within the ring of membranous structures confined by Rab6 labeling (Figure 3A). This pattern suggests that viral structural glycoproteins are also retained in the Golgi cisternae before the TGN, as well as in post-TGN membranous structures.

CD44 did not overlap with M55-loaded (not shown) and M74-loaded (Figures 3A,B) perinuclear ring, indicating that membranous cortical system, which can be confined by CD44 staining (Supplementary Figure S9), does not contribute in the building of the perinuclear membranous aggregate representing the AC. Finally, mitochondria of MCMV infected cells appeared enlarged and dislocated from the perinuclear area confined by M55-loaded and EE- and ERC-derived membranous structures (Figures 3A,B).

Altogether, the analysis using markers of the classical steady-state organelles demonstrates that MCMV extensively reorganizes membranous system of the cell, which is schematically presented in Figure 3C. These reorganizations are similar to those described for HCMV infection (Cepeda et al., 2010; Das and Pellett, 2011; Tandon and Mocarski, 2012). Thus, the entire area of the infected cell, which is confined by viral glycoprotein-loaded membranous organelles, can be considered as the AC. For further analysis, we designated the ring area containing cis/medial-Golgi and viral envelope glycoprotein-loaded trans-Golgi stacks as the outer AC (oAC) and the area within the ring as the inner AC (iAC).

### The Late Endosomal System Does Not Redistribute Into the AC of MCMV Infected Cells

Although studies on HCMV infected cells demonstrated translocation of some LE markers into the iAC (Jean Beltran et al., 2016), our analysis of Rab7 expression in MCMV infected cells (Figure 3) indicates that LE-derived membranes do not contribute to the AC. Given that LEs are a heterogeneous population of endosomal subsets and/or membranous domains that can be subdivided according to the Lamp1 and Rab7 (Humphries et al., 2011), CD63 (Laulagnier et al., 2011), NPC1 (Garver et al., 2000) and MLN64 (van der Kant et al., 2013) distribution, we analyzed the expression pattern of these markers in MCMV infected cells. Also, we analyzed the expression of ganglioside M1 (GM1), which labels a subset of internal membranes of LEs, and Rab27b, which displays post-LE intermediates. None of these markers localized within the AC (Supplementary Figure S10), indicating that the membrane intermediates that build AC are not derived from the LE system or post-LE intermediates known as lysosome-related organelles (LRO). Importantly, Lamp1 and CD63 are integral membrane components that circulate throughout the entire endosomal system. Their absence in the AC indicates that there is either very little trafficking of LE-derived membranes through the AC or their transit through the AC is very fast.

### Inner AC Contains a Large Number of Membranous Elements

Electron microscopy studies of both MCMV (Buser et al., 2007; Bosse et al., 2012) and HCMV (Homman-Loudiyi et al., 2003; Tandon and Mocarski, 2012; Schauflinger et al., 2013; Archer et al., 2017) AC demonstrated that the vacuolar rim of Golgi stacks surrounds centrally accumulated aggregate of numerous vesicular, vacuolar and tubular structures. Most of these structures have a diameter of 50–200 nm. Since the volume of the iAC area is relatively large (63–523 μm^3^), half of it can accommodate 7.8 to 62.5 thousand of membranous entities with a diameter of 200 nm, which can correspond to 150–600 entities through the equatorial section of the cell in confocal images.

**FIGURE 4:**
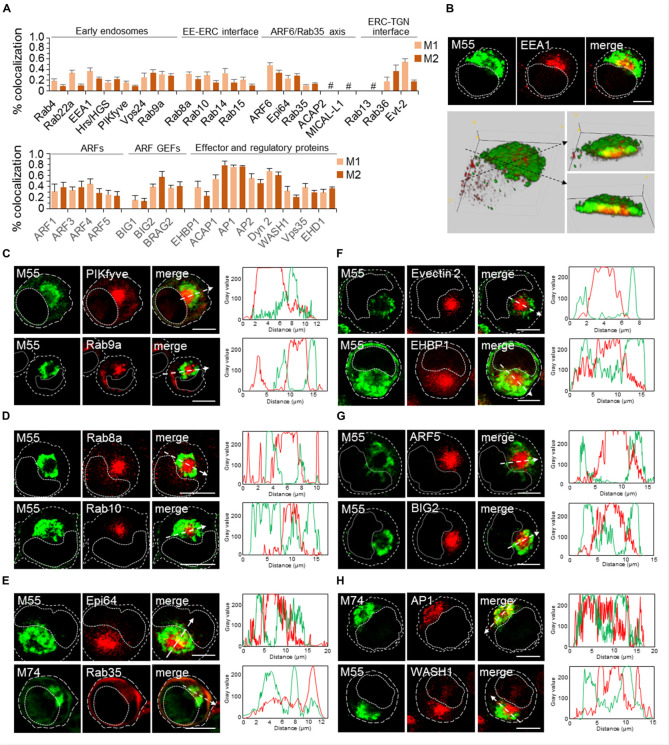
Analysis of markers of early endosomes (EE), endosomal recycling compartment (ERC), and effector proteins that control scission at the EE-ERC-TGN interface at 48 hpi with MCMV. **(A)** 3D colocalization analysis of endomembrane markers with M55 protein based on M1/M2 coefficients of pixel overlap, determined on Costes-algorythm thresholded z-stacks of confocal images. Markers were visualized using Ab reagents (Supplementary Table S2), and the sites of intracellular accumulation of viral envelope glycoproteins were visualized using mAb reagents to M55. Antibody reagents used are listed in Supplementary Table S1. Data represent mean ± SEM per cell (*n* = 6–10). #, not available due to the low signal in infected cells. **(B)** Example of 3D reconstruction of the AC based on expression of Rab5 and M55 protein in 48-h infected MCMV cells. Upper panel presents confocal slices obtained through the focal plane and lower panel 3D reconstruction of the entire z-stack (14 slices) using Image J Volume Viewer plugin. Images shown on the right (* and **) present the view across the section of stack indicated by dashed arrows. **(C–H)**) Subcellular distribution of representative markers. Complete experiments are shown in the Supplementary Material (Supplementary Figures S11–S15). Examples of the subcellular distribution of markers of EEs **(C)**, ERC **(D)**, ERC-associated Arf6/Rab35 axis **(E)**, ERC-associated effectors proteins **(F)**, ARF system **(G)**, and EE/ERC-associated scission machinery **(H)**. Shown are images through the focal plane and colocalization analysis by plotting fluorescence intensity profiles along white dashed lines. Cell borders are indicated by fine dashed lines and nuclei by fine dotted lines. Bars, 10 μm. 2-column fitting image.

**FIGURE 5:**
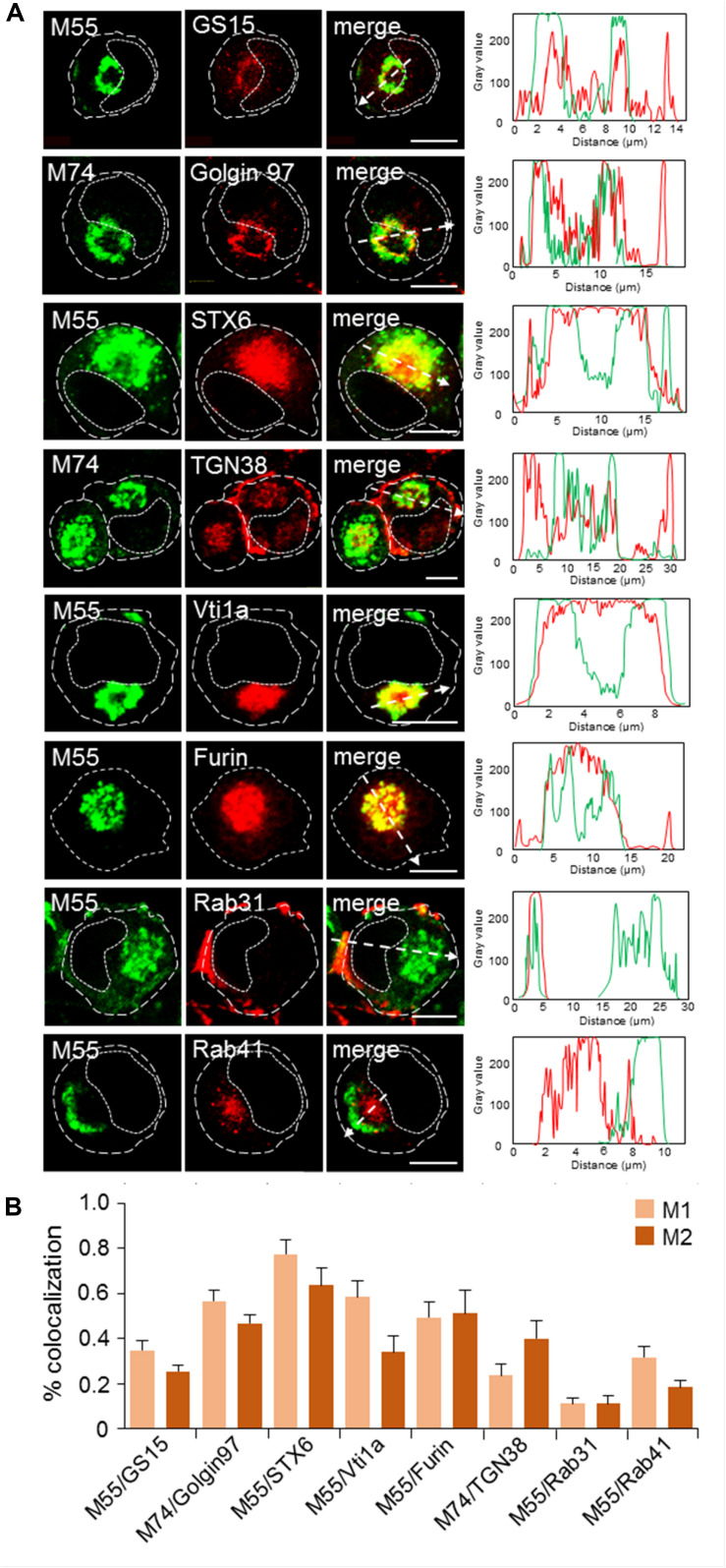
Subcellular distribution patterns of Golgi markers at 48 hpi with MCMV. **(A)** Immunofluorescence analysis. Golgi-derived membranes were visualized using Ab reagents to cellular proteins that characterize various stages of Golgi maturation (Supplementary Table S2), and viral envelope glycoproteins were visualized using mAb reagents to M55 and M74. Antibody reagents used are listed in Supplementary Table S1. Colocalization analysis was performed by plotting fluorescence intensity profiles along white dashed lines and shown in the right column. Cell borders are indicated by fine dashed lines and nuclei by fine dotted lines. Bars, 10 μm. **(B)** Colocalization analysis of M55/M74 and classical steady-state organelle markers based on M1/M2 coefficients of pixel overlap measured across the Costes-algorythm thresholded z-stacks of confocal images. Data represent mean ± SEM per cell (*n* = 6–10). 1-column fitting image.

Given that the iAC area may contain a mixture of membranous entities derived from EE, ERC, and TGN (Figure 3), we extended further analyses to markers that can dissect subsets and biogenesis of EE-, ERC-, and TGN-derived membrane domains and intermediates. The analysis is summarized in Figures 4, 5. Figure 4 presents a 3D colocalization analysis of 33 markers that act at EEs and the ERC, representative 3D reconstruction of the AC, and images of markers that may describe the iAC area. The detailed imaging analysis of all markers is presented in Supplementary Figures S11–S15.Figure 5 presents the colocalization analysis and distribution of markers of the Golgi system.

### Accumulation of the Terminal Stages of EE Differentiation in the iAC

The EE system is generated by the recruitment of Rab5 to stable membranous compartments localized at the cell periphery, known as pre-EEs. It undergoes through a series of transformations which develop tubular recycling domains and vacuolar domain that mature into late endosomes (LEs) (rev. in Naslavsky and Caplan, 2018). The pre-EE system did not contribute to the formation of AC, as tested by APPL1 and Rabenosyn 5 staining (IF images not shown, the result is summarized in Figure 7), and MCMV infection did not redistribute cortical RE, as demonstrated by CD44 staining (Figure 3). Also, Rab4-controlled tubular endosomal network did not contribute to the AC since Rab4-positive endosomes are dislocated toward the periphery (Supplementary Figures S11, S12) and did not colocalize with M55 (Figure 4A).

Other EE markers analyzed mainly localized at membranes of the iAC area, as exemplified by the equatorial plane presentation (upper panel in Figure 4B) and 3D reconstruction of stacked images (lower panel in Figure 4B) of EEA1. M55-loaded membranes surrounded the cluster of EEA1-positive membranes within the iAC area, and a certain degree of colocalization was present at the boundaries and with M55-positive entities within the iAC (Figure 4B) which is detected in quantitative analysis (Figure 3B). The same pattern was identified for almost all EE markers that are over-recruited to the iAC membranes (Figure 4A).

Given that Rab5a-positive membrane accumulated at the iAC (Figure 3A), we checked the presence of several markers that act downstream in the Rab5 cascades. Rab22a, known to builds regulatory cascade with Rab5a and maintains Rab5a at endosomal membranes (Zhu et al., 2009), was also highly recruited at the iAC membranes (Supplementary Figures S11, S12), indicating prolonged activation of Rab5a at the iAC membranes. High recruitment of EEA1 within iAC (Figure 4B and Supplementary Figure S12) suggests prolonged maturation of Rab5a-enriched structures. This conclusion is confirmed by increased recruitment of Hrs and Vps24 (Supplementary Figures S11, S12), type C markers that do not display distinct structures in uninfected cells (Supplementary Figure S12). High recruitment of EEA1 and Hrs/HGS at the iAC membranes also indicates enrichment in PI3P, whereas increased recruitment of PIKfyve (Figure 4C and Supplementary Figure S12) and Vps24 (Supplementary Figures S11, S12) suggests the enhanced development of the PI(3,5)P2 domain, prolonged activation of the vacuolar protein sorting (VPS) pathway, and the retention of the reverse topology budding machinery at the iAC membranes (Schöneberg et al., 2017).

Altogether, the analysis of EE markers suggests that iAC is built of Rab5-controlled vacuolar domains of EEs and that MCMV infection alters trafficking through the EE system at the final stages of EE maturation, retards exit from EEs, and expands of EE-derived membranous that has the potential of the reverse-topology budding.

### Accumulation of EE-TGN Intermediates in the iAC

In MCMV infected cells, Rab9a was highly recruited in the inner AC, although a significant fraction of Rab9a-positive membranes was found outside the AC (Figure 4C and Supplementary Figure S12). These patterns suggest altered maturation of Rab9-positive EE-derived intermediates that are retained at the iAC and completely segregated from outer Rab9-positive membranes that can be associated with LEs. Namely, Rab9a can display a subset of LE membranes that are distinct from Rab7 subset, LE-derived transport intermediates before fusion with TGN (Barbero et al., 2002), and EE membranes that regulate transport between EEs, TGN, and LEs (Kucera et al., 2016). Thus, the accumulation of Rab9a membranes within the iAC (Figure 4C) also suggests an alteration of trafficking between EEs and TGN.

### Dysregulation of Membrane Flow at the ERC in MCMV Infected Cells

Over-recruitment of Rab11a at the iAC (Figure 3A) also suggests dysregulation of the ERC in MCMV infected cells. The ERC involves heterogeneous subsets of relatively large tubular RE and many small transport intermediates (Xie et al., 2016) that are functionally linked to EEs, PM, TGN, and LEs (Goldenring, 2015). Recent studies indicate that the ERC is usually composed of Rab11a-and Rab8a-positive, and at least one expandable ARF6/Rab35-positive subset of membranes (Kobayashi and Fukuda, 2013). In contrast to Rab11a, Rab8a- and ARF6/Rab35-positive subsets of membranes are not expanded to the stage of the steady-state organelles in the juxtanuclear area of uninfected Balb 3T3 cells (Supplementary Figure S13). However, in MCMV infected cells, both Rab8a (Figure 4D) and ARF6 (Supplementary Figures S11C, S14B) were highly enriched within the iAC area. Together with Rab11a, these data suggest that MCMV infection dysregulates membrane flow at the entire ERC.

### Delayed Biogenesis of the ERC and Accumulation of EE-ERC Intermediates

The biogenesis of the ERC involves either relocation of maturing EEs or budding and fission of intermediates from EEs that fuse with stable ERC membranes (Naslavsky and Caplan, 2018). These processes could be controlled by small GTPases Rab15 (Strick and Elferink, 2005), Rab10 (Liu and Grant, 2015), and Rab14 (Linford et al., 2012), as well as regulatory proteins EHD1, dynamin 2 and WASH1 (Naslavsky and Caplan, 2018).

In uninfected cells, very little Rab15, Rab10, and Rab14 were found in the juxtanuclear area (Supplementary Figure S13). In contrast, in MCMV infected cells, Rab10 (Figure 4D and Supplementary Figure S14A) and Rab15 but not Rab14 (Supplementary Figures S11B, S14A) were highly recruited to the iAC area, suggesting for expansion of and delayed maturation of Rab10- and Rab15-controlled EE-ERC intermediates. Additionally, all three regulatory proteins involved in the biogenesis of the ERC, EHD1, dynamin 2, and WASH1, were also highly recruited to the iAC area (Figure 4H and Supplementary Figures S11E, S15). These data suggest their prolonged retention at EE membranes and delayed maturation of EEs into ERC.

### Delayed Exit From the ERC

Exit from the Rab11-domain of the ERC toward the PM can be regulated by recruitment of Rab8a (Homma and Fukuda, 2016), whereas reciprocal recruitment of ARF6 and Rab35 (Klinkert and Echard, 2016) within the ERC determines the cascade-like recruitment of downstream Rabs (Rab8a, Rab10, Rab13, Rab36) (Kobayashi et al., 2014) as well as exit from the ERC toward LEs and the TGN. The direction of the outgoing flow from the ERC can also be regulated by activation of Rab-to-ARF cascades (D’Souza et al., 2014), which involve BIG2-mediated activation and recruitment of ARF1 and ARF3 for the recycling to the PM (Volpicelli-Daley et al., 2005; Kondo et al., 2012) or ARF1 and ARF4 for retrograde trafficking to the TGN (Nakai et al., 2013).

As described above, both Rab8a and ARF6 were highly enriched at membranes of the iAC, suggesting that MCMV infection delays maturation of Rab8a- and ARF6-positive domains and consequently exit from the ERC. The over-recruitment of ARF6 at the iAC was associated with the over-recruitment of Epi64 (Figure 4D and Supplementary Figure S15), an effector of activated ARF6, which indicates the overactivation od ARF6 at ERC-derived membranes within the iAC. Consistent with this observation, Rab35 (Figure 4D and Supplementary Figure S15), small GTPase that is in reciprocal relation with activated ARF6, and its effectors ACAP2 and MICAL-L1 (Klinkert and Echard, 2016) were absent from the iAC (Supplementary Figures S11C, S14B). Subsequently, we also examined the expression of Rab35 downstream effectors, Rab8a, Rab13, and Rab36 (Kobayashi et al., 2014; Klinkert and Echard, 2016). Although Rab35 was absent, Rab8a and Rab36 were highly recruited to membranes of the iAC (Figure 4D and Supplementary Figures S11B, S14A), suggesting that these Rabs are recruited to the Rab35-independent parts of the endomembrane system within the iAC and that the maturation of outgoing membranes at the ERC is delayed in MCMV infected cells. High enrichment of Rab8a suggests accumulation of membranes with a delayed exit from Rab11a domain toward the cell surface, whereas high enrichment of and Rab36 suggests a delayed exit from the ERC toward the TGN. The delay in maturation of intermediates at the ERC-to-TGN route was further confirmed by a high recruitment Evectin-2 (Figure 4F and Supplementary Figure S15), known to drive retrograde transport from the ERC to the TGN (Matsudaira et al., 2015).

The expansion of membrane domains that are subvisible in uninfected cells (i.e., ARF6-, Rab10-, Rab15-, Rab36-, and Evectin-2-positive domains) suggests that CMV infection retards the domain conversion and thereby expands membrane intermediates. Altogether, analysis of the ERC markers indicates that CMV infection highly reorganizes the ERC and the interface between EEs and the ERC, as well as to ERC and TGN, resulting in the accumulation of expanded subsets of ERC membranes and subsets of intermediates that mediate transport out of the ERC.

### The iAC Is Highly Tubular

EM studies demonstrated many tubular elements within the iAC (Buser et al., 2007; Bosse et al., 2012). Tubulation is the property of membrane flow at EEs, ERC, and TGN, and the extent of tubulation reflects the membrane dynamics at these compartments. Analysis of the ERC markers (Figure 4) demonstrated that many host-cell factors associated with tubulation are over-recruited at membranes of the iAC, including Rab8 and ARF6.

The initiation of tubular domains at EEs and the ERC is associated with regulated activation of the ARF system. Thus, we further analyzed the recruitment of major components of the ARF system in MCMV infected cells. Both, ARF GEFs that may act at EEs and ERC (BIG1, BIG2, and BRAG2) as well as class I (ARF1) and class II (ARF4 and ARF5) ARFs were over-recruited to membranes of the iAC (Figure 4G and Supplementary Figures S11D, S14C,D), suggesting that expanded EE- and ERC-derived intermediates are highly tubulated. Most of these components demonstrated substantial colocalization with M55 (Figure 4A), especially at the border of iAC area, suggesting that ARF system is also over-activated at M55-loaded TGN membranes.

In addition to the ARF system, adaptor protein (AP) complexes are also associated with the initiation of tubular extension. AP1, known to initiate membrane exit at EEs, ERC, and TGN, highly colocalized with M74 (Figure 4A) and was recruited to membranes of the oAC but also at M74-negative membranes of the iAC (Figure 4H). To our surprise, AP2 was also highly recruited to the iAC membranes (Supplementary Figures S11E, S15). Since AP2 acts at the PM in concert with EHD2 protein, the finding of high recruitment of EHD2 at the iAC (data not shown) suggest more extensive dysregulation of membrane flow within the iAC. These data, together with over-recruitment of the ARF system, indicate that many membranes within the iAC are tubular with delayed maturation of tubular extensions and prolonged recruitment of tubulation machinery.

Dynamin-2, WASH1, and EHD1, three host-cell systems that control the scission of tubular elements at EEs and the ERC, were also highly recruited at iAC membranes (Figure 4H and Supplementary Figure S11E) indicating also prolonged budding at the EE and ERC membranes within the iAC. Dynamin 2, known to act also at the TGN, highly colocalized with M55 (Figure 4A). Although the function of WASH1 has been reported to be strictly linked to the retromer complexes, our analysis in MCMV infected cells suggests that retromer function, as shown by visualization of its component Vps35 (Supplementary Figures S11E, S15) is not always associated with WASH1. These data indicate that delayed maturation of tubular elements within the iAC is associated with dysregulated scission.

Altogether, our analysis suggests that MCMV infection strongly affects the maturation of tubular domains within the iAC.

### Contribution of the Golgi and TGN in the AC

In uninfected Balb 3T3 cells, the Golgi system is organized in cisternal stacks around the nucleus (Supplementary Figure S16). In MCMV infected cells, the cis-, medial-, and trans-Golgi were vacuolized, fragmented and displaced from the nucleus to form the outer ring of the AC, as demonstrated by the cis-Golgi marker GM130 (Figure 3A), the medial and trans-Golgi marker GS15 (Figure 5A), and the trans-Golgi and trans-Golgi-TGN interface marker Golgin 97 (Figure 5A). GM130- and GS15- labeled cisternae, highly intertwined with distinct M55/M74-loaded compartments, as demonstrated by moderate colocalization (Figures 3B, 5B, respectively). In contrast, a substantial fraction of M74 colocalized with Golgin 97 (Figure 5B) but also localized in intertwining Golgin 97-negative cisternae (Figure 5A), indicating that viral glycoproteins load the trans-Golgi. Thus, cis-, medial-, and trans-Golgi cisternae (C2-C6 cisternae) form the oAC.

As already demonstrated in Figure 3, viral glycoproteins highly colocalized with the TGN marker Rab6, indicating that TGN cisternae also contribute to the oAC. However, a substantial fraction of Rab6-positive membranes was also found in the iAC (Figure 3A and Supplementary Figure S8), suggesting that MCMV infection reorients a part of the TGN toward the cell center. To analyze this observation further, we analyzed the distribution of STX6 and Vti1a, two type B markers that form the tSNARE complex involved in the post-TGN transport toward EEs and the ERC, and thereby display TGN-derived membranes at the EE-RE-TGN interface (Glick and Nakano, 2009). As demonstrated in Figure 5, membranes enriched in these markers highly colocalized with M55/M74 at the oAC but also a substantial fraction of these membranes accumulated at the iAC devoid of M55/M74, indicating that MCMV infection redistributes and expands membrane intermediates of the EE-ERC-TGN interface at the iAC area.

Given that the EE-ERC-TGN interface is derived from C7 Golgi cisternae (Mogelsvang et al., 2004), we further examined three markers that can display exit events at the late TGN. TGN38, a type-A marker known to circulate through the TGN-PM-RE-TGN route, localized at the vacuolar structures adjacent to M55/74-loaded cisternae, enlarged subplasmalemmal structures and in discrete tubular structures at the iAC (Figure 5A and Supplementary Figure S16). Furin, another type-A marker that circulates the TGN-PM- EE/LE route (Thomas, 2002) and loads different domains of C7 cisternae than TGN38 (Nokes et al., 2008; Boal and Stephens, 2010), was highly enriched in M55/M/74-loaded compartments but also in membrane compartments that build iAC (Figure 5 and Supplementary Figure S16). The distribution of furin in the oAC is consistent with the known role of furin in the posttranslational processing of HCMV gB (Vey et al., 1995). Rab31, a small GTPase known to control anterograde exit from the TGN toward PM was highly recruited to the peripheral membrane system and did not redistribute into the iAC (Figure 5 and Supplementary Figure S16). These data, together with the accumulation of STX6 and Vti1a at the iAC, indicate that MCMV infection does not affect anterograde trafficking exit from C7 but rather retrograde entry into C7 cisternae, resulting in accumulation of EE-ERC-TGN intermediates in the iAC.

In addition to the expansion of intermediates of the late Golgi membrane flow in the iAC area, MCMV infection also reorganizes another side of the Golgi interface. Namely, Rab41 (Rab6d) which is known to organize membrane movements at the interface between the intermediate compartment (IC) and the cis-Golgi (Goud et al., 2018) was also highly recruited at membranes within the iAC (Figure 5 and Supplementary Figure S16) and did not colocalize with viral glycoproteins (Figure 5B).

Altogether, the analysis of the Golgi system demonstrates that oAC is mainly build by the C2-C7 Golgi cisternae, whereas the iAC is composed of membrane intermediates derived at the interface the Golgi and post-Golgi linker compartments (EE-ERC-TGN and cis-Golgi-IC; Saraste and Prydz, 2019) that are reoriented toward the cell center.

### The Architecture of the AC Is Established in the Early Phase of MCMV Infection

To set up a temporal analysis of the AC development, we examined whether the basic architecture of the AC is established in the absence of viral structural envelope and tegument proteins, before their expression. The study of the expression pattern of M25, M55, and M74 (Figure 2) demonstrated that only 30–40% of cells develop full AC architecture, and our previous study (Karleuša et al., 2018) demonstrated several landmarks of the endosomal system reorganization in the E-phase of infection. Given that reorganized cis/medial-Golgi was a prominent feature of the oAC (Figure 3), we explored what the earliest time point in the MCMV replication cycle in which the primary form of the AC (pre-AC, PrAC) is developed. We analyzed the development of the primary AC form using GM130 as a marker of oAC and Rab10 as a marker of EE-ERC interface reorganization within the iAC.

**FIGURE 6:**
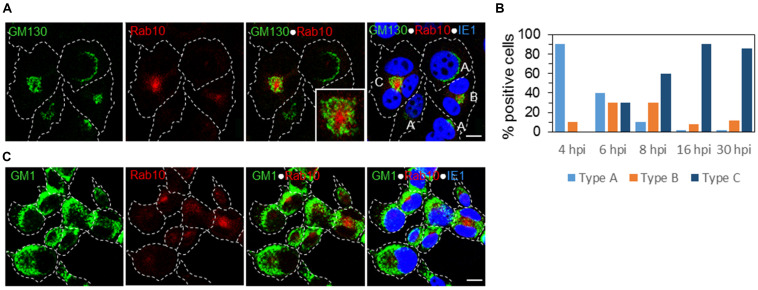
The earliest landmarks of membranous organelle rearrangements in the course of MCMV infection. The cells were infected with Δm138-MCMV (MOI 5), and intracellular distribution of the cis-Golgi marker (GM130), LE marker (GM1), EE/ERC marker (Rab10), and MCMV immediate-early protein (IE1) was analyzed by triple immunofluorescence and confocal microscopy. **(A)** Three patterns of GM130 and Rab10 staining were detectable at 6 h p.i.: A, represents a typical distribution of the Golgi stacks close to the nucleus and weak or almost undetectable recruitment of Rab10; B, represents recruitment of Rab10 with a displacement of the GM130-positive membranes: and C, represents fully developed reorganization of juxtanuclear organelles with a ring-like redistribution of the GM130-positive membranes surrounding large juxtanuclear accumulation of membranes that recruit Rab10. **(B)** Percentage of cells demonstrating the three patterns in the course of MCMV infection. **(C)** Relation of the juxtanuclear Rab10-positive membranes to LEs at 6 h p.i. Cell borders are indicated by fine dotted lines and nuclei by fine dashed lines. Insert represents the boxed area acquired at higher magnification. Bars, 10 μm. 2-column fitting image.

At 6 hpi, we identified three patterns of expression: A, infected cells with the Golgi stacks around the nucleus without recruitment of Rab10 similar to uninfected cells; B, infected cells with displaced Golgi and a small aggregate of Rab10 vesicles; and C, infected cells with an expanded perinuclear aggregate of Rab10 surrounded by vacuolized and fragmented Golgi cisternae (Figure 6A). These changes did not involve LEs, as demonstrated by the simultaneous staining of GM1 and Rab10 (Figure 6C). Roughly, each pattern was present in 1/3 of infected cells (Figure 6B). Pattern B was detected in a small number of cells at 4 hpi (Figure 6B), suggesting that these perturbations are initiated 4–5 h after infection. The development of the pattern C progressed through the E phase of infection (at 8 hpi almost 60% of cells with pattern C), and at the end of the E phase (16 hpi), 90% of infected cells (expressing IE1) demonstrated pattern C (Figure 6B). The same proportion is maintained at 30 hpi (Figure 6B), the time when the AC is fully developed.

This analysis suggests that the unlinking of the Golgi ribbon associated with the reorganization of the post-Golgi linker compartments (Saraste and Prydz, 2019) is the earliest event in the biogenesis of the AC. The basic architecture of the AC is established in the E phase of infection (4–5 hpi), indicating that it is driven by MCMV-encoded E genes and does not require L gene expression, including loading with viral tegument and envelope proteins.

### The AC Is Initiated in the Early Phase of Infection by a Reorganization of the EE-RE-TGN Interface

**FIGURE 7:**
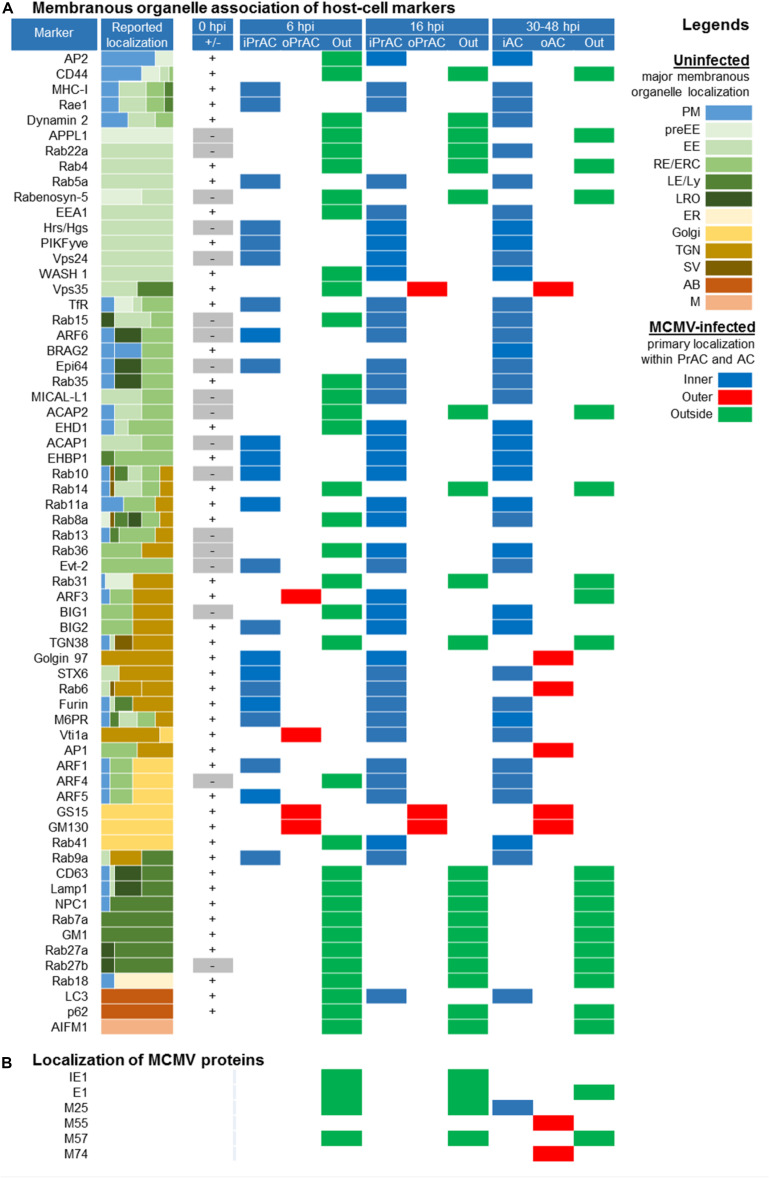
Spatial distribution of membrane markers in the reorganized membranous organelles of the Pre-Assembly Compartment (PrAC) and Assembly Compartment (AC) of MCMV infected cells. **(A)** Membrane markers are sorted according to the reported localization in the membranous system of the uninfected cell (Supplementary Table S2). The column Reported localization represents the approximate relative distribution of the markers in membranous organelles published in the literature (represented by different colors in the legend on the right). Column 0 hpi (±) represents the absence (–) or the presence (+) of the expression pattern at the distinguishable steady-state organelles of uninfected Balb 3T3 cells (Supplementary Figures S8–S10, S12, S13, S15, S16). Columns 6 and 16 hpi, as well as column > 30, represents the presence and primary (major) distribution of markers at reorganized membranous structures of the infected cells classified as inner and outer PrAC or AC, respectively (legend MCMV-infected). The primary distribution of markers was based on the simultaneous immunofluorescence staining of markers with either GM130 in the E phase of infection (6 and 16 hpi) or viral structural glycoproteins (M55 and M74) in the late phase of infection (>30 hpi). **(B)** Primary localization of MCMV proteins in the course of MCMV infection determined by immunofluorescence analysis. 2-column fitting image.

Using the established landmarks of the PrAC and the AC, we continued a spatial and temporal analysis of membranous system rearrangements with 64 markers during the E-phase of infection. Although the intracellular distribution demonstrated a higher degree of complexity, we categorized all the markers according to the major sites of expression within the infected cell: inner (iPrAC and iAC), outer (oPrAC and oAC), and out of (Out) the area confined by the GM130 in the PrAC and M55/M74 in the AC (Figure 7). Of particular interest was the analysis of type B and C markers that do not recruit at the abundant steady-state organelles in uninfected cells (marked as gray boxes in the column 0 hpi in Figure 7). These markers display domains or membranous intermediates that are either small in size or short-lived, and their presence in uninfected cells was confirmed by Western-blot (see Figure 10) and, in some cases, by the enhancement of immunofluorescence signal (data not shown).

At the end of the E phase of infection (16 hpi), a similar but not identical pattern was established as at 30 and 48 hpi (Figure 7A). Markers of the EE, ERC, and some markers of the TGN displayed membranous structures in the iPrAC, markers of the Golgi and TGN in the oPrAC, and markers of ER, LEs, LROs, mitochondria, and peripheral endomembrane system localized outside the PrAC (Figure 7A). This analysis demonstrates that a majority of membranous organelle reorganization present in the AC is achieved during the E phase of infection. However, TGN-derived elements, at least those controlled by ARF3 and Rab6, appear to continue to change in the L phase of infection, after expression of L-phase genes and viral structural proteins. ARF3, which mainly localized at the cell periphery at 30–48 hpi, at 16 hpi mainly localized at the iPrAC (Figure 7A). TGN markers, Golgin 97 and Rab6, which mainly localized at the oAC at 30–48 hpi, at 16 hpi mainly localized at iPrAC (Figure 7). Also, ARF6-associated membranous organelle functions undergo significant changes at later stages of infection. Nuclear accumulation of ARF6 and its GEF BRAG2 observed at 48 hpi (Supplementary Figure S11D) was rarely observed at earlier stages of infection.

In contrast to the composition of PrAC established at 16 hpi, which mostly mirrors the one established in the AC, analysis at 6 hpi displays the earliest set of membranous organelle reorganization. In addition to the displacement of the cis/medial Golgi stacks, as demonstrated by the pattern of GM130 staining (Figure 6), displacement to oPrAC was observed for GS15, Vti1a, and ARF3, but not for TGN38, Golgin97, Rab6, and STX6, all of which mainly remained in the iPrAC (Figure 7A).

Given that all of these Golgi/TGN markers are associated with well-defined organelle structures in uninfected cells, perturbation of their localization indicates the reorganization of the existing steady-state organelles. A more indicative of the extensity of reorganization was an analysis of type B and C marker that did not associate with distinct membranous structures in uninfected cells, such as Rab10, EHBP1, ACAP1, ARF6, Epi64, Evectin-2, and Rab36. All of them were highly recruited to membranes of the iPrAC at 6 hpi (Figure 7A), and together with increased recruitment of Rab11a and Rab8a (Figure 7A), suggest an alteration of membrane flow at the entire ERC. High recruitment of ACAP1 and EHBP is consistent with the over-activation of Rab10 since these two proteins are well-known effectors of Rab10. Similarly, high recruitment of ARF6-effector Epi64 and the absence of Rab35, MICAL-L1, and ACAP2 in the iPrAC is consistent with over-activation of ARF6 (Figure 7).

Enhanced recruitment of Rab5a and the lack of accumulation of EEA1, WASH1, and Vps35 at 6 hpi (Figure 7A) suggests an alteration of EE dynamics at the later stages of EE maturation. Over-recruitment of Rab10, but not Rab14 and Rab15, at the iPrAC (Figure 7) suggests a delay in maturation or transition of EEs toward the ERC, whereas over-recruitment of Rab9a (Figure 7A) suggests the delay in EE maturation toward the TGN. Enhanced recruitment of Hrs/HGS, PIKfyve, and Vps24 (Figure 7A), three host-cell proteins displaying the VPS pathway, suggest that the delay is in the maturation of EE vacuolar domain. These data indicate that the alterations of the terminal stages of EE maturation are among the earliest alterations during CMV infection.

Consistent with the perturbation of membrane organization at the EE-ERC-TGN interface was also enhanced recruitment of BIG2, ARF1, and ARF5 to the inner PrAC at 6 hpi (Figure 7A), followed by enhanced activation of BIG1, ARF3, and ARF4 at later stages of the E phase (16 hpi, Figure 7A). These changes indicate stepwise over-activation of the entire ARF system within the PrAC and expansion of ARF-dependent tubular domains in the early phase of infection.

Altogether, analysis of MCMV infected cells at 6 hpi demonstrates that unlinking the Golgi ribbon from the post-Golgi linker compartments, delay in maturation of EEs, and delay in membrane flow at the EE-ERC-TGN interface are the earliest events in the AC biogenesis. The expansion of EEs and EE-ERC-TGN intermediates and displacemet of the Golgi ribbon in the PrAC establishes the topology that is also maintained in the fully formed AC. Relocation of pre-Golgi linker compartments, as indicated by a location of Rab41 outside the PrAC at 6 hpi and its relocation to the iPrAC at 16 hpi (Figure 7A), occurs later with the progression of the E-phase.

### The PrAC Phenotype Is Not a Side Effect of Cell Contraction in the Early Phase of Infection

**FIGURE 8:**
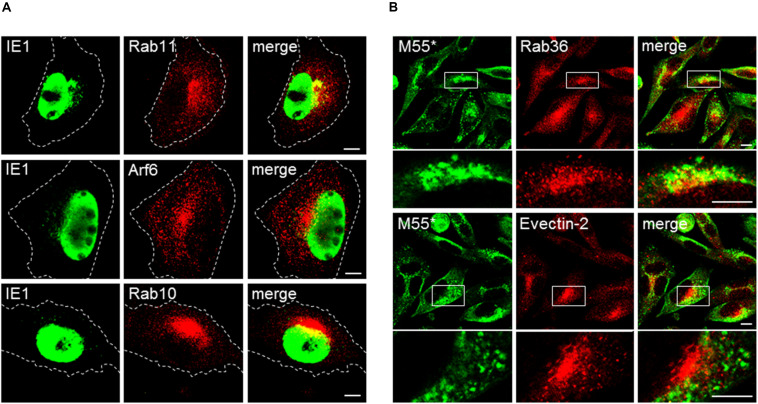
Membranous organelle reorganization in cells infected with the recombinant MCMV with deleted genes that mediate cell rounding. Balb 3T3 cells were infected with Δ9-MCMV (with deleted M23-M26 genes) and analyzed at 16 **(A)** and 30 hpi **(B)** for subcellular localization of selected ERC markers and IE1 and M55 proteins. M55* staining indicates subcellular localization of the M55 gene product and FcR binding by m138/fcr1 gene product, which was not deleted in Δ9-MCMV. Note that fcr1 binds murine IgG_2__a_ but not rabbit IgG. Cell borders are indicated by fine dashed lines. Lower panel images in **(B)** represent the boxed area acquired at higher magnification. Bars, 10 μm. 2-column fitting image.

It is possible that several markers do not display distinct structures in uninfected cells by immunofluorescence microscopy (Figure 7, column 0 hpi) because they are dispersed in the diffuse cytosolic pool of subvisible membranous intermediates. After infection, which is associated with the well-known cytopathogenic effect of cell rounding and contraction (Figure 3C), these markers may be concentrated in a smaller volume and thereby may appear as perinuclear aggregate within PrAc and AC. Although the images and fluorescence intensity profiles in Figures 3–5 demonstrate that several markers associate with sufficiently large membranous entities; still, a substantial number of markers within the iAC display enlarged amorphous structure at immunofluorescence images (Figure 3–5 and Supplementary Figures S11, S12, S14, S15). To resolve this issue, we performed analysis after infection with Δ9-MCMV, a recombinant virus with a deletion of M23-M26 genes that do not develop significant cell rounding after infection. Cells infected with Δ9-MCMV did not establish round shape at 16 and 30 hpi (Figure 8) but displayed juxtanuclear accumulation of Arf6 and Rab10 at 16 hpi (Figure 8A), and Rab36, Evectin-2, Epi64, Vps24, and BIG2 at 30 hpi (Figure 8B and Supplementary Figure S17). These markers did not display sufficiently large structures in the juxtanuclear area of uninfected cells (Supplementary Figure S13) and may be considered as landmarks of membranous organelle reorganization during MCMV infection. All these markers were present at distinct punctate structures and tubular elements in the juxtanuclear area of Δ9-MCMV infected cells (Figure 8 and Supplementary Figure S17). Therefore, the phenotype characteristic for PrAC and AC is not the side effect of cell rounding and contraction, which appears early in the infection.

### MCMV Does Not Reorganize the EE-ERC-TGN Interface by Alteration of Host-Cell Transcriptional Activity

The over-recruitment within PrAC of several small GTPases that endogenously do not display clear membranous organelles suggests either reorganization of existing intermediates of the EE-ERC-TGN interface or their upregulation and expansion of membranous domains into a new organelle structure. Although the quantitative relations between membrane-associated and cytoplasmic pools of these proteins have not been precisely established, many studies suggest that the cytoplasmic pool is rather small (Supplementary Table S2). A recent study demonstrates that the cell limits the size of the cytoplasmic pool of GDP-bound Rab proteins (inactive) by continuous ubiquitin-mediated degradation (Takahashi et al., 2019). Thus, the size of the membrane-associated pool is maintained by the rate of their recruitment, degradation, and gene expression.

**FIGURE 9:**
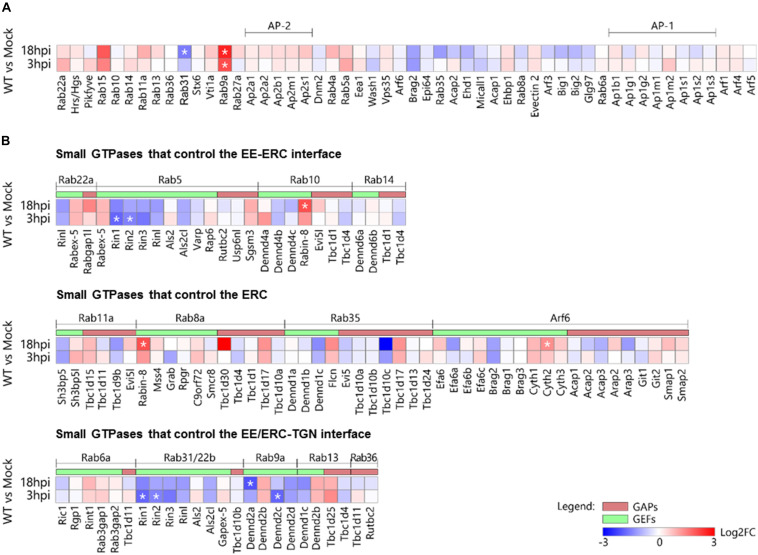
Effect of MCMV infection on the expression of the genes encoding membranous organelle markers and regulatory proteins (GEFs and GAPs) that control membrane flow at the EE-ERC-TGN interface. **(A)** Genes encoding type B and C markers. **(B)** Genes encoding GEFs and GAPs that regulate small GTPases at the EE-ERC-TGN interface. The data represent the fold change (log2) of gene expression at the beginning (3 hpi) and the end (18 hpi) of the early phase of MCMV infection relative to the mock-infected cells. Statistically significant genes are designated with an asterisk (*). 2-column fitting image.

Given that the cytoplasmic pool of regulatory elements is small and is limiting factor in organelle growth (Goehring and Hyman, 2012), over-recruitment of small GTPases to membranes of the PrAC may be associated with rapid depletion of their limiting pool and the compensatory enhancement of their gene expression. Thus, we analyzed transcriptional activity of all cellular genes that encode markers used in this study at 3 hpi, before the onset of membranous organelle rearrangements, and 18 hpi, at the end of E phase of infection before the intracellular accumulation of viral structural proteins (Figure 9A and Supplementary Figure S19). The host-cell transcriptome was analyzed on DC2.4 cells that displayed a similar pattern of membranous organelle rearrangement (Supplementary Figure S19) and compared with the previously available transcriptome data (Marcinowski et al., 2012; Juranić Lisnić et al., 2013).

Although transcriptome analysis demonstrated consistent upregulation and downregulation trends during the E phase of infection (Figure 9A), almost all these alterations were not significant. This observation is particularly important for those EE-RE-TGN interface host-cell factors that significantly increased membrane-associated pool (over-recruitment) and demonstrated the trend of transcriptional downregulation in MCMV infected cells. The only observed exception was Rab9a, which was rapidly upregulated after MCMV infection, and Rab31, which was downregulated at 18 hpi (Figure 9A). The rapid increase in Rab9a transcription may be associated with the increase in the membrane-associated pool within the inner PrAC (Figure 7). Therefore, we concluded that the transcriptional upregulation of small GTPases of the EE-ERC-TGN interface is not a significant mechanism whereby MCMV remodels the membranous system.

The increased membrane-associated pool of small GTPases that regulate the EE-ERC-TGN interface may be a result of an alteration of the membrane-associated pools of regulatory proteins that determine the extent of their recruitment. To explore this option, we analyzed transcriptional activities of all known genes encoding GEFs and GAPs for small GTPases that act at the EE-ERC-TGN interface. Among all analyzed genes, we observed significant upregulation of the only Rabin8 and Cytohesin-2 at 18 hpi (Figure 9B). Rabin8 is known GEF for Rab8, and its upregulation may be associated with the increased recruitment of Rab8 to the inner PrAC, which is present at 16 hpi but not at 6 hpi (Figure 9). Cytohesin-2 is a GEF for ARF1 and ARF6 that act at the cell periphery and, thus, its overexpression at 18 hpi cannot explain the increase in the membrane-associated pools of ARF proteins within the inner PrAC at 6 hpi (Figure 7). Significant downregulation of Rin1 and Rin2 gene expression (Figure 9B) may suggest an alteration of EE maturation at the cell periphery but not at the inner PrAC since their products act as GEFs for Rab5 and Rab31/22b at peripheral endosomes. Similarly, observed downregulation Rab9a GEF-encoding genes, DENND2a and DENND2b, in the E phase of infection (Figure 9B) does not correlate with the increase of Rab9a membrane-associated pool within the inner PrAC.

Altogether, alteration of expression of genes encoding key host-cell factors that control membrane flow at the EE-ERC-TGN interface cannot explain the membranous organelle perturbation that leads to the development of PrAC in the E-phase of infection.

### MCMV Does Not Reorganize the EE-ERC-TGN Interface by Alteration of Intracellular Level of Small GTPases

**FIGURE 10:**
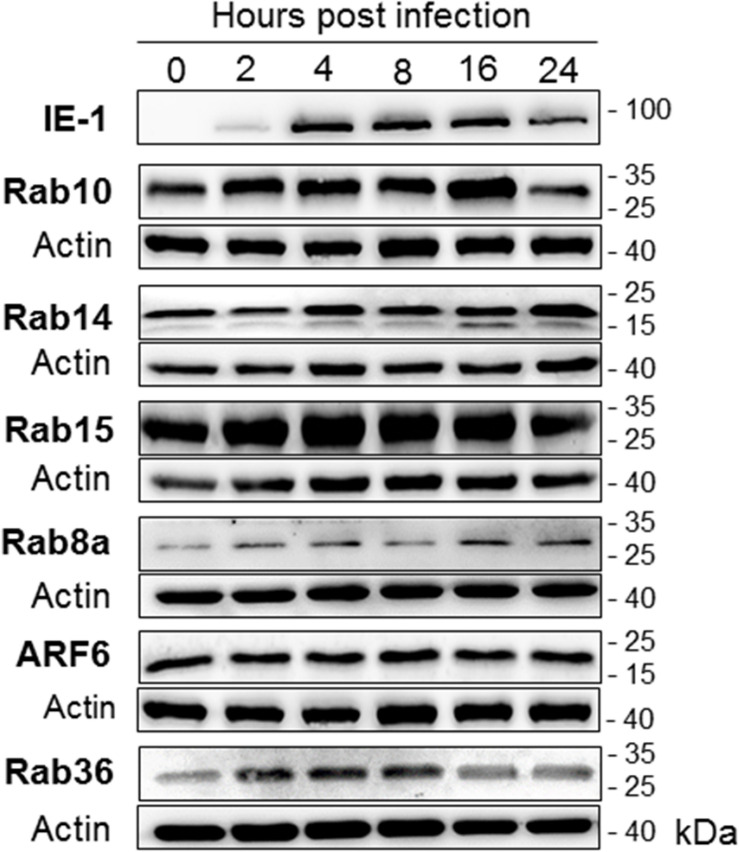
The expression level of host-cell small GTPases that act at the EE-ERC-TGN interface. Western-blot analysis was performed using lysates of uninfected (0 hpi) and MCMV-infected (Δm138-MCMV, MOI 10) Balb 3T3 cells at various stages of infection (2–24 hpi). The kinetic expression of each small GTPase was determined by simultaneous analysis of MCMV IE1 protein (shown is a representative image) and β-actin at the same membrane. Shown are images of representative experiments out of 3–5 experiments. 1-column fitting image.

The membrane-associated pool of small GTPases may be dysregulated by alteration of their degradation during MCMV infection. Thus, we analyzed the intracellular amount of several small GTPases that act at the EE-ERC-TGN interface, especially those that are not recruited to distinct membranous organelles in uninfected cells. Although Rab8A, Rab10, ARF6, Rab15, and Rab36 showed weak immunofluorescence staining in uninfected cells (Supplementary Figure S13) and strong immunofluorescence signal in the inner PrAC (images not shown, summary results in Figure 7) and the AC (Figure 4D and Supplementary Figures S11B, S14A,B), their amount did not significantly change during the early phase of MCMV infection (Figure 10). Similarly, the amount of Rab14, which is not highly recruited to membranous structures within the PrAC or AC (Supplementary Figure S11B), was even increased in infected cells (Figure 10). These data confirm that MCMV infection does not upregulate Rab proteins of the EE-ERC-TGN interface to increase their membrane-associated pool, neither by increased transcription nor by inhibited degradation.

Together, immunofluorescence studies of host-cell factors recruitment (Figures 3–7 and Supplementary Figures S8–S18), quantitative analysis of host-cell transcriptome (Figure 9 and Supplementary Figure S19) and protein expression (Figure 10) indicate that MCMV infection uses a mechanism which alters recruitment/de-recruitment dynamics of host-cell regulatory factors at membranous intermediates of the EE-ERC-TGN interface in order to initiate the development of the AC.

## Discussion

In this study, we present spatio-temporal phenotyping of the AC of beta-herpesvirus infected cells and demonstrate that MCMV infection reorganizes the interface between EEs, endosomal recycling compartment (ERC), and the trans-Golgi network (TGN). The reorganization was initiated very early in the infection, indicating that MCMV encoded early genes drive the establishment of the new organelle structure (PrAC), which evolves into a sizeable cytoplasmic structure known as the AC. The profound effect of MCMV infection on the membranous system was displayed as over-recruitment of several host-cell factors that regulate membrane flow at the EE-ERC-TGN interface without significant alteration of their gene expression, indicating that early-gene products of MCMV target recruitment mechanisms and regulatory cascades of membrane-shaping host-cell factors. The phenotyping of the new organelle structure represents a basis for further studies of beta-herpesvirus assembly as well as unclear physiological interactions at the EE-ERC-TGN interface.

### CMV Infection Expands Tubular Domains and Membrane Intermediates

Our study demonstrates that MCMV induces similar set membranous organelle reorganizations as human CMV (Das et al., 2007; Krzyzaniak et al., 2009; Cepeda et al., 2010; Das and Pellett, 2011; Close et al., 2018a; Taisne et al., 2019). As in HCMV studies, a cluster of reorganized membranous organelles confined by reorganized Golgi stacks and membranous elements loaded with viral envelope glycoproteins was denoted the AC (rev. in Tandon and Mocarski, 2012), and the cluster confined by the reorganized Golgi stacks in the early phase of infection was denoted the PrAC (Taisne et al., 2019). The inner area of the PrAC and AC in MCMV infected cell, which occupies a volume of 63–523 μm3, is filled with a large number of vesicular and tubular membranous elements (Maninger et al., 2011). These membranous elements bear host-cell factors that regulate membrane domain dynamics at EEs, ERC, and TGN. Most of these host-cell factors are poorly recruited to membranes in uninfected cells, indicating that MCMV infection expands membrane domains that are intermediates at the EE-ERC-TGN interface. For example, over-recruitment of Rab10 and Rab15 indicates the expansion of EE-to-ERC intermediates, over-recruitment of ARF6, Epi64, Rab13, Rab36, and Evt2 suggests an expansion of ERC-to-TGN intermediates, and over-recruitment of Stx6 and Vti1a suggests an expansion of TGN-to-EE/ERC intermediates (for references see Supplementary Table S2).

The inner PrAC and AC membrane domains are highly tubular, as demonstrated by over-recruitment of several host-cell factors that regulate membrane budding at EEs (i.e., WASH1, dynamin, AP1, EHD1, and EHBP1), ERC (i.e., Rab8a, Rab10, BIG2, ARF1, ARF4, ARF5, and ARF6), and TGN (i.e., BIG1, ARF1, ARF3, and AP1). Vacuolar elements within the inner AC and PrAC are derived mainly by dysregulation of EE maturation, as demonstrated by the accumulation of Hrs/HGS, PikFYVE, and Vps24. These alterations are associated with inhibited endosomal recycling and EE maturation (Ilić Tomaš et al., 2010; Karleuša et al., 2018; Lučin et al., 2018), with several functional consequences, including immune evasion (Lučin et al., 2015). Similar alterations were also recently described in HCMV infection (Hook et al., 2014; Zeltzer et al., 2018). It appears that expanded membranous elements of the inner AC and PrAC extrude the Golgi stacks from the cell center to form the outer ring of the AC and PrAC.

The reorganization of the EE-RE-TGN interface indicates that CMV infection affects multiple pathways and regulatory cascades that regulate membrane flow between these steady-state organelles. Although it is believed that EE cargo is sorted and transported by membrane intermediates to the ERC, it is still not clear whether ERC is just a terminal stage of maturation of EEs after sorting of recycling domains (Naslavsky and Caplan, 2018). Similarly, both EEs and ERC can deliver membranes with cargo to TGN, and TGN to EEs and ERC (Glick and Nakano, 2009). Many host-cell factors that regulate ERC have also been reported to associate with the TGN (Supplementary Table S2). An increasing number of examples suggest that there is no clear boundary between these steady-state organelles and that membranous intermediates create a continuum between these compartments (Glick and Nakano, 2009). For example, Rab11a, a conventional marker of the ERC, has been found in association with the TGN membranes and post-Golgi vesicles (Ullrich et al., 1996), whereas TGN38 and Stx6, conventional markers of the TGN (Bock et al., 1997; Reverter et al., 2014), can be identified in EEs and the ERC (Simonsen et al., 1999). Even more, perturbation of trafficking at the EE-ERC-TGN interface or alteration of cholesterol level in the TGN can result in translocation of these markers to EEs or ERC (Reverter et al., 2014). Almost all the machinery that builds ARF system, including ARF-GEFs (BIG1 and BIG2) and ARF proteins (ARF1, ARF3, ARF4, and ARF5), has been reported to associate with the functions of the ERC (Kondo et al., 2012; Nakai et al., 2013) and the TGN (Ishizaki et al., 2008; Boal and Stephens, 2010). All these recruitments occur around the cell center between highly intertwined, steady-state compartments that communicate with each other. Thus, it is challenging to create experimental settings with physiological expression levels of regulatory host-cell factors that can distinguish interface between these organelles, especially when organelle typing is based on migrating markers such as TfR, furin, M6PR, or TGN38. Obviously, better resolution of membranous organelles around the cell center is essential for the characterization of their interface. For example, the use of PC12 (Kobayashi and Fukuda, 2013; Homma and Fukuda, 2016) and COS-1 (Matsudaira et al., 2015) cell lines in several studies provided significant insights into the function of the ERC. Therefore, in addition to a better understanding of the AC biogenesis, MCMV infection could represent a useful model for studying the EE-ERC-TGN interface under the physiological expression level of regulatory host-cell factors.

### The Golgi Reorganization Seems to Be First

The Golgi fragmentation and displacement, as also demonstrated in our previous study (Karleuša et al., 2018), is one of the earliest landmarks of membranous system reorganization during MCMV infection, evident already 4–5 h after infection. The Golgi fragmentation is also apparent in HCMV infected cells approx. 2 days after infection (Rebmann et al., 2016; Taisne et al., 2019) and may be an initial step in the formation of the AC, as suggested by Rebmann et al. (2016). It may result in unlinking the Golgi non-compact region and dysregulation of linker compartments (the ERC and the IC), as it occurs during mitosis and cell migration (Saraste and Prydz, 2019). Although our study was focused on EE-ERC-TGN interface markers, over-recruitment of Rab41 at membranes of the inner AC, a small GTPase that acts at the IC-Golgi interface (Liu et al., 2016), suggests that both linker compartments could be sequentially reorganized during CMV infection. In accordance with this could be the observation of enhanced recruitment of LC3, which correlates with increased recruitment of Rab41 in the inner AC, in MCMV (this study), and HCMV infected cells (Taisne et al., 2019).

### LE/LRO System Is Out of the AC in MCMV Infected Cells

In contrast to the observations in HCMV infected cells (Cepeda et al., 2010; Fraile-Ramos et al., 2010; Das and Pellett, 2011; [Bibr B36][Bibr B77]), MCMV infection does not relocate CD63 and Lamp1, LE and LRO markers, to AC and PrAC. In this study, we also tested several markers that may define LE subsets, and none of them was found at the inner AC and PrAC area, indicating that MCMV infection segregates EE-ERC-TGN from the LE/LRO system. This was demonstrated by the segregation of Rab9a-positive membranes into two subsets, one within the inner AC and one outside the AC. Rab9a has been shown to act at the EE-TGN interface (Kucera et al., 2016) and to define a subset of LEs (Barbero et al., 2002).

### A Mixture of Membrane Intermediates May Be Required for Secondary Envelopment

Heterogeneous membrane domains of the AC derived at the EE-ERC-TGN interface may ensure a proper environment for the final stage in CMV assembly, the secondary envelopment. Lessons from alpha-herpesviruses suggest that the secondary envelopment does not occur at the site of a high concentration of viral glycoproteins but rather at an endosomal compartment (Johns et al., 2014). The existing knowledge about beta-herpesvirus composition suggests that membranous compartment appropriate for secondary envelopment should ensure several properties. First, membranes should provide a proper biophysical environment and machinery required for budding membrane away from the cytoplasm and fission of the membrane into the virion envelope. This “reverse topology” mechanism (Schöneberg et al., 2017) requires PI3P domains, conversion of PI3P into the PI(3,5)P2 by PIKFyve, activation of the ESCRT pathway, and termination by recruitment of Vps24 (Votteler and Sundquist, 2013). All of these requirements are the property of EE membranes, which are highly enriched within the PrAC/AC and appear to be significantly retarded, as indicated by the over-recruitment of Hrs/HGS, PIKFyve, and Vps24, suggesting that PrAC/AC accumulate reverse-topology permissive membranes. Second, CMV envelope proteins should be sorted by cargo-sorting mechanisms and transported via the TGN-to-EE route from PI4P-rich TGN membranes (Marcelić et al., unpublished) toward reverse-topology permissive membranes to meet a proper environment, as described for alpha-herpesviruses (Johns et al., 2014). Third, topological features of a membranous compartment for successful envelopment could require large membrane surface-area-to-volume ratio and association with microtubule and actin tracks that ensure forces for scission required for virion egress. These are properties of tubular domains that characterize ERC and TGN. Fourth, membranes for envelopment should provide an appropriate lipid composition. It is reasonable to believe that MCMV will build a similar lipidome composition of infectious virions as HCMV, including threefold reduction of PS and twofold enrichment in PE (Liu et al., 2011) and that development of the secondary envelopment foci will also include lipidome remodeling. Therefore, the envelopment may be expected at membranes capable of concentrating PS decarboxylase, known to be enriched at TGN (Schuiki and Daum, 2009).

Altogether, none of the EE-, ERC-, and TGN-derived membranous compartments can provide a proper composition for envelopment. Thus, it is possible that, through inhibition of membrane flow at the EE-ERC-TGN interface, CMV infection generates a mixture of membranous intermediates and CMV capsids associate with membranes until they create a proper environment for the envelopment. Alternatively, the diverse tubular membrane domains formed at the EE-ERC-TGN interface that undergo slow transition within the inner AC could include intermediary forms that can mix cargo and molecular machinery, and some of these intermediary forms could have an adequate composition for the secondary envelopment. Studies with tagged beta-herpesvirus capsids are essential to address this issue.

### Targeting the General Mechanism of Host-Cell Factors Membrane Recruitment

Host-cell transcriptome (Hertel and Mocarski, 2004; Marcinowski et al., 2012; Juranic Lisnic et al., 2013; Jean Beltran et al., 2016) and proteome (Weekes et al., 2014) analyses demonstrate that CMVs alter the expression of a large number of host-cell factors that regulate membrane flow. These alterations are associated with systemic virus infection-induced effect on host-cell protein translation (McKinney et al., 2014; Weekes et al., 2014), their targeted degradation (Tirosh et al., 2015), and specific targeting by virus-encoded miRNAs (Hook et al., 2014). Our study demonstrates that CMVs could also target membrane recruitment of host-cell factors that build regulatory cascade at the EE-ERC-TGN interface. Although the cascades can be disrupted by targeting the expression level of host cell-factors, our analysis (Figure 9) did not identify significant alterations of host-cell factors used in this study as markers sufficient to cause extensive reorganization. Thus, it is reasonable to believe that MCMV encodes functions that target a general mechanism involved in fine-tuning of membrane flow by affecting membrane recruitment of host-cell factors, such as host-cell factor phosphorylation (Rebmann et al., 2016) or membrane cholesterol (Gudleski-O’Regan et al., 2012) or host-cell factor ubiquitination balance.

The ubiquitination of the endosomal system machinery has emerged as a mechanism that modulates the dynamics and maturation of the EE system (Ramanathan et al., 2013; Hao et al., 2015). The molecular rheostat function based on the fine titration of the ubiquitination by the cooperative action of a ubiquitin (Ub) ligase and a deubiquitinating enzyme (DUB) has been demonstrated in the actin-assembly function of the WASH complex at EEs (Hao et al., 2015). A recent study demonstrated that ubiquitination by multiple Ub ligases and interaction with several DUBs also regulates ERC dynamics (Sakai et al., 2019). Inhibition of an Ub ligase that acts at the ERC (Sakai et al., 2019) or its over-expression (Coumailleau et al., 2004) impairs the segregation of EEs and the ERC, inhibits ERC-to-TGN trafficking, and redistributes a TGN resident protein (TGN46) into the ERC. Spatiotemporal regulation of ubiquitination of host-cell factors that control endosomal tubulation (i.e., EHD1 and MICAL-L1) has been proposed to be essential for segregation of EE and the ERC (Sakai et al., 2019). Thus, the membrane flow at the ERC interface could be regulated by Ub-based molecular rheostats, and CMVs may reshape the EE-ERC-TGN interface by dysregulation of the coordinated function of ERC-associated Ub ligases and DUBs. This can be achieved by modulation of Ub-ligases and DUBs expression or by viral coding proteins with Ub-ligase or DUB function. In the host-cell transcriptome of MCMV infected cells, we did not identify significant alterations of host-cell transcripts that build the Ub-ligation and DUB network (data not shown). On the other hand, both HCMV and MCMV encode at least one protein with DUB activity (UL48 and M48, respectively). Both proteins are expressed in the early phase of infection (Marcinowski et al., 2012; Weekes et al., 2014) and are essential for virus growth (Das et al., 2014; Hilterbrand et al., 2017). Therefore, M48, through its DUB function, could be an MCMV-encoded tool for disruption of the molecular rheostat functions and initiation of EE-ERC-TGN interface reorganization in the early phase of infection. To test this hypothesis, it would be essential to develop a recombinant MCMV with mutated DUB domain of the M48 since the virus without M48 is not viable (data not shown; Hilterbrand et al., 2017).

In conclusion, our study demonstrates that AC of murine and human CMV has a similar organizational structure and that studies on MCMV can contribute to a better understanding of the AC of beta-herpesviruses. The MCMV model is characterized by the short time required for the onset of the AC (6–8 h), which may be advantageous in the host-cell factor perturbation studies. The set of landmarks of the PrAC and AC presented in our study could improve understanding the biogenesis of the AC, and could contribute to the identification of the secondary envelopment site, host-cell factors that are essential for secondary envelopment and virion egress, as well as virus-encoded functions that drive these processes.

## Data Availability Statement

The accession numbers for the RNA sequencing data can be found in the Supplementary Material.

## Author Contributions

NJV, LjK, HML, GBZ, MM, and SLJ performed the immunofluorescence experiments. GBZ, VP, and KG performed the Western-blot experiments. IB generated the monoclonal antibodies to MCMV proteins. BL performed the transcriptome analysis; HML, GBZ, and PL analyzed and processed the data. PL wrote the manuscript. All authors contributed to the article and approved the submitted version.

## Conflict of Interest

The authors declare that the research was conducted in the absence of any commercial or financial relationships that could be construed as a potential conflict of interest.

## Footnotes

1 http://rsb.info.nih.gov/ij/plugins/track/jacop.html

2 https://www.bioinformatics.babraham.ac.uk/projects/fastqc/
