# Supplementary material for: Cytomegalovirus Generates Assembly Compartment in the Early Phase of Infection by Perturbation of Host-Cell Factors Recruitment at the Early Endosome/Endosomal Recycling Compartment/Trans-Golgi Interface
Source: Front Cell Dev Biol. 2020 Sep 11;8:563607. doi: 10.3389/fcell.2020.563607 (PMC7516400; doi:10.3389/fcell.2020.563607)
Supplement: Supplementary file 1 [file Data_Sheet_1.PDF]

Supplementary Material

Table S1. List of antibody and ligand reagents used in this study

Target molecule	Reagent	Reference
**Rab proteins**		
**Rab4a**	Rabbit polyclonal (Cell Signaling, Cat.No. 2167P)	Lee et al., 2013
**Rab5a**	Rabbit polyclonal (Santa Cruz Biotech; Cat. No. sc28570)<br>Rabbit polyclonal (Cell Signaling, Cat.No. 3547P)	ad sc28570 - Meister et al.. 2014
**Rab6a**	Rabbit polyclonal (Cell Signaling; Cat.No. 9625)	Kawauchi and Nabeshima, 2019
**Rab7a**	Rabbit polyclonal (Cell Signaling; Cat.No. 9367)	Hong et al., 2015; Hubert et al., 2016
**Rab8a**	Rabbit polyclonal (LS Bio; Cat.No. LS-C313375)	-
**Rab9a**	Rabbit polyclonal (Cell Signaling; Cat.No. 5118)	Liu et al., 2012
**Rab10**	Rabbit polyclonal (Cell Signaling; Cat.No. 8127)	Homma and Fukuda, 2016
**Rab11a**	Rabbit polyclonal (Cell Signaling; Cat.No.5589)	Di Matteo et al., 2017
**Rab13**	Rabbit polyclonal (Biorbyt; Cat.No. orb36847)	-
**Rab14**	Rabbit polyclonal (Novus, Cat.No. NBP1-84720)	Goundry et al., 2017
**Rab15**	Rabbit polyclonal (Biorbyt, Cat.No. orb326053)	-
**Rab18**	Rabbit polyclonal (Sigma-Aldrich, Cat.No. SAB4200174)	Qinwei et al., 2016
**Rab22a**	Rabbit polyclonal (LS Bio; Cat.No. LS-C112063)	-
**Rab27a**	Mouse monoclonal IgG_1 (Abcam, Cat.No. ab55667)<br>Rabbit polyclonal (Abcam; Cat.No. ab108983)<br>Sheep polyclonal (R&D Systems, Cat.No. AF7245)	ad ab55667 - Ostenfeld et al., 2014

**Rab27b**	Rabbit polyclonal (Millipore, Cat.No. ABS1026)	Nojima et al., 2016
**Rab31/Rab22b**	Rabbit polyclonal (LS Bio, Cat.No. LS-C186716)	-
**Rab35**	Rabbit polyclonal (Cell Signaling; Cat.No. 9690S)	Dikshit et al., 2015
**Rab41**	Rabbit polyclonal (Biorbyt; Cat.No.orb372858)	-

**Rab effectors, GEFs and GAPs**		
**EPI64**	Rabbit polyclonal (Biorbyt; Cat.No. orb313370)	-
**MICAL-L1**	Mouse monoclonal IgG_1 (Santa Cruz Biotechnology; Cat.No. sc-398397)	Takahashi et al., 2017
**ACAP1**	Rabbit polyclonal (biorbyt; Cat.No. orb29096)	-
**ACAP2**	Mouse monoclonal IgG_1 (Santa Cruz Biotechnology; Cat.No. sc-376150)	-
**Rabenosyn-5**	Rabbit polyclonal (LS Bio, Cat.No. LS-C110401)	-
**Hrs/HGS**	Rabbit polyclonal (LS Bio, Cat.No. LS-B12780)	-
**Vps24**	Rabbit polyclonal (LS Bio, Cat.No. LS-C94080)	-
**PikFYVE**	Rabbit polyclonal (LS Bio, Cat.No. LS-C119339)	-
**APPL-1**	Rabbit polyclonal (Cell Signaling, Cat.No. 3858)	Jung et al., 2017
**EEA1**	Chicken polyclonal (Invitrogen, Cat.No. 40-5700)	Mu et al., 1995; Simonsen et al., 1998; Christoforidis et al., 1999

**ARF proteins and their effectors**		
**ARF1**	Rabbit monoclonal (Abcam; Cat.No. ab32524)	Rocca et al., 2013
**ARF3**	Rabbit monoclonal (Abcam; Cat.No. ab157467)	-
**ARF4**	Rabbit polyconal (Novus; Cat.No. NBP1-55918)<br>Rabbit polyclonal (LSBio; Cat.No. LS-C353895)	-
**ARF5**	Rabbit monoclonal (Abcam; Cat.No, ab125023)	-

**ARF6**	Rabbit polyclonal (Abcam; Cat.No. ab77581)<br>Rabbit monoclonal (Abcam; Cat.No. ab49931)<br>Rabbit monoclonal (Cell Signaling; Cat.No. 5740S)	ad ab77581 - Allaire et al., 2013; Eva et al., 2012<br>ad ab49931 - Eva et al., 2012;<br>ad 5740S - Sakagami et al., 2008
**BIG1**	Rabbit polyclonal (Biorbyt; Cat.No. orb155841)	-
**BIG2**	Rabbit polyclonal (Abcam, Cat.No. ab75001)<br>Rabbit polyclonal (LSBio; Cat.No. LS-C119793)	-
**BRAG2**	Rabbit polyclonal (Abcam, Cat.No – ab122442)	Humphreys et al.,2013

	**Endosomal markers**	
**CD63**	Rat monoclonal IgG_{2b} (MBL International, clone R5G2, Cat.No. D263-3)	Bobrie et al., 2012
**Lamp1**	Rat monoclonal IgG_{2a} (BD Pharmigen, Cat.No. 553792)	Belaid et al., 2014; Garg et al., 2011
**NPC1**	Rabbit polyclonal Abs (LifeSpan BioSciences; Cat.No. LS-B6281).	van der Kant et al., 2013
**GM1**	Alexa Fluor CTxB 555 conjugate (Invitrogen, Cat.No. C34776)<br>Alexa Fluor CTxB 488 conjugate (Invitrogen, Cat.No. C22841)	ad C34776 - Shaw et al., 2006;<br>ad C22841 - Bavari et al., 2002;
**Evectin-2**	Rabbit polyclonal (Biorbyt, Cat.No. orb312792)	-
**AP1 (Anti-adaptin γ)**	Mouse monoclonal IgG_{2a} (BD Biosciences, Cat.No. 610385)	Nag et al., 2018
**AP2 (Anti-adaptin α)**	Mouse monoclonal IgG_1 (BD Biosciences, Cat.No. 610501)	Stevenson et al., 2017
**Vps35**	Rabbit monoclonal (Abcam, Cat.No.ab157220)	MacDonald et al., 2018
**WASH1**	Mouse monoclonal IgG1 (Sigma-Aldrich,Cat.No.SAB4200552)	-
**Dynamin2**	Rabbit polyclonal IgG (Thermofisher scientific, Cat.No. PA5-19800)	Søreng et al., 2018
**LBPA (lysobisphosphatidic acid)**	MAbs, hybridoma 6C4 (mouse IgG1; gift of Dr. J. Gruenberg, University of Geneva, Switzerland).	Chevallier et al., 2008; Brankatschk et al., 2011
**EHBP1**	Rabbit polyclonal (Novus; Cat.No.NBP1-93614)	Simone LC et al., 2013

Endosomal cargo proteins		
**MHC-I**	MAb, hybridoma SF1-1.1.1 (mouse IgG2a) – recognize the heavy chain of fully conformed H2-K^d molecule	Noun et al., 1996
**CD44**	clone IM7, rat	Hiršl et al., 2018
**TfR (CD71)**	Tranferrin Alexa Fluor 555 conjugate (Invitrogen, Cat.No. T35352)<br>Transferrin Alexa Fluor 488 conjugate (Invitrogen, Cat.No. T13342)<br>Monoclonal antibody, clone R17 217.1.3 (rat IgG2a; ATCC TIB 219).	ad T3535 - Razi et al., 2009<br>ad T13342 - Barysch et al., 2010<br>mAb - Mahmutefendić et al., 2011,
**M6PR**	Rabbit polyclonal (Abcam, Cat.No. ab2733; ab8093)	ad ab2733 - Munson et al., 2015; ad ab8093 - Boonyaratanakornkit et al., 2013
**Furin**	Rabbit polyclonal (Abcam, Cat.No. ab3467)	Delestre-Delacour et al., 2017
**RAE1**	Clone Rae1γ.01, mouse IgG1	

Golgi markers		
**GS15**	Mouse monoclonal IgG_1 (BD Biosciences, Cat.No. 610960)	Williams et al., 2014
**GM130**	Mouse monoclonal IgG_1 (BD Biosciences, Cat.No. 610823)	Bagh et al., 2017
**Vti1a**	Mouse monoclonal IgG_1 (BD Biosciences, Cat. No. 611220)	Crawford et al., 2017; Wong and Munro, 2014
**Syntaxin-6**	Rabbit Monoclonal (Thermo Fischer Scientific, Cat.No. MA5-14858)	-
**Golgin 97**	Rabbit polyclonal (Abcam, Cat.No.ab84340)	Doerflinger et al., 2017
**TGN-38**	Rabbit polyclonal (Novus Biological, Cat.No. NBP1-03495)	Fernandez-Fernandez et al., 2017

Autophagic and apoptotic pathways		
**LC3A, B, C**	Rabbit polyclonal (MBL, Cat.No. PM036)	Monel et al., 2017
**LC3B**	Rabbit polyclonal (Cell Signaling Technology, Cat.No. 2775)	Piragyte et al., 2018
**p62**	Mouse monoclonal IgG_{2a} (Novus; Cat. No. H00008878-M01)	An and Harper, 2018
**AIFM1**	Rabbit polyclonal (Cell Signaling, cat no 4642)	Park et al., 2017

Cytoskeleton		
**Actin**	Mouse monoclonal (Millipore, Cat.No. MAB1501)	Treda et al., 2016
**Tubulin**	Rabbit polyclonal (Sigma, Cat.No. T-3526)<br>Mouse monoclonal IgG_1 (Invitrogen, Cat.No. A11126)	ad Sigma T-3526 - Zhang et al., 2009<br>ad Invitrogen A11126 - Cooper et al., 2015

MCMV proteins		
**m123/IE1**	Mouse monoclonal IgG_1; clone CROMA 101 (University of Rijeka Center for Proteomics, Cat. No. HR-MCMV-08).<br>Mouse monoclonal IgG_{2a}; clone IE1.01. (University of Rijeka Center for Proteomics, Cat. No. HR-MCMV-12).	Kučić et al., 2005; Budt et al., 2009
**M112-113/E1**	Mouse monoclonal $IgG_{1,}$ clone CROMA 103 (University of Rijeka Center for Proteomics, Cat. No. HR-MCMV-07)	Kučić et al., 2005; Podlech et al., 2010
**M25**	Mouse monoclonal $IgG_{1,}$ clone M25C.01 (University of Rijeka Center for Proteomics, Cat. No. HR-MCMV-03)	Kutle et al., 2017
**M55.1 (gB)**	Mouse monoclonal $IgG_{2b,}$ clone M55.01 (University of Rijeka Center for Proteomics, Cat. No. HR-MCMV-05)	Liu et al., 2014
**M55.2 (gB)**	Mouse monoclonal IgG_{2a}, clone M55.02 (University of Rijeka Center for Proteomics, Cat. No. HR-MCMV-14).	Yunis et al., 2019
**M57**	Mouse monoclonal, clone M57.02 (University of Rijeka Center for Proteomics, Cat. No. HR-MCMV-6).	Lenac Rovis et al., 2016
**M74**	Mouse monoclonal IgG1, clone M74.01 (University of Rijeka Center for Proteomics, Cat. No. HR-MCMV-6).	Yunis et al., 2019

Supplementary references:

1. Allaire, P. D., Seyed Sadr, M., Chaineau, M., Seyed Sadr, E., Konefal S., Fotouhi M., et al. (2013). Interplay between Rab35 and Arf6 controls cargo recycling to coordinate cell adhesion and migration. J. Cell Sci. 126, 722-731. doi: 10.1242/jcs.112375.
2. An, H., Harper, J. W. (2018). Systematic analysis of ribophagy in human cells reveals bystander flux during selective autophagy. Nat. Cell Biol. 20, 135-143. doi: 10.1038/s41556-017-0007-x.
3. Bagh, M. B., Peng, S., Chandra, G., Zhang, Z., Singh, S. P., Pattabiraman, N., et al. (2017) Misrouting of v-ATPase subunit V0a1 dysregulates lysosomal acidification in a

neurodegenerative lysosomal storage disease model. Nat. Commun. 8:14612. doi: 10.1038/ncomms14612.

4. Barysch, S. V., Jahn, R., Rizzoli, S. O. (2010). A fluorescence-based in vitro assay for investigating early endosome dynamics. Nat. Protoc. 5, 1127-1137. doi:10.1038/nprot.2010.84.
5. Bavari, S., Bosio, C. M., Wiegand, E., Ruthel, G., Will, A. B., Geisbert, T. W., et al. (2002). Lipid raft microdomains: a gateway for compartmentalized trafficking of Ebola and Marburg viruses. J. Exp. Med. 195, 593-602. doi: 10.1084/jem.20011500.
6. Belaid, A., Ndiaye, P. D., Cerezo, M., Cailleteau, L., Brest, P., Klionsky, D. J., et al. (2014). Autophagy and SQSTM1 on the RHOA(d) again: emerging roles of autophagy in the degradation of signaling proteins. Autophagy 10, 201-208. doi: 10.4161/auto.27198.
7. Bobrie A., Colombo M., Krumeich S., Raposo G., Théry C. (2012). Diverse subpopulations of vesicles secreted by different intracellular mechanisms are present in exosome preparations obtained by differential ultracentrifugation. J. Extracell. Vesicles 1. doi: 10.3402/jev.v1i0.18397.
8. Bonuccelli G., Casimiro M. C., Sotgia F., Wang C., Liu M., Katiyar S., et al. (2009). Caveolin-1 (P132L), a common breast cancer mutation, confers mammary cell invasiveness and defines a novel stem cell/metastasis-associated gene signature. Am. J. Pathol. 174, 1650-1662. doi: 10.2353/ajpath.2009.080648.
9. Boonyaratanakornkit J., Schomacker H., Collins P., Schmidt A. (2013). Alix serves as an adaptor that allows human parainfluenza virus type 1 to interact with the host cell ESCRT system. PLoS One 8, e59462. doi: 10.1371/journal.pone.0059462.
10. Brankatschk B., Pons V., Parton R. G., Gruenberg J. (2011). Role of SNX16 in the dynamics of tubulo-cisternal membrane domains of late endosomes. PLoS One 6, e21771. doi: 10.1371/journal.pone.0021771.
11. Budt M., Niederstadt L., Valchanova R. S., Jonjić S., Brune W. (2009). Specific inhibition of the PKR-mediated antiviral response by the murine cytomegalovirus proteins m142 and m143. J. Virol. 83, 1260-1270. doi: 10.1128/JVI.01558-08.
12. Chevallier J., Chamoun Z., Jiang G., Prestwich G., Sakai N., Matile S., et al. (2008). Lysobisphosphatidic acid controls endosomal cholesterol levels. J. Biol. Chem. 283, 27871-2780. doi: 10.1074/jbc.M801463200.
13. Christoforidis S., McBride H. M., Burgoyne R. D., Zerial M. (1999). The Rab5 effector EEA1 is a core component of endosome docking. Nature 397, 621-625. doi: 10.1038/17618.
14. Cooper S., Sadok A., Bousgouni V., Bakal C. (2015). Apolar and polar transitions drive the conversion between amoeboid and mesenchymal shapes in melanoma cells. Mol. Biol. Cell 26, 4163-4170. doi: 10.1091/mbc.E15-06-0382.
15. Crawford D. C., Ramirez D. M., Trauterman B., Monteggia L. M., Kavalali E. T. (2017). Selective molecular impairment of spontaneous neurotransmission modulates synaptic efficacy. Nat. Commun. 8, 14436. doi: 10.1038/ncomms14436.
16. Delestre-Delacour C., Carmon O., Laguerre F., Estay-Ahumada C., Courel M., Elias S., et al. (2017). Myosin 1b and F-actin are involved in the control of secretory granule biogenesis. Sci. Rep. 7, 5172. doi:10.1038/s41598-017-05617-1.

17. Di Matteo P., Calvello M., Luin S., Marchetti L., Cattaneo A. (2017). An optimized procedure for the site-directed labeling of NGF and proNGF for imaging purposes. Front. Mol. Biosci. 4, 4. doi: 10.3389/fmolb.2017.00004.
18. Dikshit N., Bist P., Fenlon S. N., Pulloor N. K., Chua C. E., Scidmore, M. A., et al. (2015). Intracellular uropathogenic E. coli exploits host Rab35 for iron acquisition and survival within urinary bladder cells. PLoS Pathog. 11, e1005083. doi: 10.1371/journal.ppat.1005083.
19. Doerflinger, S. Y., Cortese, M., Romero-Brey, I., Menne, Z., Tubiana, T., Schenk, C., et al. (2017). Membrane alterations induced by nonstructural proteins of human norovirus. PLoS Pathog. 3, e1006705. doi: 10.1371/journal.ppat.1006705.
20. Eva, R., Crisp, S., Marland, J. R., Norman, J. C., Kanamarlapudi, V., French-Constant, C., et al. (2012). ARF6 directs axon transport and traffic of integrins and regulates axon growth in adult DRG neurons. J. Neurosci. 32, 10352-10364. doi:10.1523/JNEUROSCI.1409-12.2012.
21. Fernandez-Fernandez, M. R., Ruiz-Garcia, D., Martin-Solana, E., Chichon, F. J., Carrascosa, J. L., Fernandez, J. J. (2017). 3D electron tomography of brain tissue unveils distinct Golgi structures that sequester cytoplasmic contents in neurons. J. Cell Sci. 130, 83-89. doi: 10.1242/jcs.188060.
22. Garg S., Sharma M., Ung C., Tuli A., Barral D. C., Hava D. L., et al. (2011). Lysosomal trafficking, antigen presentation, and microbial killing are controlled by the Arf-like GTPase Arl8b. Immunity 35, 182-193. doi: 10.1016/j.immuni.2011.06.009.
23. Gundry, C., Marco, S., Rainero, E., Miller, B., Dornier, E., Mitchell, L., et al. (2017). Phosphorylation of Rab-coupling protein by LMTK3 controls Rab14-dependent EphA2 trafficking to promote cell: cell repulsion. Nat. Commun. 8, 14646. doi: 10.1038/ncomms14646.
24. Hiltbold, E. M., Poloso, N. J., Roche, P. A. (2003). MHC class II-peptide complexes and APC lipid rafts accumulate at the immunological synapse. J. Immunol. 170, 1329-1338. doi: 10.4049/jimmunol.170.3.1329.
25. Hiršl, L., Brizić, I., Jenuš, T., Juranić Lisnić, V., Reichel, J.J., Jurković, S., et al. (2018). Murine CMV expressing the high affinity NKG2D ligand MULT-1: A model for the development of cytomegalovirus-based vaccines. Front. Immunol. 9:991. doi: 10.3389/fimmu.2018.00991.
26. Homma, Y., Fukuda, M. (2016). Rabin8 regulates neurite outgrowth in both GEF activity-dependent and -independent manners. Mol. Biol. Cell 27, 2107-2118. doi: 10.1091/mbc.E16-02-0091.
27. Hong, N. H., Qi, A., Weaver, A. M. (2015). PI(3,5)P2 controls endosomal branched actin dynamics by regulating cortactin-actin interactions. J. Cell Biol. 210, 753-769. doi: 10.1083/jcb.201412127.
28. Hubert, V., Peschel, A., Langer, B., Gröger, M., Rees, A., Kain, R. (2016). LAMP-2 is required for incorporating syntaxin-17 into autophagosomes and for their fusion with lysosomes. Biol. Open. 5, 1516-1529. doi: 10.1242/bio.018648.
29. Humphreys, D., Davidson, A. C., Hume, P. J., Makin, L. E., Koronakis, V. (2013). Arf6 coordinates actin assembly through the WAVE complex, a mechanism usurped by Salmonella to invade host cells. Proc. Natl. Acad. Sci. USA 110, 16880-16885. doi:10.1073/pnas.1311680110.

30. Jung, Y. R., Lee, J. H., Sohn, K. C., Lee, Y., Seo, Y. J., Kim, C. D., et al. (2017). Adiponectin signaling regulates lipid production in human sebocytes. PLoS One.; 12, e0169824. doi: 10.1371/journal.pone.0169824.
31. Kahn, O. I., Schätzle, P., van de Willige, D., Tas, R. P., Lindhout, F. W., Portegies, S., et al. (2018). APC2 controls dendrite development by promoting microtubule dynamics. Nat. Commun. 9, 2773. doi:10.1038/s41467-018-05124-5.
32. Kawauchi, T., Nabeshima, Y. I. (2019). Growth arrest triggers extra-cell cycle regulatory function in neurons: possible involvement of p27(kip1) in membrane trafficking as well as cytoskeletal regulation. Front. Cell Dev. Biol. 7:64. doi:10.3389/fcell.2019.00064.
33. Kučić, N., Mahmutefendić, H., Lucin, P. (2005). Inhibition of protein kinases C prevents murine cytomegalovirus replication. J. Gen. Virol. 86, 2153-2161. doi: 10.1099/vir.0.80733-0.
34. Kutle, I., Sengstake, S., Templin, C., Glaß, M., Kubsch, T., Keyser, K. A., et al. (2017). The M25 gene products are critical for the cytopathic effect of mouse cytomegalovirus. Sci Rep. 7, 15588. doi: 10.1038/s41598-017-15783-x.
35. Lee, Y., Chung, S., Baek, I. K., Lee, T. H., Paik, S. Y., Lee, J. (2013). UNC119a bridges the transmission of Fyn signals to Rab11, leading to the completion of cytokinesis. Cell Cycle. 12, 1303-1315. doi: 10.4161/cc.24404.
36. Lenac Rovis, T., Kucan Brlic, P., Kaynan, N., Juranic Lisnic, V., Brizic I., Jordan, S., et al. (2016). Inflammatory monocytes and NK cells play a crucial role in DNAM-1-dependent control of cytomegalovirus infection. J. Exp. Med. 213, 1835-1850. doi: 10.1084/jem.20151899.
37. Liu, G., Zhang, F., Wang, R., London, L., London, S. D. (2014). Protective MCMV immunity by vaccination of the salivary gland via Wharton's duct: replication-deficient recombinant adenovirus expressing individual MCMV genes elicits protection similar to that of MCMV. FASEB J. 28, 1698-1710. doi: 10.1096/fj.13-244178.
38. Liu, X. D., Ko, S., Xu, Y., Fattah, E. A., Xiang, Q., Jagannath, C., et al. (2012). Transient aggregation of ubiquitinated proteins is a cytosolic unfolded protein response to inflammation and endoplasmic reticulum stress. J. Biol. Chem. 287, 19687-19698. doi: 10.1074/jbc.M112.350934.
39. MacDonald, E., Brown, L., Selvais, A., Liu, H., Waring, T., Newman, D., et al. (2018). HRS-WASH axis governs actin-mediated endosomal recycling and cell invasion. J. Cell Biol. 217, 2549-2564. doi: 10.1083/jcb.201710051.
40. Mahmutefendić, H., Blagojević, G., Ilić Tomaš, M., Kučić, N., Lučin, P. (2011). Segregation of open Major Histocompatibility Class I conformers at the plasma membrane and during endosomal trafficking reveals conformation-based sorting in the endosomal system. Int. J. Biochem. Cell Biol. 43, 504-515 doi: 10.1016/j.biocel.2010.12.002.
41. Meister, M., Zuk, A., Tikkanen, R. (2014). Role of dynamin and clathrin in the cellular trafficking of flotillins. FEBS J. 281, 2956-2976. doi: 10.1111/febs.12834.
42. Mendoza, P., Ortiz, R., Díaz, J., Quest, A.F., Leyton, L., Stupack, D., Torres, V.A. (2013). Rab5 activation promotes focal adhesion disassembly, migration and invasiveness in tumor cells. J. Cell Sci. 126, 3835-3847. doi: 10.1242/jcs.119727.

43. Monel, B., Compton, A. A., Bruel, T., Amraoui, S., Burlaud-Gaillard, J., Roy, N., et al. (2017). Zika virus induces massive cytoplasmic vacuolization and paraptosis-like death in infected cells. EMBO J. 36, 1653-1668. doi: 10.15252/embj.201695597.
44. Mu, F. T., Callaghan, J. M., Steele-Mortimer, O., Stenmark, H., Parton, R.G., Campbell, P. L., et al. (1995). EEA1, an early endosome-associated protein. EEA1 is a conserved alpha-helical peripheral membrane protein flanked by cysteine "fingers" and contains a calmodulin-binding IQ motif. J. Biol. Chem. 270, 13503-13511. doi: 10.15252/embj.201695597.
45. Munson, M. J., Allen, G. F., Toth, R., Campbell, D. G., Lucocq, J. M., Ganley, I. G. (2015). mTOR activates the VPS34-UVRAG complex to regulate autolysosomal tubulation and cell survival. EMBO J. 34, 2272-2290. doi: 10.15252/embj.201590992.
46. Nag, S., Rani, S., Mahanty, S., Bissig, C., Arora, P., Azevedo, C., et al. (2018). Rab4A organizes endosomal domains for sorting cargo to lysosome-related organelles. J. Cell Sci. 131, pii: jcs216226. doi: 10.1242/jcs.216226.
47. Nojima, H., Konishi, T., Freeman, C. M., Schuster, R. M., Japtok, L., Kleuser, B., et al. (2016) Chemokine receptors, CXCR1 and CXCR2, differentially regulate exosome release in hepatocytes. PLoS One 11, e0161443. doi: 10.1371/journal.pone.0161443.
48. Noun, G., Reboul, M., Abastado, J. P., Jaulin, C., Kourilsky, P., Pla, M. (1996). Alloreactive monoclonal antibodies select Kd molecules with different peptide profiles. J. Immunol. 157, 2455-2461. PMID: 8805645.
49. Ostenfeld, M. S., Jeppesen, D. K., Laurberg, J. R., Boysen, A. T., Bramsen, J. B., Primdal-Bengtson, B., et al. (2014). Cellular disposal of miR23b by RAB27-dependent exosome release is linked to acquisition of metastatic properties. Cancer Res. 74, 5758-5771. doi:10.1158/0008-5472.CAN-13-3512.
50. Park, Y. H., Seo, J. H., Park, J. H., Lee, H. S., Kim, K. W. (2017). Hsp70 acetylation prevents caspase dependent/independent apoptosis and autophagic cell death in cancer cells. Int. J. Oncol. 51, 573-578. doi: 10.3892/ijo.2017.4039.
51. Piragyte, I., Clapes, T., Polyzou, A., Klein Geltink, R.I., Lefkopoulos, S., Yin, N., et al. (2018). A metabolic interplay coordinated by HLX regulates myeloid differentiation and AML through partly overlapping pathways. Nat. Commun. 9, 3090. doi: 10.1038/s41467-018-05311-4.
52. Podlech, J., Pintea, R., Kropp, K. A., Fink, A., Lemmermann, N. A., Erlach, K. C., et al. (2010). Enhancerless cytomegalovirus is capable of establishing a low-level maintenance infection in severely immunodeficient host tissues but fails in exponential growth. J. Virol. 84, 6254-6261. doi: 10.1128/JVI.00419-10.
53. Qinwei, W., Xiaqin, S., Weihua, Y., Tianlan, L., Yanyan, R., Tianda, C., Dai, Z. (2016). RAB18, a protein associated with Warburg Micro syndrome, controls neuronal migration in the developing cerebral cortex. Mol. Brain 9, 19. doi: 10.1186/s13041-016-0198-2.
54. Razi, M., Chan, E. Y., Tooze, S. A. (2009). Early endosomes and endosomal coatomer are required for autophagy. J. Cell Biol. 185, 305-321. doi: 10.1083/jcb.200810098.
55. Rocca, D. L., Amici, M., Antoniou, A., Blanco Suarez, E., Halemani, N., Murk, K., et al. (2013). The small GTPase Arf1 modulates Arp2/3-mediated actin polymerization via PICK1 to regulate synaptic plasticity. Neuron 79, 293-307. doi: 10.1016/j.neuron.2013.05.003.
56. Sakagami, H., Sanda, M., Fukaya, M., Miyazaki, T., Sukegawa, J., Yanagisawa, T., et al. (2008). IQ ArfGEF/BRAG1 is a guanine nucleotide exchange factor for Arf6 that interacts

with PSD-95 at postsynaptic density of excitatory synapses. Neurosci. Res. 60, 199-212. doi: 10.1016/j.neures.2007.10.013.

57. Shaw, J. E., Epand, R. F., Epand, R. M., Li, Z., Bittman, R., Yip, C. M. (2006). Correlated fluorescence-atomic force microscopy of membrane domains: structure of fluorescence probes determines lipid localization. Biophys. J. 90, 2170-2178. doi: 10.1529/biophysj.105.073510.
58. Simone, L. C., Caplan, S., Naslavsky, N. (2013). Role of phosphatidylinositol 4,5-bisphosphate in regulating EHD2 plasma membrane localization. PLoS One 8, e74519. doi: 10.1371/journal.pone.0074519.
59. Simonsen, A., Lippé, R., Christoforidis, S., Gaullier, J. M., Brech, A., Callaghan, J., et al. (1998). EEA1 links PI(3)K function to Rab5 regulation of endosome fusion. Nature 394, 494-498. doi: 10.1038/28879.
60. Soffientini, U., Caridis, A. M., Dolan, S., Graham, A. (2014). Intracellular cholesterol transporters and modulation of hepatic lipid metabolism: Implications for diabetic dyslipidaemia and steatosis. Biochim. Biophys. Acta 1842, 1372-1382. doi: 10.1016/j.bbalip.2014.07.002.
61. Søreng, K., Munson, M. J., Lamb, C. A., Bjørndal, G. T., Pankiv, S., Carlsson, S. R., et al. (2018). SNX18 regulates ATG9A trafficking from recycling endosomes by recruiting Dynamin-2. EMBO Rep. 19, pii: e44837. doi:10.15252/embr.201744837.
62. Stevenson, N. L., White, I. J., McCormack, J. J., Robinson, C., Cutler, D. F., Nightingale, T. D. (2017). Clathrin-mediated post-fusion membrane retrieval influences the exocytic mode of endothelial Weibel-Palade bodies. J Cell Sci. 130, 2591-2605. doi: 10.1242/jcs.200840.
63. Takahashi, Y., Tanikawa, C., Miyamoto, T., Hirata, M., Wang, G., Ueda, K., et al. (2017). Regulation of tubular recycling endosome biogenesis by the p53-MICALL1 pathway. Int. J. Oncol. 51, 724-736. doi: 10.3892/ijo.2017.4060.
64. Treda, C., Popeda, M., Ksiazkiewicz, M., Grzela, D. P., Walczak, M. P., Banaszczyk, M., et al. (2016). EGFR activation leads to cell death independent of PI3K/AKT/mTOR in an AD293 cell line. PLoS One.; 11, e0155230. doi: 10.1371/journal.pone.0155230.
65. van der Kant, R., Zondervan, I., Janssen, L., Neefjes, J. (2013). Cholesterol-binding molecules MLN64 and ORP1L mark distinct late endosomes with transporters ABCA3 and NPC1. J. Lipid Res. 54, 2153–2165. doi: 10.1194/jlr.M037325.
66. Williams, K. C., McNeilly, R. E., Coppolino, M. G. (2014). SNAP23, Syntaxin4, and vesicle-associated membrane protein 7 (VAMP7) mediate trafficking of membrane type 1-matrix metalloproteinase (MT1-MMP) during invadopodium formation and tumor cell invasion. Mol. Biol. Cell 25, 2061-2070. doi: 10.1091/mbc.E13-10-0582.
67. Wong, M., Munro, S. (2014). Membrane trafficking. The specificity of vesicle traffic to the Golgi is encoded in the golgin coiled-coil proteins. Science 346, 1256898. doi: 10.1126/science.1256898.
68. Yunis, J., Farrell, H. E., Bruce, K., Lawler, C., Wyer, O., Davis-Poynter, N., et al. (2019). Murine cytomegalovirus glycoprotein O promotes epithelial cell infection in vivo. J. Virol. 93, pii: e01378-18. doi: 10.1128/JVI.01378-18.
69. Zhang, O., Wilson, M. C., Xu, W., Hsu, F. F., Turk, J., Kuhlmann, F. M., et al. (2009). Degradation of host sphingomyelin is essential for Leishmania virulence. PLoS Pathog. 5, e1000692. doi: 10.1371/journal.ppat.1000692.
