# Supplementary material for: Cytomegalovirus Generates Assembly Compartment in the Early Phase of Infection by Perturbation of Host-Cell Factors Recruitment at the Early Endosome/Endosomal Recycling Compartment/Trans-Golgi Interface
Source: Front Cell Dev Biol. 2020 Sep 11;8:563607. doi: 10.3389/fcell.2020.563607 (PMC7516400; doi:10.3389/fcell.2020.563607)
Supplement: Supplementary file 2 [file Data_Sheet_2.PDF]

Supplementary Material

Table S2. Reported subcellular localization and function of used markers (related to Fig. 1 and Fig. 9**)**

Marker	Subcellular localization and function
**Markers of the EE, ERC, TGN, and their activity at EE-ERC-TGN interface**	
**AP-2**	*Adaptor protein (AP)-2 complex is a type C marker that indicates ARF6- and PIP2-associated clathrin mobilization at membranes.* Localizes to the plasma membrane (PM). Facilitates clathrin-mediated cargo sorting. Binds to PI4,5P2 [phosphatidylinositol (4,5)-bisphosphate]. ARF (ADP-ribosylation factor) 6 (ARF6) contributes to the membrane recruitment of AP-2 (Park and Guo, 2014). Localizes at EEs in some cells (Sorkina et al., 1999). May regulate post-endocytic trafficking through the non-clathrin ARF6-dependent endocytic pathway (Lau and Chou, 2008).
**CD44**	*Type A marker which traffic through the peripheral endosomal recycling route (PM-pre-EE-PM) and displays a significant retention localization at the PM.* Enters the cell via ARF6-dependent and clathrin-independent endocytosis and cycles in the cortical area of the cell within the PM-pre-EE-PM route. A small fraction of internalized CD44 cycles through the EE-ERC-PM route with retention within the ERC (Dutta and Donaldson, 2015; Eyster et al., 2009; Mahmutefendić et al., 2017).
**MHC-I**	*Type A marker that traffic the endosomal recycling route (PM-pre-EE-EE-ERC-PM) and late endosomal (LE) degradative route (PM-pre-EE-EE-LE-Ly). Displays a significant retention localization in the ERC and LEs.* Fully conformed MHC-I molecules enter the cell via ARF6-dependent and clathrin-independent endocytosis. Cycle within the PM-pre-EE-EE-ERC-PM route with retention within the ERC and almost half is directed from EEs into LEs and degraded in lysosomes (Ly) (Eyster et al., 2009, Naslavsky et al., 2004, Mahmutefendić et al., 2011; Zagorac et al., 2012).
**Rae1**	*Type A marker that traffics the endosomal recycling route (PM-pre-EE-EE-ERC-PM). Displays a significant retention localization within the ERC.* Enter the cell via ARF6-dependent and clathrin-independent endocytosis and cycles within the PM-pre-EE-EE-ERC-PM route with retention within the ERC (Mahmutefendić et al., 2017).
**Dynamin 2**	*Type C marker that recruits to membranes for vesicle scission. Displays the site of activation of clathrin and WASH complex.* Required for the formation of clathrin-coated and non-clathrin-coated vesicles from the TGN (Cao et al., 2000). Associates with clathrin-coated buds at tubular EEs, ERC, and TGN. Mediates, at least in part, recycling of TfR from the ERC (van Dam and Stoorvogel, 2002; Jimah and Hinshaw, 2019). Regulates the fission of recycling tubules from EEs and thereby controls the maturation of EEs (Mesaki et al., 2011).
**APPL1**	*Type C marker that recruits to membranes of a subset of cortical pre-EEs. Displays the size of a subset of pre-EEs.* Defines a subset of precursor EEs, which are stable sorting stations - often called very early endosomes or pre-EEs. Enables rapid cargo recycling (Kalaidzidis et al., 2015). APPL1 localizes to a subset of

	Rab5-positive endosomes via interaction with Rab5 and compete with EEA1 for Rab5 binding (Zoncu et al., 2009; Diggins and Webb, 2017).
**Rab22a**	*Type B marker that recruits to membranes of EEs and a subset of Rab11-positive REs.*<br>Localizes at EEs and REs (Shakya et al., 2018). Recruits Rab5-GEF Rabex-5 to EEs and activates Rab5 (Rab22-Rabex-5-Rab5 cascade). Rabex-5 activates Rab5 as a GEF and inhibits Rab22 (Zhu et al., 2009). Promotes the assembly of a BLOC-1-BLOC-2-KIF13A complex on EE/SEs to generate REs. Present at long tubular structures of REs, which are either generated from Rab11A-positive vesicles or represent a subpopulation of Rab11-positive RE tubules (Shakya et al., 2018). Labels a fraction of total tubular RE intermediates (Rab22a-dependent REs), which are distinct from (i) MICAL-L1-syndapin2-dependent tubular REs (TREs) that originate from ARF6-positive endosomes, (ii) ERC generated from Golgi, and (iii) retromer-SNX27-dependent tubular endosomes (Bahl et al., 2016).
**Rab4a**	*Type C marker that displays the tubular recycling domain of EEs.*<br>Occupy distinct domain than Rab5 at EEs (Sönnichsen et al., 2000). Reported to be >90% (Marzesco et al., 1998) and almost 100% (Mari et al., 2001) membrane-associated. Activates on EEA1-positive EEs and generates a highly dynamic tubular domain. Activates Arl1, which recruits BIG1 and BIG2 and thereby activate ARF1 and ARF3 to promote assembly of AP-1 and AP-3 and GGA-3 (D'Souza et al., 2014). Constantly nucleates Rab4 domain at Rab5-positive endosomes and generates Rab4-positive tubules that mediate the exit of recycling cargo from EEs (Rink et al., 2005).
**Rab5a**	*Type C marker that displays the vacuolar domain of EEs.*<br>Rab5-positive endosomes are continuously generated/renewed in the cell periphery and move toward the cell center while growing in size (Rink et al., 2005). Reported to be ~50% in a membrane-associated form (Massignan et al., 2010; Liu and Grant, 2015). Targeted to membranes of EEs by Rabex-5/Rabaptin-5 complexes (Zhu et al., 2009). Activates generation of phosphatidylinositol (PtdIns)3P (PI3P) and generates PI3P-domain at early endosomes. Binds a large number of effector proteins, including EEA1 (required for fusion of endosomes) and Hrs/HGS (required for sorting of ubiquitinated cargo) (Frankel and Audhya, 2018). Excludes RAB-10 and thereby prevents the progression of membranes at the Golgi–endosomal interface, including the progression of EE membranes towards the ERC (Sasidharan et al., 2012).
**Rabenosyn-5**	*Type B marker that displays a subset of pre-EEs and intermediates that represent the exit from EEs towards the ERC or TGN.*<br>Recruited to PI3P endosomes (Nielsen et al., 2000). Defines a subset of pre-EEs that capture CD cargo (TfR) destined for recycling (Navaroli et al., 2011). Rab4 and Rab5 effector that interacts with EHD1 and EHD3. Controls EEs-to-Golgi transport (Naslavsky et al., 2009; Rai et al., 2019). Involved in the transport of cargo from EEs to ERC. Acts downstream of Rab5 and Rab4 and upstream of EHD1. Localizes at vesicular EEs (Naslavsky et al., 2004).
**EEA1**	*Type C marker that recruits to the vacuolar domain of EEs and indicates PI3P membrane composition.*<br>Rab5 effector protein present at the subdomain of EEs that acts as a tether for incoming vesicles during homo- and heterotypic fusion of EEs. Defines a subset of EEs (Simonsen et al., 1998; Wilson et al., 2000).
**Hrs/Hgs**	*Type B marker that recruits to a subset of EEA1-negative peripheral pre-EEs and vacuolar domain of EEs. Displays the PI3P domain of EEs and the site of MVB biogenesis.*<br>ESCRT-0 component that initiates the formation of multivesicular body (MVB) vesicles (McCullough et al., 2013). Localizes in a population of peripheral EEA1-negative endosomes distinct from APPL1-containing endosomes and in EEA1-positive endosomes (Flores-Rodriguez et al., 2015). Contains PI3P-binding FYVE domain and localizes at different region than EEA1 of EEs (Raiborg et al., 2001).

**PIKfyve**	*Type B marker that displays PI3P domain, which undergoes conversion to PI3,5P2 at EEs and LEs associated with recruitment of reverse budding machinery required for the development of intraluminal vesicles (ILVs).*<br>Binds to membrane PI3P and synthesizes PtdIns(3,5)P2 (PI3,5P2)and PtdIns5P (PI5P). Localizes to various degrees on EEs, MVBs, LEs, and most likely, the TGN, with variations depending on the cell type, the level of protein expression, and the rate of PI3P-to-PI3,5P2 conversion (Shisheva, 2008).
**Vps24**	*Type C marker that indicates activation of the reverse budding processes and development of ILVs. Indicates also the presence of PI3,5P2 at membranes.*<br>Localizes at EEs and LEs. Component of the ESCRT-III complex that participates in the final stages of reverse budding processes at MV endosomes, prior Vps4-mediated scission of intraluminal vesicles (Schöneberg et al., 2017). Cytosolic protein that binds to PI3,5P at endosomal membranes (Whitley et al., 2003).
**WASH 1**	*Type C marker that displays tubulation and actin recruitment at membranes of PI3P domain of EEs and REs.*<br>Component of the WASH complex. Present at EEs and the ERC (Cullen and Steinberg, 2018). Connects Retromer complex at PI3P endosomes and Retriever/CCC complex at LEs with filamentous actin and mediates sequence-dependent recycling of cargo to PM (Derivery et al., 2009; Cullen and Steinberg, 2018). Can recruit at EEs without Retromer - interacts with the CCC complex (Naslavsky and Caplan, 2018). Participate in EE/RE-to-TGN retrieval, EE/RE-to-PM recycling, and biogenesis of lysosomes (Wang et al., 2018).
**Vps35**	*Type C marker that displays recruitment of the retromer at EEs and ERC.*<br>Present at EE and the RE membranes. Component of the Retromer complex that controls recycling from endosomes to the TGN. Drives retrieval of endosomal cargo away from lysosomal degradation (Belenkaya et al., 2008; Matsudaira et al., 2015; McNally and Cullen, 2018).
**TfR**	*Type A marker that traffic the endosomal recycling route (PM-pre-EE-EE-ERC-PM). Displays a significant retention localization within the ERC.*<br>TfR localizes at the PM and after binding of the ligand (Tf) undergo rapid clathrin-dependent endocytosis, internalization into several subsets of pre-EEs, sorting into the tubular domain of EEs and recycling via the fast (Rab4-dependent) and slow (Rab11-dependent) recycling route (Grant and Donaldson 2009; Kalaizidis et al., 2015; Villaseñor et al., 2016 Mahmutefendić et al., 2018).
**Rab15**	*Type B marker that may recruit at endosomal intermediates within the EE-ERC interface.*<br>A small GTPase that colocalizes with Rab4, Rab5, and the TfR on SEs as well as Rab11 on the ERC (Zuk and Elferink, 2000). Regulates WPB exocytosis (Biesemann et al., 2017). Interacts with MICAL1, MICALCL, MICAL3, EHBP1, and EHBP1L1 (Pylypenko et al., 2018).
**ARF6**	*Type C marker that may recruit to PM and the ERC membranes. Displays pericentriolar subset of REs within the ERC. Indicates inactivation of Rab35.*<br>Localizes mainly to the PM and regulates the endocytic and recycling pathways and cytoskeletal remodeling (rev. by D'Souza-Schorey and Chavrier, 2006; Weekes et al., 2014). Reported to be membrane-associated to 90% in CHO cells (Gaschet and Hsu, 1999; Yang et al., 1998), 50% in J774 macrophages and B lymphocytes (Yang et al., 1998), and 70-80% in 3T3-L1 adipocytes (Yang et al., 1998). ARF6 may be attached to PM in the GDP form, and the site of attachment to PM is saturable (Klein et al., 2006). In neuron-like cells, ARF6/Rab8-REs are weak, hardly visible. After NGF stimulation, ARF6/Rab8-REs emerge beside centrosomes. Rabin8 activates Rab8 at ARF6-REs and Rab10 at Rab11-REs (Homma and Fukuda, 2016). A constitutively active mutant of ARF6 inhibits endocytic recycling (Grant and Donaldson, 2009; Kobayashi and Fukuda, 2012; Dutta and Donaldson, 2015). ARF6GTP recruits AP-2 (but not AP-1) onto membranes. In vivo, ARF6-GTP (but not ARF6-

	GDP) was found associated with AP-2 (Paleotti et al., 2005). As part of Rab35–ARF6 axis regulates exocytosis of WPB (Biesemann et al., 2017).
**BRAG2/ IQSEQ1/ ARFGEP100**	*Type C marker that indicates the activation of ARF6.* ARF-GEF that activates ARF6 in most of the cellular contexts (rev. by D'Souza and Casanova, 2016), including regulation of endocytosis (Dunphy et al., 2006). Endogenous BRAG2 also activates the class II ARFs (ARF4 and ARF5) (Moravec et al., 2012). BRAG2 binds clathrin and the AP-2 adaptor complex, and both BRAG2 and ARF5 localize to clathrin-coated pits at the plasma membrane (Moravec et al., 2012).
**Epi64/ TBC1D10**	*Type C marker that indicates activation of ARF6 and inactivation of Rab35 and Rab27a.* ARF6 effector and Rab35-GAP. Interacts with ARF6-GTP but not with ARF6-GDP (Hanono et al., 2006). Found to be ~80% membrane-bound in polarized cells (Imai et al., 2011). Indicates the presence of the active form of ARF6 and the inactivation of Rab35 (Chesneau et al., 2012). Detected at the PM. Limits Rab35 activation at the early steps of endocytosis (Patino-Lopez et al., 2008). Overexpression reduces Rab35-GTP level, induces intracellular vacuoles that are not Rab4, Rab5, and Rab11 positive, and reduces fast TfR recycling (Chesneau et al., 2012; Biesemann et al., 2017). Functions as a Rab27a GAP (Itoh and Fukuda, 2006).
**Rab35**	*Type C marker that displays a subset o REs within the ERC. Indicates ARF6 inactivation.* Localizes on the ERC (Kobayashi and Fukuda, 2012) and PM (Weekes et al., 2014). At PM, activated by an AP2/clathrin-binding GEF. Forms ARF6-Rab35-Rab8 regulatory cascade at the PM required for uncoating of CCVs (rev. by Klinkert and Echard, 2016). The activation pathway at ERC membranes unknown. Recruits its effector molecule MICAL-L1 to ARF6-positive recycling endosomes (Kobayashi and Fukuda, 2013). Promotes the generation of tubular recycling endosomes (TREs) by recruiting the effectors MICAL-L1 and ACAP2 to SE/REs (Allaire et al., 2013; Kobayashi et al., 2014) and fission of tubular endosomes by recruitment of EHD1 (Allaire et al. 2010). Via ACAP2 inactivates ARF6 (Chesneau et al., 2012; Biesemann et al., 2017). Correct sorting of CIE cargoes (such as CD44, CD98, and CD147) depends on Rab35 activation and ARF6 inactivation on SE/REs (Dutta and Donaldson, 2015). Recruits downstream Rabs (Rab8, Rab13, and Rab36), which promote recycling (Kobayashi et al., 2014).
**ACAP2**	*Type C marker that indicates activation of Rab35 and inactivation of ARF6 at REs.* Effector of Rab35 that act as ARF6-GAP and inactivates ARF6 at the cell periphery (Jackson et al., 2000) and the ERC (Kobayashi and Fukuda, 2012).
**EHD1**	*Type C marker that displays the sites of membrane scission at tubular membranes of EEs and the ERC.* Regulates endosomal transport at EEs and ERC. Found to be 80% membrane-bound (Guilherme et al., 2004). Localizes to tubular membranes that comprise part of the ERC (rev. by Naslavsky and Caplan, 2018). Facilitates tubulation and functions in the formation and scission of membrane carriers at REs (rev by Cullen and Steinberg, 2018; and Naslavsky and Caplan, 2018). Acts downstream of Rab10 (Grant and Caplan, 2008). Required for Rab5/Rabenosyn 5-dependent and Rab11/Rab11FIP2-dependent EE-to-ERC transport and Rab8a/MICAL-L1-dependent recycling at tubular recycling endosomes (TRE) (Sharma et al., 2009; Giridharan et al., 2013; Naslavsky and Caplan, 2018). Essential for the retrograde membrane transport of Shiga toxin (McKenzie et al., 2012) and Cholera toxin (Lee et al., 2015) from REs to the Golgi.
**MICAL-L1**	*Type C marker that indicates Rab35 activation on endosomes.* Rab35 effector that recruits to the ARF6-positive subset of ERC membranes (Kobayashi and Fukuda, 2013). Interacts with Rab35, Rab8A, Rab8B, Rab10, Rab13, Rab15, and Rab36 (Fukuda et al., 2008;

	Rahajeng et al., 2012). Recruits EHD1 and Rab8a to recycling tubules (Sharma et al., 2009; Giridharan et al., 2012).
**ACAP1**	*Type C marker that indicates ARF6 inactivation and clathrin-dependent recycling at the ERC.*<br>Recruited to membranes by Rab8A, Rab10, and Rab35. Acts as ARF6-GAP and indicates ARF6 inactivation (Jackson et al., 2000). Act as an adaptor for clathrin recruitment at REs and promotes recycling (Li et al., 2007).
**EHBP1**	*Type C marker that indicates Rab10-induced endosomal tubulation at REs.*<br>Effector protein, which in the complex with Rab10, generate endosome associated tubules (Wang et al., 2016; Eguchi et al., 2018).
**Rab10**	*Type B marker that may display EE-ERC endosomal intermediates and indicates tubulation within the ERC.*<br>Localizes at the PM (Weekes et al., 2014), EEs, ERC (Shi and Grant, 2013), and late Golgi/TGN compartments (Goud et al., 2018). In 3T3-L1 adipocytes, >95% in membrane-associated form and 80% in GTP-bound form (Sano et al., 2008). Acts at EE-ERC interface (Shi and Grant, 2013). In time-lapse series, Rab10 GFP traffics in rapidly moving vesicles between endosomes that are difficult to distinguish in static images (Babey et al., 2006). In *C. elegans* binds ARF6-GAP CNT1, a homolog of ACAP1 and ACAP2 (Shi et al., 2012). In *C. elegans*, Rab10 act downstream in the cascade of Rab5, which is required for the exit of cargo from EE to the ERC (Liu and Grant, 2015). Rab5 effector (LET-413/Erbin) recruits the RAB-10 GEF protein DENN-4 and activates Rab10, whereas Rab10 recruits Rab5-GAP TBC-2 and inactivates Rab5 (Liu et al., 2018). EHD1 acts downstream of Rab10 to mediate endosomal tubulation (Grant and Caplan, 2008).
**Rab14**	*Type B marker that may display EE-ERC and EE-TGN intermediates.*<br>Localizes predominantly at the EE-to-ERC pathway at an intermediate compartment of the TfR recycling pathway before Rab11 but after compartments positive for Rab4 and Rab5 (Linford et al., 2012). May define an independent and functionally distinct endocytic recycling pathway (Prekeris, 2012). Localizes also at the late Golgi/TGN compartments and participates in membrane trafficking between the Golgi and endosomes (Junutula et al., 2004; Proikas-Cezanne et al., 2006; Goud et al., 2018). Recruits RUFY1 to EEs, which sequentially recruits Rab4 and promotes the recycling of Tfn (Yamamoto et al., 2010).
**Rab11a**	*Type B marker that displays a subset of REs within the ERC and recycling carriers that tether to PM.*<br>Localizes at the ERC in the pericentriolar region (Sönnichsen et al., 2000) and peripheral recycling vesicles around the cellular tips where it regulates their tethering to PM (Takahashi et al., 2012). In BHK21 cells, almost the entire intracellular pool in a membrane-bound form (Chen et al., 1998); in N2a cells, 65% in a membrane-bound form (Massignan et al., 2010). Functions along the same sequence with ARFs (especially ARF1 and ARF3) at the ERC (Takahashi et al., 2012). Associates with late Golgi/TGN compartments (Goud et al., 2018).
**Rab8a**	*Type C marker that displays the subset of REs within the ERC and exocytic vesicles derived from the TGN. Indicates tubulation.*<br>Localizes at the ERC (Rahajeng et al., 2012; Vetter et al., 2015), PM (Weekes et al., 2016), late TGN, exocytic vesicles (Grigoriev et al., 2011) and stressed lysosomes (Eguchi et al., 2018). In BHK21 cells, almost the entire intracellular pool in a membrane-bound form (Chen et al., 1998). It can be activated at Rab11-positive membranes by recruitment of Rabin8 (Rab8-GEF) to Rab11-GTP and mediate the conversion of Rab11-positive membranes to Rab8-positive membranes (Vetter et al., 2015). Rabin8 also recruits Rab8 at ARF6-REs (Homma and Fukuda, 2016). ARF6 regulates Rab8a, and both stimulate the formation of recycling tubules and cell protrusions (Kobayashi and Fukuda, 2012). At late Golgi/TGN compartments, Rab6a mediates recruitment of Rab8a to exocytic vesicles, and Rab8a is required for their fission from the Golgi (Grigoriev et al., 2011; Goud et al., 2018).

	Mediates Golgi-to-RE transport in polarized MDCK cells (Babbey et al., 2006). Localizes to tubular membranes (Peränen et al., 2011). MICAL-L1 recruits Rab8a to tubular endosomes (Sharma et al., 2009; Giridharan et al., 2012; Kobayashi et al., 2014).
**Rab13**	*Type B marker that may recruit at a subset of REs within the ERC and display ERC-TGN intermediates.* Localizes on REs (Sakane et al., 2010; Kobayashi and Fukuda, 2012; Kobayashi et al., 2014) and late Golgi/TGN compartments (Goud et al., 2018). Reported to be in >90% in a membrane-associated form (Marzesco et al., 1998). Functions at the interface between TGN and ERC, at an earlier membrane trafficking step distinct from Rab8 or Rab10 (Nokes et al., 2008). Traffics on vesicles derived from REs and LEs in both its inactive and active form (Ioannou et al., 2016).
**Rab36**	*Type B marker that may recruit at a subset of REs within the ERC and display ERC-TGN intermediates.* Localizes on REs (Kobayashi et al., 2014) and the Golgi (Goud et al., 2018). Recruited as downstream Rab by Rab35 and MICLAL-L1 (Kobayashi et al., 2014). RUTBC2 effector of Rab9a is a GAP for Rab36 at endosomes and TGN (Nottingham et al., 2012).
**Evectin-2/ PLEKHB2**	*Type C marker that recruits to tubular structures within the ERC which participate in the retrograde transport. Indicates enrichment of phosphatidylserine (PS) at the cytoplasmic leaflet of ERC membranes.* Localizes at tubulovesicular structures within the ERC (Uchida et al., 2011). Binds to phosphatidylserine (PS) and displays and PS-dependent recycling endosomes (Matsudaira et al., 2017). Indicates retrograde transport route - required for retrograde transport from REs to TGN but not for recycling from the ERC to the PM (Taguchi 2013). Recruits EHD1, which facilitates tubulation and fission (Lee et al., 2015; Matsudaira et al., 2017). Binds SMAP2, GAP for ARF1, at REs, which mobilizes AP1 and clathrin for retrograde transport of CTxB (Matsudaira et al., 2015).
**Rab31/ Rab22b**	*Type B marker that may display TGN compartments and peripheral TGN-derived compartment/pre-EEs.* Associates with late Golgi/TGN compartments (Ng et al., 2007; Goud et al., 2018) on tubulo-vesicular carriers that bud from TGN, travel along microtubule and fuse with EEs at the cell periphery (Rodriguez-Gabin et al., 2009). Interacts with APPL2 – pre-EEs (King et al., 2012).
**ARF3**	*Type C marker that displays sites of a generation of recycling carriers at REs.* Localizes at the PM (Weekes et al., 2014), TGN (Manolea et al., 2010), and REs (Kondo et al., 2012). In combination with ARF1 required for the integrity of the ERC (Kondo et al., 2012) and recycling from the ERC (Volpicelli-Daley et al., 2005). It can be activated at Rab4/Arl1-initiated TEN of EEs, where it can recruit AP1, AP2, and GGAs (D'Souza et al., 2014).
**BIG1**	*Type C marker that displays the site of ARF activation at REs and the TGN.* Localize to TGN, recruit AP1, and activate ARF1 and ARF3 (Zhao et al., 2002). ARF-like protein Arl1 recruits BIG1 to TGN (Christis and Munro, 2012) and on the Rab4-enriched tubular subdomain of EEs, which activate ARF1 and ARF3 to promote assembly of AP-1 and AP-3 (D'Souza et al., 2014).
**BIG2**	*Type C marker that displays the site of ARF activation and at the TGN and REs.* Present at the TGN (Zhao et al., 2002) and on perinuclear Rab11-positive REs (Shin et al., 2004; Shen et al., 2006; Ishizaki et al., 2008). Activates ARF1 and ARF3 at REs and TGN (Boal and Stephens, 2010). ARF-like protein Arl1 recruit BIG1 to TGN (Christis and Munro, 2012) and Rab4-enriched tubular subdomain of EEs (D'Souza et al., 2014).

**TGN38**	*Type A marker that traffic TGN-PM-EE-RE-TGN route. Displays a major retention localization within the TGN.*<br>Integral membrane protein predominantly localized in the TGN. Travels TGN-PM-EE-RE-TGN route and delivered to TGN via REs (Mallet and Maxfield, 1999).
**Golgin 97**	*Type C marker that resides on TGN membranes.*<br>Trans-Golgi localized member of the Golgin family. Attached to the membrane via Arl1. Acts as a tether that capture transport vesicles derived from SEs and possibly other membranes (rev. by Witkos and Lowe, 2016).
**STX6**	*Type B marker that displays TGN membranes. It may display EE and ERC membranes.*<br>Cholesterol-binding protein (Reverter et al., 2014) that predominantly localizes at the TGN (Bock et al., 1997), but also has been identified in EEs (Simonsen et al., 1999) and REs (Schindler et al., 2015). After cholesterol depletion in TGN translocates to Rab11-containing REs (Reverter et al., 2014). t-SNARE – forms the complex with VAMP4 (v-SNARE) for retrograde transport from endosomes to TGN (Perez-Victoria et al., 2009).
**Rab6a**	*Type C marker that displays TGN membranes.*<br>Highly enriched in trans-Golgi cisternae of the TGN (Liu and Storrie, 2015) and exocytic vesicles (Grigoriev et al., 2011). Reported to be in >90% in a membrane-associated form (Marzesco et al., 1998). Cooperates with Rab8a in exocytic vesicle generation and trafficking (Grigoriev et al., 2011).
**Furin**	*Type A marker that traffics the TGN-PM-EE-LE-TGN route. Displays significant retention localization at clathrin-coated regions of the TGN.*<br>Transmembrane endoprotease mainly localized at clathrin-coated regions of the TGN (rev. by Thomas, 2002). Buds from TGN by the binding to AP1 and traffic to EEs. Transported to the PM and internalized by CDE via AP2/clathrin/dynamin. In EEs, redirected to LEs and the TGN (rev. by Thomas, 2002; Chia et al., 2011).
**M6PR**	*Type A marker that traffics TGN-PM-EE-ERC-TGN and EE-LE-TGN route.*<br>Cation-independent mannose 6-phosphate receptor (M6PR) is found at TGN, PM, EEs, REs, and LEs (rev. by Ghosh et al., 2003). After endocytosis, M6PRs enter EE/SEs and accumulates in the ERC, whereby a significant fraction is recycled to PM, and some are delivered to the TGN and/or LEs (Lin et al., 2004).
**Vti1a**	*Type B marker that displays entry sites of EE/RE derived carriers to the TGN.*<br>Found predominantly on the Golgi and TGN (Kreykenbohm et al., 2002). Interacts with STX6 and ST16 to form TGN-localized t-SNARE complex involved in the retrograde transport between EEs/REs and TGN (Mallard et al., 2002). May also form the t-SNARE complex STX5 and STX16 at the cis- and medial-Golgi membranes (Mallard et al., 2002).
**AP-1**	*Type C marker that displays sites of cargo sorting and membrane budding at TGN and RE membranes.*<br>Clathrin adaptor protein complex 1 (AP1) mainly recruited to TGN (rev. by Park and Guo, 2014) and RE membranes (Matsudaira et al., 2015). May also be recruited at EEs, mediating cargo sorting for recycling or retrograde transport to TGN as multimeric Retromer complex and GGA adaptors (Bonifacino and Hurley, 2008). AP1A is recruited by class I ARFs to TGN (D'Souza -Schorey and Chavrier 2006), whereas AP1B acts mainly at the RE by interacting with ARF6 (Shteyn et al., 2011), mediating recycling to PM and ERC-to-TGN route (Hsu and Prekeris, 2010). Found to be 95% membrane-bound in CHO cells (Gaschet and Hsu, 1999).
**ARF1**	*Type C marker that displays sites of vesicle budding and clathrin recruitment at REs and TGN.*

	Class I ARF that can be activated at the PM, where it regulates dynamin-independent endocytosis (Kumari and Mayor, 2008), EEs (D'Souza et al., 2014), REs (Volpicelli-Daley et al., 2005), the TGN (D'Souza -Schorey and Chavrier 2006), and the early Golgi (Honda et a., 2005). Recruits AP1/AP3 and GGAs to the TGN (D'Souza -Schorey and Chavrier, 2006). It can be activated at Rab4/Arl1-initiated TEN of EEs, where it can recruit AP1, AP2, and GGAs (D'Souza et al., 2014). Required for the retrograde transport of CTxB from REs to the Golgi (Matsudaira et al., 2015) and together with ARF4 control retrograde transport of TGN38 and M6PR from REs to TGN (Nakai et al., 2013).
**ARF4**	*Type C marker that displays sites of vesicle budding and clathrin recruitment at REs and TGN.*<br>Class II ARF that can be activated at the TGN (Mazelova et al., 2009), REs (Volpicelli-Daley et al., 2005; Nakai et al., 2013) and ER-Golgi intermediate compartment (Chun et al., 2008). ARF1 and ARF4 control retrograde transport of TGN38 and M6PR from REs to TGN (Nakai et al., 2013). ARF4+5 activates at distinct sites of REs than ARF1+3 (Volpicelli-Daley et al., 2005).
**ARF5**	*Type C marker that displays sites of vesicle budding and clathrin recruitment at REs and TGN.*<br>Class II ARF that can be activated at the PM (Moravec et al., 2012), REs (Volpicelli-Daley et al., 2005), TGN (Lowery et al., 2013) and ER-Golgi intermediate compartment (Chun et al., 2008). ARF4 and ARF5 activated by GBF1 promote recruitment of BIG1 and BIG2 at the TGN after the recruitment of AP-1 (Lowery et al., 2013).
**Golgi markers**	
**Rab41/ Rab6d**	*Type B marker that displays pre-Golgi linker compartments.*<br>Member of Rab VI subfamily that has an active role in maintenance of the Golgi architecture (Liu et al., 2013; Liu and Storrie, 2015) and may regulate pre-Golgi trafficking steps and linker compartments (Goud et al., 2018).
**GS15**	*Type B marker that displays transport between medial- and trans-Golgi.*<br>A v-SNARE that complexes with t-SNAREs syntaxin-5, GS28, and Ykt6 (Xu et al., 2002). Mainly found in the medial-Golgi and adjacent tubulo-vesicular elements. Redistributes from the Golgi to the endosomes when the recycling endosome is perturbed, suggesting that GS15 may cycle between endosomes and the Golgi (Tai et al., 2004).
**GM130**	*Type A marker that resides at membranes of the cis-Golgi.*<br>Cis-Golgi localized member of the Golgin family. Attached to the membrane via GRASP65 (rev by Witkos and Lowe, 2016). Cycles via membranous tubules between the cis-Golgi and late IC stations (Marra et al., 2001).
**Rab9a**	*Type B marker that displays intermediates in the transport between TGN and EEs, and membrane domains of LEs distinct from Rab7-positive domains.*<br>Localizes at LEs and the Golgi. Reported to be ~95% in a membrane-bound form (Seaman et al., 2009). At LEs, defines distinct domain than Rab7 (Barbero et al., 2002). Detected at Rab5-EEs before Rab/Rab7a conversion (Gillingham et al., 2014). Acts downstream of Rab5a and inactivates Rab5a by recruiting Rab5 GAP SGSM3 (Gillingham et al., 2014). It can be activated at TGN and mediate transport to EEs and either transport or conversion from EEs to LEs (Kucera et al., 2016). Recruits Rab36 GAP RUTBC2 and inactivate Rab36 in the endosomal system (Nottingham et al., 2012).
**Late endosomal markers**	
**CD63**	*Type A marker that displays luminal and limiting membranes of LEs and LE-derived organelles (LRO) and transport carriers.*

	Integral membrane protein present at luminal and limiting membranes of LEs and LRO (Lebrand et al., 2002) and exosomes (Pols et al., 2009). May be present at the PM (Weekes et al., 2014).
**Lamp1**	*Type A marker that displays a subset of LE limiting membranes and LE-derived organelles (LRO) and transport carriers.*<br>Mainly present on LEs and lysosomes, and at low levels on the PM and EEs (Cook et al., 2004; Janvier and Bonifacino, 2005). Reaches the PM through LRO secretion in a process that requires Rab27a and Gadkin, which recruits AP1 and AP2 (Laulagnier et al., 2011). Present at the early subset of Rab7-positive and Rab7-negative LE membranes and on Rab7-positive subset of late LE membranes (Humphries et al., 2011). Present at limiting membranes of LEs. Traffic to lysosomes via the direct route (TGN-EE-LE-Ly) or indirect route (TGN-PM-EE-LE-Ly) (Cook et al., 2004; Lebrand et al., 2002; Janvier and Bonifacino, 2005).
**NPC1**	*Type A marker that displays a subset of LE Lamp1-negative limiting membranes.*<br>Present mostly on a subset of Lamp1-negative and Rab7-positive LE membranes (Garver et al., 2000). Almost exclusively localized to ORP1L positive vesicles while being absent from MLN64-positive late endosomes (van der Kant et al., 2013).
**Rab7a**	*Type B marker that displays a subset of LE limiting membranes.*<br>Small GTPase that controls transport towards late endosomes and lysosomes (Bucci et al., 2000). Localize at both EEs and LEs. Reported to be ~95% in a membrane-bound form (Seaman et al., 2009). Dispensable for the transfer of cargo from the LE/MVB to the lysosome and endocytic organelle maintenance (Vanlandingham and Ceresa, 2009). Defines a distinct domain of LEs than Rab9 (Barbero et al., 2002). Recruits retromer on endosomes (Rojas et al., 2008).
**GM1**	*Type A marker that displays luminal membranes of LEs and PM.*<br>Ganglioside (glycosphingolipid) that can be found at the PM and luminal membranes of LEs (Möbius et al., 1999).
**Rab27a**	*Type B marker that displays LRO and LE-derived transport carriers.*<br>A small GTPase that regulates secretory pathway of non-secretory cells (Fukuda, 2013). Regulates distal steps in the exocytosis of multivesicular endosomes (Fukuda, 2013). Involved in LRO organelle biogenesis (Raposo et al., 2007; Laulagnier et al., 2011; Biesemann et al., 2017) and exosome secretion (Fukuda, 2013).
**Rab27b**	*Type B marker that displays a subset of LE limiting membranes and LE-derived transport carriers.*<br>A small GTPase that regulates secretory pathway of non-secretory cells (Fukuda, 2013). Regulates the proximal step in the exocytosis of multivesicular endosomes (Fukuda, 2013).
**ER marker**	
**Rab18**	*Type B marker that displays ER and pre-Golgi intermediates.*<br>A small GTPase present at the PM fractions (Weekes et al., 2014) and the ER (Gerondopoulos et al., 2014). Reported to be ~67% cytosolic and 33% in a membrane-associated form (Overmeyer et al., 2001). Required for the maintenance of normal ER morphology, including perinuclear ER sheets and peripheral tubular ER networks (Gerondopoulos et al., 2014). May also regulates pre-Golgi membranous organelle trafficking (Goud et al., 2018).
**Marker of mitochondria**	
**AIFM1**	*Type A marker that displays inner membranes of mitochondria.*<br>Apoptosis-inducing factor 1, mitochondrial protein inserted into the inner mitochondrial membrane (Norberg et al., 2010).

Autophagy pathway markers	
**LC3B**	*Type C marker that displays subset autophagosomal membranes.*<br>The best-studied endogenous marker of autophagy. After the autophagic response, LC3B-II accumulates at membranes and can be used as an indicator of autophagic flux. Displays autophagosomes. In untreated cells, LC3B is evenly distributed in the cytoplasm and does not localize in the nucleus. (Kuma et al., 2007; Koukourakis et al., 2015).
**p62**	*Type C marker that displays sites of autophagic degradation.*<br>Localizes to the autophagosome formation site on the ER (Itakura and Mizushima, 2011).

Supplementary references:

1. Allaire, P. D., Marat, A. L., Dall'Armi, C., Di Paolo, G., McPherson, P. S., Ritter, B. (2010). The Connecdenn DENN domain: a GEF for Rab35 mediating cargo-specific exit from early endosomes. Mol. Cell 37, 370–382. doi: 10.1016/j.molcel.2009.12.037.
2. Allaire, P. D., Seyed Sadr, M., Chaineau, M., Seyed Sadr, E., Konefal, S., Fotouhi, M., *et al.* (2013). Interplay between Rab35 and ARF6 controls cargo recycling to coordinate cell adhesion and migration. J. Cell Sci. 126, 722-731. doi: 10.1242/jcs.112375.
3. Babbey, C. M., Ahktar, N., Wang, E., Chen, C. C., Grant, B. D., Dunn, K. W. (2006). Rab10 regulates membrane transport through early endosomes of polarized MDCK cells. Mol. Biol. Cell 17, 3156–3175. doi: 10.1091/mbc.e05-08-0799.
4. Bahl, K., Xie, S., Spagnol, G., Sorgen, P., Naslavsky, N., Caplan, S. (2016). EHD3 protein is required for tubular recycling endosome stabilization, and an asparagine-glutamic acid residue pair within its Eps15 homology (EH) domain dictates its selective binding to NPF peptides. J. Biol. Chem. 291, 13465-13478. doi: 10.1074/jbc.M116.716407.
5. Barbero, P., Bittova, L., Pfeffer, S. R. (2002). Visualization of Rab9-medicated vesicle transport from endosomes to the trans-Golgi in living cells. J. Cell Biol. 156, 511–518. doi: 10.1083/jcb.200109030.
6. Belenkaya, T. Y., Wu, Y., Tang, X., Zhou, B., Cheng, L., Sharma, Y.V., *et al.* (2008). The retromer complex influences Wnt secretion by recycling wntless from endosomes to the trans-Golgi network. Dev. Cell 14, 120-131. doi: 10.1016/j.devcel.2007.12.003.
7. Biesemann, A., Gorontzi, A., Barr, F., Gerke, V. (2017). Rab35 protein regulates evoked exocytosis of endothelial Weibel-Palade bodies. J. Biol. Chem 292, 11631-11640. doi: 10.1074/jbc.M116.773333.
8. Boal, F., Stephens, D. J. (2010). Specific functions of BIG1 and BIG2 in endomembrane organization. PLoS One 5, e9898. doi:10.1371/journal.pone.0009898.
9. Bock, J. B., Klumperman, J., Davanger, S., Scheller, R. H. (1997). Syntaxin 6 functions in trans-Golgi network vesicle trafficking. Mol. Biol. Cell 8, 1261-1271. doi: 10.1091/mbc.8.7.1261.
10. Bonifacino, J. S., Hurley, J. H. (2008). Retromer. Curr. Opin. Cell Biol. 20, 427-436. doi: 10.1016/j.ceb.2008.03.009.
11. Bucci, C., Thomsen, P., Nicoziani, P., McCarthy, J., van Deurs, B. (2000). Rab7: a key to lysosome biogenesis. Mol. Biol. Cell 11, 467-480. doi: 10.1091/mbc.11.2.467.
12. Cao, H., Thompson, H. M., Krueger, E.W., McNiven, M. A. (2000). Disruption of Golgi structure and function in mammalian cells expressing a mutant dynamin. J. Cell Sci. 113, 1993-2002. doi: 10.3389/fendo.2013.00126.
13. Chen, W., Feng, Y., Chen, D., Wandinger-Ness, A. (1998). Rab11 is required for trans-Golgi network-to-plasma membrane transport and a preferential target for GDP dissociation inhibitor. Mol. Biol. Cell 9, 3241-3257. doi: 10.1091/mbc.9.11.3241.
14. Chesneau, L., Dambournet, D., Machicoane, M., Kouranti, I., Fukuda, M., Goud, B. (2012). An ARF6/Rab35 GTPase cascade for endocytic recycling and successful cytokinesis. Curr. Biol. 22, 147-153. doi: 10.1016/j.cub.2011.11.058.
15. Chia, P. Z., Gasnereau, I., Lieu, Z.Z., Gleeson, P. A. (2011). Rab9-dependent retrograde transport and endosomal sorting of the endopeptidase furin. J. Cell Sci. 124, 2401-2413. doi: 10.1242/jcs.083782.

16. Christis, C., Munro, S. (2012). The small G protein Arl1 directs the trans-Golgi-specific targeting of the ARF1 exchange factors BIG1 and BIG2. J. Cell Biol. 196, 327–335. doi: 10.1083/jcb.201107115.

17. Chun, J., Shapovalova, Z., Dejgaard, S.Y., Presley, J. F., Melançon, P. (2008). Characterization of class I and II ADP-ribosylation factors (ARFs) in live cells: GDP-bound class II ARFs associate with the ER-Golgi intermediate compartment independently of GBF1. Mol. Biol. Cell 19, 3488-3500. doi: 10.1091/mbc.e08-04-0373.

18. Cook, N. R., Row, P. E, Davidson, H. W. (2004). Lysosome-associated membrane protein 1 (Lamp1) traffics directly from the TGN to early endosomes. Traffic 5, 685-699. doi: 10.1111/j.1600-0854.2004.00212.

19. Cullen, P. J., Steinberg, F. (2018). To degrade or not to degrade: mechanisms and significance of endocytic recycling. Nat. Rev. Mol. Cell Biol. 19, 679-696. doi: 10.1038/s41580-018-0053-7.

20. D'Souza -Schorey, C., Chavrier, P. (2006). ARF proteins: roles in membrane traffic and beyond. Nat. Rev. Mol. Cell Biol. 7, 347–358. doi: 10.1038/nrm1910.

21. De Matteis, M. A., Corda, D., Luini, A. (2009). The Golgi complex. FEBS Lett. 583, 3731. doi: 10.1016/j.febslet.2009.10.082.

22. Derivery, E., Sousa, C., Gautier, J. J., Lombard, B., Loew, D., Gautreau, A. (2009). The Arp2/3 activator WASH controls the fission of endosomes through a large multiprotein complex. Dev. Cell 17, 712-723. doi: 10.1016/j.devcel.2009.09.010.

23. Diggins, N. L., Webb, D. J. (2017). APPL1 is a multifunctional endosomal signaling adaptor protein. Biochem. Soc. Trans. 45, 771-779. doi: 10.1042/BST20160191.

24. D'Souza, R. S., Casanova, J. E. (2016). The BRAG/IQSec family of ARF GEFs. Small GTPases 7, 257–264. doi: 10.1080/21541248.2016.1219442.

25. D'Souza, R. S., Semus, R., Billings, E. A., Meyer, C. B., Conger, K., Casanova, J. E. (2014). Rab4 orchestrates a small GTPase cascade for recruitment of adaptor proteins to early endosomes. Curr. Biol. 24, 1187-1198. doi: 10.1016/j.cub.2014.04.003.

26. Dunphy, J. L., Moravec, R., Ly, K., Lasell, T. K., Melancon, P., Casanova, J. E. (2006). The ARF6 GEF GEP100/BRAG2 regulates cell adhesion by controlling endocytosis of beta1 integrins. Curr. Biol. 16, 315-320. doi: 10.1016/j.cub.2005.12.032.

27. Dutta, D., Donaldson, J. G. (2015). Rab and ARF G proteins in endosomal trafficking. Methods Cell Biol. 130, 127-138. doi: 10.1016/bs.mcb.2015.04.004.

28. Dutta, D., Donaldson, J. G. (2015). Sorting of clathrin-independent cargo proteins depends on Rab35 delivered by clathrin-mediated endocytosis. Traffic 16, 994-1009. doi: 10.1111/tra.12302.

29. Eguchi, T., Kuwahara, T., Sakurai, M., Komori, T., Fujimoto, T., Ito, G., *et al.* (2018). LRRK2 and its substrate Rab GTPases are sequentially targeted onto stressed lysosomes and maintain their homeostasis. Proc. Natl. Acad. Sci. USA 115, E9115-E9124. doi: 10.1073/pnas.1812196115.

30. Eyster, C. A., Higginson, J. D., Huebner, R., Porat-Shliom, N., Weigert, R., Wu, W. W., *et al.* (2009). Discovery of new cargo proteins that enter cells through clathrin-independent endocytosis. Traffic 10, 590-599. doi: 10.1111/j.1600-0854.2009.00894.x.

31. Flores-Rodriguez, N., Kenwright, D. A., Chung, P. H., Harrison, A. W., Stefani, F., Waigh, T. A., *et al.* (2015). ESCRT-0 marks an APPL1-independent transit route for EGFR between the cell surface and the EEA1-positive early endosome. J. Cell Sci. 128, 755-767. doi: 10.1242/jcs.161786.

32. Frankel, E. B., Audhya A. (2018). ESCRT-dependent cargo sorting at multivesicular endosomes. Semin. Cell Dev. Biol. 74, 4-10. doi:10.1016/j.semcdb.2017.08.020.

33. Fukuda, M. (2013). Rab27 effectors, pleiotropic regulators in secretory pathways. Traffic 14, 949-963. doi: 10.1111/tra.12083.

34. Fukuda, M., Kanno, E., Ishibashi, K., Itoh, T. (2008). Large scale screening for novel Rab effectors reveals unexpected broad Rab binding specificity. Mol. Cell Proteomics 7, 1031-1042. doi: 10.1074/mcp.M700569-MCP200.

35. Garver, W. S., Heidenreich, R. A., Erickson, R. P., Thomas, M. A., Wilson, J. M. (2000). Localization of the murine Niemann-Pick C1 protein to two distinct intracellular compartments. J. Lipid Res. 41, 673-687. 10787428.

36. Gaschet, J., Hsu V. W. (1999). Distribution of ARF6 between membrane and cytosol is regulated by its GTPase cycle. J. Biol. Chem. 274, 20040-20045. doi: 10.1074/jbc.274.28.20040.

37. Gerondopoulos, A., Bastos, R. N., Yoshimura, S., Anderson, R., Carpanini, S., Aligianis, I., *et al.* (2014). Rab18 and a Rab18 GEF complex are required for normal ER structure. J. Cell Biol. 205, 707-720. doi: 10.1083/jcb.201403026.

38. Ghosh, P., Dahms, N. M., Kornfeld, S. (2003). Mannose 6-phosphate receptors: new twists in the tale. Nat. Rev. Mol. Cell Biol. 4, 202-212. doi: 10.1038/nrm1050 12612639.

39. Gillingham, A. K., Sinka, R., Torres, I. L., Lilley, K. S., Munro, S. (2014). Toward a comprehensive map of the effectors of Rab GTPases. Dev. Cell 31, 358-373. doi:10.1016/j.devcel.2014.10.007.

40. Giridharan, S. S., Cai, B., Naslavsky, N., Caplan, S. (2012). Trafficking cascades mediated by Rab35 and its membrane hub effector, MICAL-L1. Commun. Integr. Biol. 5, 384-387. doi: 10.4161/cib.20064.

41. Goud, B., Liu, S., Storrie, B. (2018). Rab proteins as major determinants of the Golgi complex structure. Small GTPases 9, 66-75. doi: 10.1080/21541248.2017.1384087.

42. Grant, B. D., Caplan, S. (2008). Mechanisms of EHD/RME-1 protein function in endocytic transport, Traffic 9, 2043-2052. doi: 10.1111/j.1600-0854.2008.00834.x.

43. Grant, B. D., Donaldson, J. G. (2009). Pathways and mechanisms of endocytic recycling. Nat. Rev. Mol. Cell Biol. 10, 597–608. doi: 10.1038/nrm2755.

44. Grigoriev, I., Yu, K. L., Martinez-Sanchez, E., Serra-Marques, A., Smal, I., Meijering, E., *et al.* (2011). Rab6, Rab8, and MICAL3 cooperate in controlling docking and fusion of exocytotic carriers. Curr. Biol. 21, 967-974. doi: 10.1016/j.cub.2011.04.030.

45. Guilherme, A., Soriano, N. A., Furcinitti, P. S., Czech, M. P. (2004). Role of EHD1 and EHBP1 in perinuclear sorting and insulin-regulated GLUT4 recycling in 3T3-L1 adipocytes. J. Biol. Chem. 279, 40062-40075. doi: 10.1074/jbc.M401918200.

46. Hanono, A., Garbett, D., Reczek, D., Chambers, D. N., Bretscher, A. (2006). EPI64 regulates microvillar subdomains and structure. J. Cell Biol.175, 803-813. doi: 10.1083/jcb.200604046.

47. Holthuis, J. C., Nichols, B. J., Dhruvakumar, S., Pelham, H. R. (1998). Two syntaxin homologues in the TGN/endosomal system of yeast. EMBO J. 17, 113-126. doi: 10.1093/emboj/17.1.113.

48. Homma, Y., Fukuda, M. (2016). Rabin8 regulates neurite outgrowth in both GEF activity-dependent and -independent manners. Mol. Biol. Cell 27, 2107-2118. doi: 10.1091/mbc.E16-02-0091.

49. Honda, A., Al-Awar, O. S., Hay, J. C., Donaldson, J. G. (2005). Targeting of ARF-1 to the early Golgi by membrin, an ER-Golgi SNARE. J. Cell Biol. 168, 1039-1051. doi: 10.1083/jcb.200409138.

50. Hsu, V. W., Prekeris, R. (2010). Transport at the recycling endosome. Curr. Opin. Cell Biol. 22, 528-534. doi:10.1016/j.ceb.2010.05.008.

51. Humphries, W. H., Szymanski, C. J., Payne, C. K. (2011). Endo-lysosomal vesicles positive for Rab7 and LAMP1 are terminal vesicles for the transport of dextran. PLoS One 6, e26626. doi: 10.1371/journal.pone.0026626.

52. Imai, A., Yoshie, S., Ishibashi, K., Haga-Tsujimura, M., Nashida, T., Shimomura, H., Fukuda, M. (2011). EPI64 protein functions as a physiological GTPase-activating protein for Rab27 protein and regulates amylase release in rat parotid acinar cells. J. Biol. Chem. 286, 33854-33862. doi: 10.1074/jbc.M111.281394.

53. Ioannou, M. S., Girard, M., McPherson, P. S. (2016). Rab13 traffics on vesicles independent of prenylation. J. Biol Chem. 291, 10726-10735. doi:10.1074/jbc.M116.722298.

54. Ishizaki, R., Shin, H., Mitsuhashi, H., Nakayama, K. (2008). Redundant roles of BIG2 and BIG1, guanine-nucleotide exchange factors for ADP-ribosylation factors in membrane traffic between the trans-Golgi network and endosomes. Mol. Biol. Cell 19, 2650–2660. doi: 10.1091/mbc.e07-10-1067.

55. Itakura, E., Mizushima, N. (2011). p62 Targeting to the autophagosome formation site requires self-oligomerization but not LC3 binding. J. Cell Biol. 192, 17-27. doi: 10.1083/jcb.201009067.

56. Itoh, T., Fukuda, M. (2006). Identification of EPI64 as a GTPase-activating protein specific for Rab27A. J. Biol. Chem. 281, 31823-31831. doi: 10.1074/jbc.M603808200.

57. Jackson, T. R., Brown, F. D., Nie, Z., Miura, K., Foroni, L., Sun, J., et al. (2000). ACAPs are ARF6 GTPase-activating proteins that function in the cell periphery. J. Cell Biol. 151, 627-638. doi: 10.1083/jcb.151.3.627.

58. Janvier, K., Bonifacino, J. S. (2005). Role of the endocytic machinery in the sorting of lysosome-associated membrane proteins. Mol. Biol. Cell 16, 4231-4242. doi: 10.1091/mbc.e05-03-0213.

59. Jimah, J. R., Hinshaw, J. E. (2019). Structural insights into the mechanism of dynamin superfamily proteins. Trends Cell Biol. 29, 257-273. doi: 10.1016/j.tcb.2018.11.003.

60. Jović, M., Naslavsky, N., Rapaport, D., Horowitz, M., Caplan, S. (2007). EHD1 regulates beta1 integrin endosomal transport: effects on focal adhesions, cell spreading and migration. J. Cell Sci. 120, 802-814. doi: 10.1242/jcs.03383.
61. Junutula, J. R., De Maziére, A. M., Peden, A. A., Ervin, K. E., Advani, R. J., van Dijk, S. M., *et al.* (2004). Rab14 is involved in membrane trafficking between the Golgi complex and endosomes. Mol. Biol. Cell 15, 2218-2229. doi: 10.1091/mbc.e03-10-0777.
62. Kalaidzidis, I., Miaczynska, M., Brewinska-Olchowik, M., Hupalowska, A., Ferguson, C., Parton, R. G., *et al.* (2015). APPL endosomes are not obligatory endocytic intermediates but act as stable cargo-sorting compartments. J. Cell Biol. 211, 123–144. doi: 10.1083/jcb.201311117.
63. King, G. J., Stöckli, J., Hu, S. H., Winnen, B., Duprez, W. G., Meoli, C. C., *et al.* (2012) Membrane curvature protein exhibits interdomain flexibility and binds a small GTPase. J. Biol. Chem. 287, 40996-41006. doi: 10.1074/jbc.M112.349803.
64. Klein, S., Franco, M., Chardin, P., Luton, F. (2006). Role of the ARF6 GDP/GTP cycle and ARF6 GTPase-activating proteins in actin remodeling and intracellular transport. J. Biol. Chem 281, 12352-12361. doi: 10.1074/jbc.M601021200.
65. Klinkert, K., Echard, A. (2016). Rab35 GTPase: a central regulator of phosphoinositides and F-actin in endocytic recycling and beyond. Traffic 17, 1063-1077. doi: 10.1111/tra.12422.
66. Knödler, A., Feng, S., Zhang, J., Zhang, X., Das, A., Peränen, J., Guo, W. (2010). Coordination of Rab8 and Rab11 in primary ciliogenesis. Proc. Natl. Acad. Sci. USA 107, 6346-6351. doi: 10.1073/pnas.1002401107.
67. Kobayashi, H., Etoh, K., Ohbayashi, N., Fukuda, M. (2014). Rab35 promotes the recruitment of Rab8, Rab13 and Rab36 to recycling endosomes through MICAL-L1 during neurite outgrowth. Biol. Open 3, 803-814. doi: 10.1242/bio.20148771.
68. Kobayashi, H., Fukuda, M. (2012). Rab35 regulates ARF6 activity through centaurin beta2/ACAP2 during neurite outgrowth. J. Cell Sci. 125, 2235-2243. doi: 10.1242/jcs.098657.
69. Kobayashi, H., Fukuda, M. (2013). ARF6, Rab11 and transferrin receptor define distinct populations of recycling endosomes. Commun. Integr. Biol. 6, e25036. doi:10.4161/cib.25036.
70. Kondo, Y., Hanai, A., Nakai, W., Katoh, Y., Nakayama, K., Shin, H. W. (2012). ARF1 and ARF3 are required for the integrity of recycling endosomes and the recycling pathway. Cell Struct. Funct. 37, 141-154. doi: 10.1247/csf.12015.
71. Koukourakis, M. I., Kalamida, D., Giatromanolaki, A., Zois, C. E., Sivridis, E., Pouliliou, S., *et al.* (2015). Autophagosome proteins LC3A, LC3B and LC3C have distinct subcellular distribution kinetics and expression in cancer cell lines. PLoS One 10, e0137675. doi: 10.1371/journal.pone.0137675.
72. Kreykenbohm, V., Wenzel, D., Antonin, W., Atlachkine, V., Von Mollard, G. F. (2002). The SNAREs vti1a and vti1b have distinct localization and SNARE complex partners. Eur. J. Cell Biol. 81,273-280. PMID: 12067063.
73. Kucera, A., Borg Distefano, M., Berg-Larsen, A., Skjeldal, F., Repnik, U., Bakke, O., Progida, C. (2016). Spatiotemporal resolution of Rab9 and CI-MPR dynamics in the endocytic pathway. Traffic 17, 211-229. doi: 10.1111/tra.12357.
74. Kuma, A., Matsui, M., Mizushima, N. (2007). LC3, an autophagosome marker, can be incorporated into protein aggregates independent of autophagy: caution in the interpretation of LC3 localization. Autophagy. (4):323-8. doi: 10.4161/auto.4012.
75. Kumari, S., Mayor, S. (2008). ARF1 is directly involved in dynamin-independent endocytosis. Nat. Cell Biol. 10, 30-41. doi: 10.1038/ncb1666.
76. Lau, A. W., Chou, M.bM. (2008). The adaptor complex AP-2 regulates post-endocytic trafficking through the non-clathrin ARF6-dependent endocytic pathway. J. Cell Sci. 121, 4008-4017. doi: 10.1242/jcs.033522.
77. Laulagnier, K., Schieber, N. L., Maritzen, T., Haucke, V., Parton, R. G., Gruenberg, J. (2011). Role of AP1 and Gadkin in the traffic of secretory endo-lysosomes. Mol. Biol. Cell 22, 2068-2082. doi: 10.1091/mbc.E11-03-0193.
78. Lebrand, C., Corti, M., Goodson, H., Cosson, P., Cavalli, V., Mayran, N., *et al.* (2002). Late endosome motility depends on lipids via the small GTPase Rab7. EMBO J. 21, 1289-1300. doi: 10.1093/emboj/21.6.1289.
79. Li, J., Peters, P. J., Bai, M., Dai, J., Bos, E., Kirchhausen, T., *et al.* (2007). An ACAP1-containing clathrin coat complex for endocytic recycling. J. Cell Biol. 178, 453–464. doi: 10.1083/jcb.200608033.
80. Lin, S. X., Mallet, W. G., Huang, A. Y., Maxfield, F. R. (2004). Endocytosed cation-independent mannose 6-phosphate receptor traffics via the endocytic recycling compartment en route to the trans-Golgi network and a subpopulation of late endosomes. Mol. Biol. Cell 15, 721-733. doi: 10.1091/mbc.e03-07-0497.

81. Linford, A., Yoshimura, S., Nunes Bastos, R., Langemeyer, L, Gerondopoulos, A, Rigden, DJ, Barr, FA (2012). Rab14 and its exchange factor FAM116 link endocytic recycling and adherens junction stability in migrating cells. Dev. Cell. 22, 952-966. doi: 10.1016/j.devcel.2012.04.010.

82. Liu, H., Wang, S., Hang, W., Gao, J., Zhang, W., Cheng, Z., *et al.* (2018). LET-413/Erbin acts as a RAB-5 effector to promote RAB-10 activation during endocytic recycling. J. Cell Biol. 217, 299-314. doi: 10.1083/jcb.201705136.

83. Liu, O., Grant, B. D. (2015). Basolateral endocytic recycling requires RAB-10 and AMPH-1 mediated recruitment of RAB-5 GAP TBC-2 to endosomes. PLoS Genet. 11, e1005514. doi: 10.1371/journal.pgen.1005514.

84. Liu, S., Hunt, L., Storrie, B. (2013). Rab41 is a novel regulator of Golgi apparatus organization that is needed for ER-to-Golgi trafficking and cell growth. PLoS One 8, e71886. doi: 10.1371/journal.pone.0071886.

85. Liu, S., Storrie, B. (2015). How Rab proteins determine Golgi structure. Int. Rev. Cell Mol. Biol 315, 1-22. doi: 10.1016/bs.ircmb.2014.12.002.

86. Liu, Y., Xu, X. H., Chen, Q., Wang, T., Deng, C.Y., Song, B. L., *et al.* (2013). Myosin Vb controls biogenesis of post-Golgi Rab10 carriers during axon development. Nat. Commun. 4, 2005. doi: 10.1038/ncomms3005.

87. Lowery, J., Szul, T., Styers, M., Holloway, Z., Oorschot, V., Klumperman, J., Sztul, E. (2013). The Sec7 guanine nucleotide exchange factor GBF1 regulates membrane recruitment of BIG1 and BIG2 guanine nucleotide exchange factors to the trans-Golgi network (TGN). J. Biol Chem. 288, 11532-11545. doi: 10.1074/jbc.M112.438481.

88. Mahmutefendić, H., Blagojević, G., Tomaš, M. I., Kučić, N., Lučin, P. (2011). Segregation of open Major Histocompatibility Class I conformers at the plasma membrane and during endosomal trafficking reveals conformation-based sorting in the endosomal system. Int. J. Biochem. Cell Biol. 43, 504-515. doi:10.1016/j.biocel.2010.12.002.

89. Mahmutefendić, H., Blagojević Zagorac, G., Grabušić, K., Karleuša, L., Maćešić, S., Momburg, F., Lučin, P. (2017). Late endosomal recycling of open MHC-I conformers. J. Cell Physiol. 232, 872-887. doi: 10.1002/jcp.25495.

90. Mallard, F., Tang, B. L., Galli, T., Tenza, D., Saint-Pol, A., Yue, X., *et al.* (2002). Early/recycling endosomes-to-TGN transport involves two SNARE complexes and a Rab6 isoform. J. Cell Biol. 156, 653-664. doi: 10.1083/jcb.200110081.

91. Mallet, W. G., Maxfield, F. R. (1999). Chimeric forms of furin and TGN38 are transported with the plasma membrane in the trans-Golgi network via distinct endosomal pathways. J. Cell Biol. 146, 345-359. doi: 10.1083/jcb.146.2.345.

92. Manolea, F., Chun, J., Chen, D. W., Clarke, I., Summerfeldt, N., Dacks, J. B., Melançon, P. (2010). ARF3 is activated uniquely at the trans-Golgi network by brefeldin A-inhibited guanine nucleotide exchange factors. Mol. Biol. Cell. 21, 1836-1849. doi: 10.1091/mbc.E10-01-0016.

93. Mari, M., Macia, E., Le Marchand-Brustel, Y., Cormont, M. (2001). Role of the FYVE finger and the RUN domain for the subcellular localization of Rabip4. J. Biol. Chem. 276, 42501-42508. doi: 10.1074/jbc.M104885200.

94. Marra, P., Maffucci, T., Daniele, T., Tullio, G. D., Ikehara, Y., Chan, E. K., Luini, A., *et al.* (2001). The GM130 and GRASP65 Golgi proteins cycle through and define a subdomain of the intermediate compartment. Nat. Cell Biol. 3, 1101-1113. doi: 10.1038/ncb1201-1101.

95. Marzesco, A. M., Galli, T., Louvard, D., Zahraoui, A. (1998). The rod cGMP phosphodiesterase delta subunit dissociates the small GTPase Rab13 from membranes. J. Biol. Chem. 273, 22340-22345. doi: 10.1074/jbc.273.35.22340.

96. Massignan, T, Biasini, E, Lauranzano, E, Veglianese, P, Pignataro, M, Fioriti, L, *et al.* (2010). Mutant prion protein expression is associated with an alteration of the Rab GDP dissociation inhibitor alpha (GDI)/Rab11 pathway. Mol. Cell Proteomics 9, 611-622. doi: 10.1074/mcp.M900271-MCP200.

97. Matsudaira, T., Mukai, K., Noguchi, T., Hasegawa, J., Hatta, T., Iemura, S. I., *et al.* (2017). Endosomal phosphatidylserine is critical for the YAP signalling pathway in proliferating cells. Nat. Commun. 8, 1246. doi: 10.1038/s41467-017-01255-3.

98. Matsudaira, T., Niki, T., Taguchi, T., Arai, H. (2015). Transport of the cholera toxin B-subunit from recycling endosomes to the Golgi requires clathrin and AP-1. J. Cell Sci. 128, 3131-3142. doi: 10.1242/jcs.172171.

99. Mazelova, J., Astuto-Gribble, L., Inoue, H., Tam, B. M., Schonteich, E., Prekeris, R., *et al.* (2009). Ciliary targeting motif VxPx directs assembly of a trafficking module through ARF4. EMBO J. 28, 183-192. doi: 10.1038/emboj.2008.267.

100. McCullough, J., Colf, L. A., Sundquist, W. I. (2013). Membrane fission reactions of the mammalian ESCRT pathway. Annu. Rev. Biochem. 82, 663-692. doi: 10.1146/annurev-biochem-072909-101058.

101. McKenzie, J. E., Raisley, B., Zhou, X., Naslavsky, N., Taguchi, T., Caplan, S., Sheff, D. (2012). Retromer guides STxB and CD8-M6PR from early to recycling endosomes, EHD1 guides STxB from recycling endosome to Golgi. Traffic 13, 1140-1159. doi:10.1111/j.1600-0854.2012.01374.x.

102. McNally, K. E., Cullen, P. J. (2018). Endosomal retrieval of cargo: Retromer is not alone. Trends Cell Biol. 28, 807-822. doi: 10.1016/j.tcb.2018.06.005.

103. Mesaki, K., Tanabe, K., Obayashi, M., Oe, N., Takei, K. (2011). Fission of tubular endosomes triggers endosomal acidification and movement. PLoS One 6, 19764. doi:10.1371/journal.pone.0019764

104. Möbius, W., Herzog, V., Sandhoff, K., Schwarzmann, G. (1999). Intracellular distribution of a biotin-labeled ganglioside, GM1, by immunoelectron microscopy after endocytosis in fibroblasts. J. Histochem. Cytochem. 47, 1005-1014. doi: 10.1177/002215549904700804.

105. Moravec, R., Conger, K. K., D'Souza, R., Allison, A. B., Casanova, J. E. (2012). BRAG2/GEP100/IQSec1 interacts with clathrin and regulates α5β1 integrin endocytosis through activation of ADP ribosylation factor 5 (ARF5). J. Biol. Chem. 287, 31138-31147. doi: 10.1074/jbc.M112.383117.

106. Nakai, W., Kondo, Y., Saitoh, A., Naito, T., Nakayama, K., Shin, H. W. (2013). ARF1 and ARF4 regulate recycling endosomal morphology and retrograde transport from endosomes to the Golgi apparatus. Mol. Biol. Cell 24, 2570-2581. doi: 10.1091/mbc.E13-04-0197.

107. Naslavsky, N., Caplan, S. (2018). The enigmatic endosome – sorting the ins and outs of endocytic trafficking. J. Cell Sci. 131, jcs216499. doi: 10.1242/jcs.216499.

108. Naslavsky, N., Boehm, M., Backlund, P. S., Jr., Caplan, S. (2004). Rabenosyn-5 and EHD1 interact and sequentially regulate protein recycling to the plasma membrane. Mol. Biol. Cell 15, 2410-2422 doi: 10.1091/mbc.e03-10-0733.

109. Naslavsky, N., McKenzie, J., Altan-Bonnet, N., Sheff, D., Caplan, S. (2009). EHD3 regulates early-endosome-to-Golgi transport and preserves Golgi morphology. J. Cell Sci. 122, 389-400. doi: 10.1242/jcs.037051.

110. Navaroli, D. M., Stevens, Z. H., Uzelac, Z., Gabriel, L., King, M. J., Lifshitz, L. M., *et al.* (2011). The plasma membrane-associated GTPase Rin interacts with the dopamine transporter and is required for protein kinase C-regulated dopamine transporter trafficking. J. Neurosci. 31, 13758-13770. doi: 10.1523/JNEUROSCI.2649-11.2011.

111. Ng, E. L., Wang, Y., Tang, B. L. (2007). Rab22B's role in trans-Golgi network membrane dynamics. Biochem. Biophys. Res. Commun. 361, 751-757. doi: 10.1016/j.bbrc.2007.07.076.

112. Nielsen, E., Christoforidis, S., Uttenweiler-Joseph, S., Miaczynska, M., Dewitte, F., Wilm, M., *et al.* (2000). Rabenosyn-5, a novel Rab5 effector, is complexed with hVPS45 and recruited to endosomes through a FYVE finger domain. J Cell Biol. 151, 601-612. doi: 10.1083/jcb.151.3.601.

113. Nokes, R. L., Fields, I. C., Collins, R. N., Fölsch, H. (2008). Rab13 regulates membrane trafficking between TGN and recycling endosomes in polarized epithelial cells. J. Cell Biol. 182, 845-853. doi: 10.1083/jcb.200802176.

114. Norberg, E., Orrenius, S., Zhivotovsky, B. (2010). Mitochondrial regulation of cell death: processing of apoptosis-inducing factor (AIF). Biochem. Biophys. Res. Commun. 396, 95-100. doi: 10.1016/j.bbrc.2010.02.163.

115. Nottingham, R. M., Pusapati, G. V., Ganley, I. G., Barr, F. A., Lambright, D. G., Pfeffer, S. R. (2012). RUTBC2 protein, a Rab9A effector and GTPase-activating protein for Rab36. J. Biol. Chem 287, 22740-22748. doi: 10.1074/jbc.M112.362558.

116. Overmeyer, J. H., Wilson, A. L., Maltese, W. A. (2001). Membrane targeting of a Rab GTPase that fails to associate with Rab escort protein (REP) or guanine nucleotide dissociation inhibitor (GDI). J. Biol. Chem. 276, 20379-20386. doi: 10.1074/jbc.M101511200.

117. Paleotti, O., Macia, E., Luton, F., Klein, S., Partisani, M., Chardin, P., *et al.* (2005). The small G-protein ARF6GTP recruits the AP-2 adaptor complex to membranes. J. Biol. Chem. 280, 21661-21666. doi: 10.1074/jbc.M503099200.

118. Park, S. Y., Guo, X. (2014). Adaptor protein complexes and intracellular transport. Biosci. Rep. 34, pii: e00123. doi: 10.1042/BSR20140069.

119. Patino-Lopez, G., Dong, X., Ben-Aissa, K., Bernot, K. M., Itoh, T., Fukuda, M., *et al.* (2008). Rab35 and its GAP EPI64C in T cells regulate receptor recycling and immunological synapse formation. J. Biol. Chem. 283, 8323-8330. doi: 10.1074/jbc.M800056200.

120. Peränen, J. (2011). Rab8 GTPase as a regulator of cell shape. Cytoskeleton (Hoboken). 68, 527-539. doi:10.1002/cm.20529.

121. Pérez-Victoria, F. J., Bonifacino, J. S. (2009). Dual roles of the mammalian GARP complex in tethering and SNARE complex assembly at the trans-golgi network. Mol. Cell Biol. 29, 5251-5263. doi: 10.1128/MCB.00495-09.
122. Pols, M. S., Klumperman, J. (2009). Trafficking and function of the tetraspanin CD63. Exp. Cell Res. 315, 1584-1592. doi: 10.1016/j.yexcr.2008.09.020.
123. Poteryaev, D., Datta, S., Ackema, K., Zerial, M., Spang, A. (2010). Identification of the switch in early-to-late endosome transition. Cell 141, 497-508. doi: 10.1016/j.cell.2010.03.011.
124. Prekeris, R. (2012). The art of "cut and run": the role of Rab14 GTPase in regulating N-cadherin shedding and cell motility. Dev Cell. 22, 909-910. doi:10.1016/j.devcel.2012.05.002.
125. Proikas-Cezanne, T., Gaugel, A., Frickey, T., Nordheim, A. (2006). Rab14 is part of the early endosomal clathrin-coated TGN microdomain. FEBS Lett. 580, 5241-5246. doi: 10.1016/j.febslet.2006.08.053.
126. Pylypenko, O., Hammich, H., Yu, I. M., Houdusse, A. (2018). Rab GTPases and their interacting protein partners: Structural insights into Rab functional diversity. Small GTPases. 9:1-2, 22-48, doi: 10.1080/21541248.2017.1336191.
127. Rahajeng, J., Panapakkam Giridharan, S. S., Cai, B., Naslavsky, N., Caplan, S. (2012). MICAL-L1 is a tubular endosomal membrane hub that connects Rab35 and ARF6 with Rab8a. Traffic 13, 82-93. doi: 10.1111/j.1600-0854.2011.01294.x.
128. Rai, A., Goody, R. S., Müller, M. P. (2019). Multivalency in Rab effector interactions. Small GTPases 10, 40-46. doi: 10.1080/21541248.2016.1265700.
129. Rai, A., Oprisko, A., Campos, J., Fu, Y., Friese, T., Itzen, A., et al. (2016). bMERB domains are bivalent Rab8 family effectors evolved by gene duplication. Elife 5 Pii, e18675. doi: 10.7554/eLife.18675.
130. Raiborg, C., Bache, K. G., Mehlum, A., Stenmark, H. (2001). Function of Hrs in endocytic trafficking and signalling. Biochem. Soc. Trans. 29, 472-475. doi: 10.1042/bst0290472.
131. Raposo, G., Marks, M. S., Cutler, D. F. (2007). Lysosome-related organelles: driving post-Golgi compartments into specialisation. Curr. Opin. Cell Biol. 19, 394-401. doi: 10.1016/j.ceb.2007.05.001.
132. Reverter, M., Rentero, C., Garcia-Melero, A., Hoque, M., Vilà de Muga, S., Alvarez-Guaita, A., *et al.* (2014). Cholesterol regulates Syntaxin 6 trafficking at trans-Golgi network endosomal boundaries. Cell Rep. 7, 883-97. doi: 10.1016/j.celrep.2014.03.043.
133. Rink, J., Ghigo, E., Kalaidzidis, Y., Zerial, M. (2005). Rab conversion as a mechanism of progression from early to late endosomes. Cell. 122, 735–749. doi: 10.1016/j.cell.2005.06.043.
134. Rodriguez-Gabin, A. G., Yin, X., Si, Q., Larocca, J. N. (2009). Transport of mannose-6-phosphate receptors from the trans-Golgi network to endosomes requires Rab31. Exp. Cell Res. 315, 2215-2230. doi: 10.1016/j.yexcr.2009.03.020.
135. Rojas, R., van Vlijmen, T., Mardones, G. A., Prabhu, Y., Rojas, A. L., Mohammed, S., *et al.* (2008). Regulation of retromer recruitment to endosomes by sequential action of Rab5 and Rab7. J. Cell Biol. 183, 513-526. doi: 10.1083/jcb.200804048.
136. Sakane, A., Honda, K., Sasaki, T. (2010). Rab13 regulates neurite outgrowth in PC12 cells through its effector protein, JRAB/MICAL-L2. Mol. Cell Biol. 30, 1077-1087. doi: 10.1128/MCB.01067-09.
137. Sano, H., Roach, W. G., Peck, G. R., Fukuda, M., Lienhard, G. E. (2008). Rab10 in insulin-stimulated GLUT4 translocation. Biochem. J. 411, 89-95. doi: 10.1042/BJ20071318
138. Sasidharan, N., Sumakovic, M., Hannemann, M., Hegermann, J., Liewald, J. F., Olendrowitz, C., *et al.* (2012). RAB-5 and RAB-10 cooperate to regulate neuropeptide release in Caenorhabditis elegans. Proc. Natl. Acad. Sci. USA 109, 18944-18949. doi: 10.1073/pnas.1203306109.
139. Schindler, C., Chen, Y., Pu, J., Guo, X., Bonifacino, J. S. (2015). EARP is a multisubunit tethering complex involved in endocytic recycling. Nat. Cell Biol. 17, 639-650. doi: 10.1038/ncb3129.
140. Schöneberg, J., Lee, I. H., Iwasa, J. H., Hurley, J. H. (2017). Reverse-topology membrane scission by the ESCRT proteins. Nat. Rev. Mol. Cell Biol. 18, 5-17. doi: 10.1038/nrm.2016.121.
141. Seaman, M. N., Harbour, M. E., Tattersall, D., Read, E., Bright, N. (2009). Membrane recruitment of the cargo-selective retromer subcomplex is catalysed by the small GTPase Rab7 and inhibited by the Rab-GAP TBC1D5. J. Cell Sci. 122, 2371-2382. doi: 10.1242/jcs.048686.

142. Shakya, S., Sharma, P., Bhatt, A. M., Jani, R. A., Delevoye, C., Setty, S. R. (2018). Rab22A recruits BLOC-1 and BLOC-2 to promote the biogenesis of recycling endosomes. EMBO Rep. 19(12). pii: e45918. doi: 10.15252/embr.201845918.
143. Sharma, M., Giridharan, S. S., Rahajeng, J., Naslavsky, N., Caplan, S. (2009). MICAL-L1 links EHD1 to tubular recycling endosomes and regulates receptor recycling. Mol. Biol. Cell 20, 5181-5194. doi: 10.1091/mbc.e09-06-0535
144. Shen, X., Xu, K. F., Fan, Q., Pacheco-Rodriguez, G., Moss, J., Vaughan, M. (2006). Association of brefeldin A-inhibited guanine nucleotide-exchange protein 2 (BIG2) with recycling endosomes during transferrin uptake. Proc. Natl. Acad. Sci. USA 103, 2635-2640. doi: 10.1073/pnas.0510599103.
145. Shi, A., Grant, B. D. (2013). Interactions between Rab and ARF GTPases regulate endosomal phosphatidylinositol-4,5-bisphosphate during endocytic recycling. Small GTPases 4. 106-109. doi: 10.4161/sgtp.23477.
146. Shi, A., Liu, O., Koenig, S., Banerjee, R., Chen, C. C., Eimer, S., Grant, B. D. (2012). RAB-10-GTPase-mediated regulation of endosomal phosphatidylinositol-4,5-bisphosphate. Proc. Natl. Acad. Sci. USA 109, E2306-15. doi: 10.1073/pnas.1205278109.
147. Shin, H., Morinaga, N., Noda, M., Nakayama, K. (2004). BIG2, A guanine nucleotide exchange factor for ADP-ribosylation factors: Its localization to recycling endosomes and implication in the endosome integrity. Mol. Biol. Cell 15, 5283–5294. doi: 10.1091/mbc.e04-05-0388.
148. Shisheva, A. (2008). PIKfyve: Partners, significance, debates and paradoxes. Cell Biol. Int. 32, 591-604. doi: 10.1016/j.cellbi.2008.01.006.
149. Shteyn, E., Pigati, L., Folsch, H. (2011). ARF6 regulates AP-1B-dependent sorting in polarized epithelial cells. J. Cell Biol. 194, 873–887. doi: 10.1083/jcb.201106010.
150. Simonsen, A., Gaullier, J. M., D’Arrigo, A., Stenmark, H. (1999). The Rab5 effector EEA1 interacts directly with syntaxin-6. J. Biol. Chem. 274, 28857-28860. doi: 10.1074/jbc.274.41.28857.
151. Simonsen, A., Lippe, R., Christoforidis, S., Gaullier, J. M., Brech, A., Callaghan, J., *et al.* (1998). EEA1 links PI(3)K function to Rab5 regulation of endosome fusion. Nature 394, 494-498. doi: 10.1038/28879.
152. Sönnichsen, B., De Renzis, S., Nielsen, E., Rietdorf, J., Zerial, M. (2000). Distinct membrane domains on endosomes in the recycling pathway visualized by multicolor imaging of Rab4, Rab5, and Rab11. J. Cell Biol. 149, 901–914. doi: 10.1083/jcb.149.4.901.
153. Sorkina, T., Bild, A., Tebar, F., Sorkin, A. (1999). Clathrin, adaptors and eps15 in endosomes containing activated epidermal growth factor receptors. J. Cell Sci. 112, 317-327. PMID: 9885285.
154. Taguchi, T. (2013). Emerging roles of recycling endosomes. J. Biochem. 153, 505-510. doi: 10.1093/jb/mvt034.
155. Tai, G., Lu, L., Wang, T. L., Tang, B. L., Goud, B., Johannes L., Hong, W. (2004). Participation of the syntaxin 5/Ykt6/GS28/GS15 SNARE complex in transport from the early/recycling endosome to the trans-Golgi network. Mol. Biol. Cell 15, 4011-4022. doi: 10.1091/mbc.e03-12-0876.
156. Takahashi, S., Kubo, K., Waguri, S., Yabashi, A., Shin, H. W., Katoh, Y., Nakayama, K. (2012). Rab11 regulates exocytosis of recycling vesicles at the plasma membrane. J. Cell Sci. 125, 4049-4057. doi: 10.1242/jcs.102913.
157. Thomas, G. (2002). Furin at the cutting edge: from protein traffic to embryogenesis and disease. Nat. Rev. Mol. Cell Biol. 3, 753-766. doi: 10.1038/nrm934.
158. Uchida, Y., Hasegawa, J., Chinnapen, D., Inoue, T., Okazaki, S., Kato, R., *et al.* (2011). Intracellular phosphatidylserine is essential for retrograde membrane traffic through endosomes. Proc. Natl. Acad. Sci. USA 108, 15846-15851. doi: 10.1073/pnas.1109101108.
159. Valdivia, R. H., Baggott, D., Chuang, J.S., Schekman, R. W. (2002). The yeast clathrin adaptor protein complex 1 is required for the efficient retention of a subset of late Golgi membrane proteins. Dev Cell 2, 283-294. doi: 10.1016/s1534-5807(02)00127-2.
160. van Dam, E. M., Stoorvogel, W. (2002). Dynamin-dependent transferrin receptor recycling by endosome-derived clathrin-coated vesicles. Mol. Biol. Cell. 13, 169-182. doi: 10.1091/mbc.01-07-0380.
161. Van der Kant, R., Zondervan, I., Janssen, L., Neefjes, J. (2013). Cholesterol-binding molecules MLN64 and ORP1L mark distinct late endosomes with transporters ABCA3 and NPC1. J Lipid Res. 54, 2153-2165. doi: 10.1194/jlr.M037325.
162. Vanlandingham, P. A., Ceresa, B. P. (2009). Rab7 regulates late endocytic trafficking downstream of multivesicular body biogenesis and cargo sequestration. J. Biol. Chem. 284, 12110–12124. doi: 10.1074/jbc.M809277200.

163. Vetter, M., Stehle, R., Basquin, C., Lorentzen, E. (2015). Structure of Rab11-FIP3-Rabin8 reveals simultaneous binding of FIP3 and Rabin8 effectors to Rab11. Nat. Struct. Mol. Biol. 22, 695-702. doi: 10.1038/nsmb.3065.
164. Villaseñor, R., Kalaidzidis, Y., Zerial, M. (2016). Signal processing by the endosomal system. Curr. Opin. Cell Biol. 39:53-60. doi: 10.1016/j.ceb.2016.02.002.
165. Volpicelli-Daley, L. A., Li, Y., Zhang, C. J., Kahn, R. A. (2005). Isoform-selective effects of the depletion of ADP-ribosylation factors 1-5 on membrane traffic. Mol. Biol. Cell 16, 4495-4508. doi: 10.1091/mbc.e04-12-1042.
166. Wang, J., Fedoseienko, A., Chen, B., Burstein, E., Jia, D., Billadeau, D. D. (2018). Endosomal receptor trafficking: retromer and beyond. Traffic 19,578-590. doi:10.1111/tra.12574.
167. Wang, P., Liu, H., Wang, Y., Liu, O., Zhang, J., Gleason, A., *et al.* (2016). RAB-10 promotes EHBP-1 bridging of filamentous actin and tubular recycling endosomes. PLoS Genet. 12, e1006093. doi: 10.1371/journal.pgen.1006093.
168. Watson, R. T., Pessin, J. E. (2000). Functional cooperation of two independent targeting domains in syntaxin 6 is required for its efficient localization in the trans-Golgi network of 3T3L1 adipocytes. J. Biol. Chem. 275, 1261-1268. doi: 10.1074/jbc.275.2.1261.
169. Weekes, M. P., Tomasec, P., Huttlin, E. L., Fielding, C. A., Nusinow, D., Stanton, R. J., *et al.* (2014). Quantitative temporal viromics: an approach to investigate hostpathogen interaction. Cell 157, 1460 –1472. doi: 10.1016/j.cell.2014.04.028.
170. Whitley, P., Reaves, B. J., Hashimoto, M., Riley, A. M., Potter, B. V., Holman, G. D. (2003). Identification of mammalian Vps24p as an effector of phosphatidylinositol 3,5-bisphosphate-dependent endosome compartmentalization. J. Biol. Chem. 278, 38786-38795. doi: 10.1074/jbc.M306864200.
171. Wilson, J. M., de Hoop, M., Zorzi, N., Toh, B. H., Dotti, C. G., Parton, R. G. (2000). EEA1, a tethering protein of the early sorting endosome, shows a polarized distribution in hippocampal neurons, epithelial cells, and fibroblasts. Mol. Biol. Cell 11, 2657-2671. doi: 10.1091/mbc.11.8.2657.
172. Witkos, T. M., Lowe, M. (2016). The Golgin family of coiled-coil tethering proteins. Front. Cell Dev. Biol. 3, 86. doi: 10.3389/fcell.2015.00086.
173. Xu, Y., Martin, S., James, D. E., Hong, W. (2002). GS15 forms a SNARE complex with syntaxin 5, GS28, and Ykt6 and is implicated in traffic in the early cisternae of the Golgi apparatus. Mol. Biol. Cell 13, 3493-3507. doi: 10.1091/mbc.e02-01-0004.
174. Yamamoto, H., Koga, H., Katoh, Y., Takahashi, S., Nakayama, K., Shin, H. W. (2010). Functional cross-talk between Rab14 and Rab4 through a dual effector, RUFY1/Rabip4. Mol. Biol. Cell 21, 2746-2455. doi: 10.1091/mbc.E10-01-0074.
175. Yang, C. Z., Heimberg, H., D'Souza-Schorey, C., Mueckler, M. M., Stahl, P. D. (1998). Subcellular distribution and differential expression of endogenous ADP-ribosylation factor 6 in mammalian cells. J. Biol. Chem. 273, 4006-4011. doi: 10.1074/jbc.273.7.4006.
176. Zagorac, G. B., Mahmutefendić, H., Tomaš, M. I., Kučić, N., Le Bouteiller, P., Lučin, P. (2012). Early endosomal rerouting of major histocompatibility class I conformers. J. Cell. Physiol. 227, 2953-2964. doi: 10.1002/jcp.23042.
177. Zhao, X., Lasell, T. K. R., Melancon, P. (2002). Localization of large ADP-ribosylation factor- guanine nucleotide exchange factors to different Golgi compartments: evidence for distinct functions in protein. Mol. Cell Biol. 13, 119–133. doi: 10.1091/mbc.01-08-0420.
178. Zhu, H., Liang, Z., Li, G. (2009). Rabex-5 is a Rab22 effector and mediates a Rab22-Rab5 signaling cascade in endocytosis. Mol. Biol. Cell. 20, 4720–4729. doi: 10.1091/mbc.e09-06-0453.
179. Zoncu, R., Perera, R.M., Balkin, D. M., Pirruccello, M., Toomre, D., De Camilli, P. (2009). A phosphoinositide switch controls the maturation and signaling properties of APPL endosomes. Cell 136, 1110–1121. doi: 10.1016/j.cell.2009.01.032.
180. Zuk, P. A., Elferink, L. A. (2000). Rab15 differentially regulates early endocytic trafficking. J. Biol. Chem. 275, 26754-26764. doi: 10.1074/jbc.M000344200.
