# Supplementary material for: Cytomegalovirus Generates Assembly Compartment in the Early Phase of Infection by Perturbation of Host-Cell Factors Recruitment at the Early Endosome/Endosomal Recycling Compartment/Trans-Golgi Interface
Source: Front Cell Dev Biol. 2020 Sep 11;8:563607. doi: 10.3389/fcell.2020.563607 (PMC7516400; doi:10.3389/fcell.2020.563607)
Supplement: Supplementary file 3 [file Data_Sheet_3.PDF]

Supplementary Material

Table S3. Guanine nucleotide exchange factors (GEFs) and GTP-ase activating proteins (GAPs) of small GTPases that regulate EE-ERC-TGN interface (Related to Fig. 10)

Small GTPase	GEF	Reference	GAP	Reference
**Rab22a**	Rinl	Woller et al., 2011	RabGAP1L	Qu et al., 2016
	Rabex-5 (RABGEF1)	Delprato et al., 2004; Yoshimura et al., 2010; Ishida et al., 20016; Fukuda, 2016		
**Rab5a**	Rabex-5 (RABGEF1)	Horiuchi et al., 1997; Yoshimura et al., 2010; Ishida et al., 2016	TBC1D3	Fukuda, 2011; Müller and Goody, 2018
	Rin1	Tall et al., 2001; Chen et al., 2009; Balaji et al., 2014	RUTBC3 (SGSM1, RabGAP-5)	Haas et al., 2005; Fukuda, 2011; Müller and Goody, 2018
	Rin2	Saito et al., 2002; Kajiho et al., 2003; Carney et al., 2006	USP6NL (RN-Tre)	Fukuda, 2011; Müller and Goody, 2018
	Rin3	Kajiho et al., 2003	SGSM3	Gillingham et al., 2014
	Rinl	Woller et al., 2011; Kajiho et al., 2012		
	ALS2/Alsin	Topp et al., 2004;		
	ALS2CL	Hadano et al., 2004		
	Varp (ANKRD27)	Bean et al., 2015		
	RAP6 (GAPVD1, Gapex5, RME-6)	Hunker et al., 2006; Sato et al., 2006; Kitano et al., 2008; Smythe, 2015; Fukuda, 2016		
**Rab10**	DennD4a-c	Yoshimura et al., 2010; Ishida et al., 2016; Müller and Goody, 2018	TBC1D1	Roach et al. 2007; Frasa et al., 2012; Müller and Goody, 2018
	Rabin-8 (RAB3IP)	Homma and Fukuda, 2016	TBC1D4	Fukuda, 2011; Müller and Goody, 2018
			EVI5-L	Frasa et al., 2012; Müller and Goody, 2018
**Rab14**	DennD6a-b	Yoshimura et al., 2010; Linford et al., 2012; Ishida et al., 2016;	TBC1D1	Roach et al. 2007; Frasa et al., 2012; Müller and Goody, 2018
			TBC1D4	Fukuda, 2011; Müller and Goody, 2018

**Rab15**	NA		NA	
**Rab11a**	SH3BP5 (REI-1)	Sakaguchi et al., 2015; Müller and Goody, 2018; Jenkins et al., 2018; Goto-Ito et al., 2019	TBC1D11 (RABGAP1)	Fuchs et al., 2007; Fukuda, 2011; Müller and Goody, 2018
	SH3BP5L	Goto-Ito et al., 2019	TBC1D15	Zhang et al, 2005; Fukuda, 2011; Müller and Goody, 2018
			TBC1D9B	Gallo et al., 2014
			EVI5	Westlake et al., 2007; Fukuda, 2011; Müller and Goody, 2018
**Rab8a**	Rabin-8 (RAB3IP)	Hattula et al., 2002; Knödler et al., 2010; Yoshimura et al., 2010; Wang et al., 2015; Ishida et al., 2016; Homma and Fukuda, 2016; Pylypenko et al., 2018	TBC1D1	Roach et al. 2007; Frasa et al., 2012; Müller and Goody, 2018
	GRAB (Rab3IL1)	Yoshimura et al., 2010; Ishida et al., 2016; Müller and Goody, 2018	TBC1D30	Frasa et al., 2012; Müller and Goody, 2018
	Mss4 (RABIF)	Itzen et al., 2006; Müller and Goody, 2018	TBC1D4	Fukuda, 2011; Müller and Goody, 2018
	RPGR	Murga-Zamalloa et al., 2010	TBC1D17	Vaibhava et al, 2012
	C9orf72-SMCR8	Corbier et al., 2016; Müller and Goody, 2018	TBC1D10	Hokanson and Bretscher, 2012
**Rab35**	DennD1A (connecdenn 1)	Allaire et al., 2010; Marat et al., 2010; Marat et al., 2011; Yoshimura et al., 2010; Chaineau et al., 2013; Ishida et al., 2016; Cauvin et al., 2016;	TBC1D10A (EPI64A)	Hsu et al., 2010; Fukuda, 2011; Cauvin et al., 2016; Klinkert and Echard, 2016; Biesemann et al., 2017; Müller and Goody, 2018
	DennD1B (connecdenn 2)	Marat and McPherson, 2010; Yoshimura et al., 2010; Chaineau et al., 2013; Ishida et al., 2016; Müller and Goody, 2018; Pylypenko et al., 2018	TBC1D10B (EPI64B)	Fukuda, 2011; Müller and Goody, 2018 Hsu et al., 2010
	DennD1C (connecdenn 3)	Marat and McPherson, 2010; Allaire et al., 2010; Chaineau et al., 2013; Ishida et al., 2016; Müller and Goody, 2018	TBC1D10C (EPI64C)	Hsu et al., 2010; Fukuda, 2011; Müller and Goody, 2018
	Folliculin (FLCN)	Nookala et al., 2012; Chaineau et al., 2013; Ishida et al., 2016; Zheng et al., 2017	TBC1D17	Fukuda, 2011; Müller and Goody, 2018
			TBC1D13	Davey et al., 2012; Klinkert and Echard, 2016

			TBC1D24	Chaineau et al., 2013;
			EVI5	Dabbeekeh et al., 2007; Westlake et al., 2005; Fukuda, 2011; Müller and Goody, 2018
**ARF6**	EFA6a (Psd1)	Franco et al., 1999; Macia et al., 2001; Macia et al., 2012; Donaldson and Jackson, 2011	ACAP1	Jackson et al., 2000; Donaldson and Jackson, 2011; Chen et al., 2104
	EFA6b (Psd4)	Derrien et al., 2002; Donaldson and Jackson, 2011	ACAP2 (centaurin β2)	Jackson et al., 2000; Kanno et al., 2010; Donaldson and Jackson, 2011; Rahajeng et al., 2012; Biesemann et al., 2017
	EFA6c (Psd2)	Matsuya et al., 2005; Donaldson and Jackson, 2011; Saegusa et al., 2019	ACAP3	Jackson et al., 2000; Donaldson and Jackson, 2011
	EFA6d (Psd3)	Sakagami et al., 2006; Donaldson and Jackson, 2011	ARAP2	Chen et al., 2104
	BRAG2 (Iqsec1) (GEP100)	Someya et al., 2002; Dunphy et al., 2006; Hiroi et al., 2006; Donaldson and Jackson, 2011; Aizel et al., 2013	ARAP3	Donaldson and Jackson, 2011
	BRAG1 (Iqsec2)	Donaldson and Jackson, 2011	GIT1	Donaldson and Jackson, 2011
	BRAG3 (Iqsec3)	Donaldson and Jackson, 2011	GIT2	Donaldson and Jackson, 2011
	Cytohesin1 (CYTH1)	Casanova, 2007; Donaldson and Jackson, 2011	SMAP1	Donaldson and Jackson, 2011
	Cytohesin2 (CYTH2/ ARNO)	Macia et al., 2001; Casanova, 2007; Donaldson and Jackson, 2011	SMAP2	Donaldson and Jackson, 2011
	Cytohesin3 (Grp1)	Casanova, 2007; Donaldson and Jackson, 2011		
**Rab6a**	Ric1-Rgp1	Yoshimura et al., 2010; Pusapati et al., 2012; Ishida et al., 2016; Müller and Goody, 2018	TBC1D11 (GAPCenA)	Miserey-Lenkei et al., 2006; Fukuda, 2011; Müller and Goody, 2018
	RINT-1 (ZW10)	Ishida et al., 2016		
	Rab3GAP1-2	Ishida et al., 2016		
**Rab31/ 22b**	Rin1-3	Kajiho et al., 2011; Ishida et al., 2016	TBC1D10B	Frasa et al., 2012; Müller and Goody, 2018
	Rinl	Kajiho et al., 2012		

	ALS2/Alsin	Kajiho et al., 2011		
	ALS2CL	Kajiho et al., 2011		
	Gapex-5 (GAPVD1)	Lodhi et al., 2007; Fukuda, 2016		
**Rab9a**	DennD2a-d	Yoshimura et al., 2010; Ishida et al., 2016; Müller and Goody, 2018		
**Rab13**	DennD1C	Yoshimura et al., 2010; Ishida et al., 2016; Müller and Goody, 2018	TBC1D25	Frasa et al., 2012; Müller and Goody, 2018
	DennD2B	Ioannou et al., 2015;	TBC1D4	Sun et al., 2010; Nishikimi et al., 2014; Ioannou et al., 2015
**Rab36**	NA		TBC1D11	Fukuda, 2011; Müller and Goody, 2018
			RUTBC2 (SGSM1)	Nottingham et al., 2012

NA, not available.

Supplementary references:

1. Aizel, K., Biou, V., Navaza, J., Duarte, L. V., Campanacci, V., Cherfils, J., Zeghouf, M. (2013). Integrated conformational and lipid-sensing regulation of endosomal ArfGEF BRAG2. PLoS Biol. 11, e1001652. doi: 10.1371/journal.pbio.1001652.
2. Allaire, P. D., Marat, A. L., Dall'Armi, C., Di, P. G., McPherson, P. S., Ritter, B. (2010). The Connecdenn DENN domain: a GEF for Rab35 mediating cargo-specific exit from early endosomes. Mol. Cell 37, 370–382. doi: 10.1016/j.molcel.2009.12.037.
3. Balaji, K., French, C. T., Miller, J. F., Colicelli, J. (2014). The RAB5-GEF function of RIN1 regulates multiple steps during Listeria monocytogenes infection. Traffic 15, 1206-1218. doi: 10.1111/tra.12204.
4. Bean, B. D., Davey, M., Snider, J., Jessulat, M., Deineko, V., Tinney, M., et al. (2015). Rab5-family guanine nucleotide exchange factors bind retromer and promote its recruitment to endosomes. Mol. Biol. Cell. 26, 1119-1128. doi: 10.1091/mbc.E14-08-1281.
5. Biesemann, A., Gorontzi, A., Barr, F., Gerke, V. (2017). Rab35 protein regulates evoked exocytosis of endothelial Weibel-Palade bodies. J. Biol. Chem. 292, 11631-11640. doi: 10.1074/jbc.M116.773333.
6. Carney, D. S., Davies, B. A., Horazdovsky, B. F. (2006). Vps9 domain-containing proteins: activators of Rab5 GTPases from yeast to neurons. Trends Cell Biol. 16, 27-35. doi: 10.1016/j.tcb.2005.11.001.
7. Casanova, J. E. (2007). Regulation of Arf activation: the Sec7 family of guanine nucleotide exchange factors. Traffic 8, 1476–1485. doi: 10.1111/j.1600-0854.2007.00634.x
8. Cauvin, C., Rosendale, M., Gupta-Rossi, N., Rocancourt, M., Larraufie, P., Salomon, R., et al. (2016). Rab35 GTPase triggers switch-like recruitment of the lowe syndrome lipid phosphatase OCRL on newborn endosomes. Curr. Biol. 26, 120-128 doi: 10.1016/j.cub.2015.11.040.
9. Chaineau, M., Ioannou, M. S., McPherson, P. S. (2013). Rab35: GEFs, GAPs and effectors. Traffic14, 1109-1117. doi: 10.1111/tra.12096.
10. Chen, P. I., Kong, C., Su, X., Stahl, P. D. (2009). Rab5 isoforms differentially regulate the trafficking and degradation of epidermal growth factor receptors. J. Biol. Chem. 284, 30328-3038. doi: 10.1074/jbc.M109.034546.

11. Chen, P. W., Luo, R., Jian, X., Randazzo, P. A. (2014). The Arf6 GTPase-activating proteins ARAP2 and ACAP1 define distinct endosomal compartments that regulate integrin α5β1 traffic. J Biol Chem. 289, 30237-30248. doi: 10.1074/jbc.M114.596155.
12. Corbier, C., Sellier, C. (2016). C9ORF72 is a GDP/GTP exchange factor for Rab8 and Rab39 and regulates autophagy. Small GTPases 5, 1-6. PMID:27494456; doi: 10.1080/21541248.2016.1212688
13. Dabbeekeh, J. T. S., Faitar, S. L., Dufresne, C. P., Cowell, J. K. (2007). The EVI5 TBC domain provides the GTPase-activating protein motif for RAB11. Oncogene 26, 2804–2808. doi: 10.1038/sj.onc.1210081.
14. Davey, J. R., Humphrey, S. J., Junutula, J. R., Mishra, A. K., Lambright, D. G., James, D. E., Stockli, J. (2012). TBC1D13 is a RAB35 specific GAP that plays an important role in GLUT4 trafficking in adipocytes. Traffic 13, 1429–1441.
15. Delprato, A., Merithew, E., Lambright, D. G. (2004). Structure, exchange determinants, and family-wide rab specificity of the tandem helical bundle and Vps9 domains of Rabex-5. Cell 118, 607-617.
16. Derrien, V., Couillault, C., Franco, M., Martineau, S., Montcourrier, P., Houlgatte, R., Chavrier, P. (2002). A conserved C-terminal domain of EFA6-family ARF6-guanine nucleotide exchange factors induces lengthening of microvilli-like membrane protrusions. J. Cell Sci. 115, 2867-2879.
17. Donaldson, J. G., Jackson, C. L. (2011). ARF family G proteins and their regulators: roles in membrane transport, development and disease. Nat. Rev. Mol. Cell Biol. 12, 362-375. doi: 10.1038/nrm3117.
18. Dunphy, J. L., Moravec, R., Ly K., Lasell, T. K., Melancon, P., Casanova, J.E. (2006). The Arf6 GEF GEP100/BRAG2 regulates cell adhesion by controlling endocytosis of beta1 integrins. Curr. Biol. 16, 315–320. doi: 10.1016/j.cub.2005.12.032.
19. Esters, H., Alexandrov, K., Iakovenko, A., Ivanova, T., Thomä, N., Rybin, V., et al. (2001). Vps9, Rabex-5 and DSS4: proteins with weak but distinct nucleotide-exchange activities for Rab proteins. J. Mol. Biol. 310, 141-156. doi: 10.1006/jmbi.2001.4735.
20. Franco, M., Peters, P. J., Boretto, J., van Donselaar, E., Neri, A., D'Souza-Schorey, C., Chavrier, P. (1999). EFA6, a sec7 domain-containing exchange factor for ARF6, coordinates membrane recycling and actin cytoskeleton organization. EMBO J. 18, 1480-1491. doi: 10.1093/emboj/18.6.1480.
21. Frasa, M. A., Koessmeier, K. T., Ahmadian, M. R., Braga, V. M. (2012). Illuminating the functional and structural repertoire of human TBC/RABGAPs. Nat. Rev. Mol. Cell Biol. 13, 67-73. doi: 10.1038/nrm3267.
22. Fuchs, E., Haas, A. K., Spooner, R. A., Yoshimura, S., Lord, J. M., Barr, F. A. (2007). Specific Rab GTPase-activating proteins define the Shiga toxin and epidermal growth factor uptake pathways. J. Cell. Biol. 177, 1133–1143. doi: 10.1083/jcb.200612068
23. Fukuda, M. (2016). Multiple roles of VARP in endosomal trafficking: Rabs, retromer components and R-SNARE VAMP7 meet on VARP. Traffic 17, 709-719. doi: 10.1111/tra.12406.
24. Fukuda, M. (2011). TBC proteins: GAPs for mammalian small GTPase Rab? Biosci. Rep. 31, 159-168. doi: 10.1042/BSR20100112.
25. Gallo, L. I., Liao, Y., Ruiz, W. G., Clayton, D. R., Li, M., Liu, Y. J., et al. (2014). TBC1D9B functions as a GTPase-activating protein for Rab11a in polarized MDCK cells. Mol. Biol. Cell 25, 3779-3397. doi: 10.1091/mbc.E13-10-0604.
26. Goto-Ito, S., Morooka, N., Yamagata, A., Sato, Y., Sato, K., Fukai, S. (2019). Structural basis of guanine nucleotide exchange for Rab11 by SH3BP5. Life Sci. Alliance 2, pii: e201900297. doi: 10.26508/lsa.201900297.
27. Haas, A. K., Fuchs, E., Kopajtich, R., Barr, F. A. (2005). A GTPase-activating protein controls Rab5 function in endocytic trafficking. Nat. Cell Biol. 7, 887-893. doi: 10.1038/ncb1290.
28. Hadano, S., Otomo, A., Suzuki-Utsunomiya, K., Kunita, R., Yanagisawa, Y., Showguchi-Miyata, J., et al. (2004). ALS2CL, the novel protein highly homologous to the carboxy-terminal half of ALS2, binds to Rab5 and modulates endosome dynamics. FEBS Lett. 575, 64-70. doi: 10.1016/j.febslet.2004.07.092.
29. Hama, H., Tall, G. G., Horazdovsky, B. F. (1999). Vps9p is a guanine nucleotide exchange factor involved in vesicle-mediated vacuolar protein transport. J. Biol. Chem. 274, 15284-15291.

30. Hattula, K., Furuhjelm, J., Arffman, A., Peränen, J. (2002). A Rab8-specific GDP/GTP exchange factor is involved in actin remodeling and polarized membrane transport. Mol. Biol. Cell 13, 3268-3280. doi: 10.1091/mbc.e02-03-0143
31. Hiroi, T., Someya, A., Thompson, W., Moss, J., Vaughan, M. (2006). GEP100/BRAG2: activator of ADP-ribosylation factor 6 for regulation of cell adhesion and actin cytoskeleton via E-cadherin and alpha-catenin. Proc. Natl. Acad. Sci USA 103, 10672–10677. doi: 10.1073/pnas.0604091103.
32. Hokanson, D. E., Bretscher, A. P. (2012). EPI64 interacts with Slp1/JFC1 to coordinate Rab8a and Arf6 membrane trafficking. Mol. Biol. Cell. 23, 701-715. doi: 10.1091/mbc.E11-06-0521.
33. Homma, Y., Fukuda, M. (2016). Rabin8 regulates neurite outgrowth in both GEF activity-dependent and -independent manners. Mol. Biol. Cell. 27, 2107-1218. doi: 10.1091/mbc.E16-02-0091.
34. Horiuchi, H., Lippé, R., McBride, H. M., Rubino, M., Woodman, P., Stenmark, H., et al. (1997). A novel Rab5 GDP/GTP exchange factor complexed to Rabaptin-5 links nucleotide exchange to effector recruitment and function. Cell 90, 1149-1159. doi: 10.1016/s0092-8674(00)80380-3.
35. Hsu, C., Morohashi, Y., Yoshimura, S., Manrique-Hoyos, N., Jung, S., Lauterbach, M. A., et al. (2010). Regulation of exosome secretion by Rab35 and its GTPase-activating proteins TBC1D10A-C. J. Cell Biol. 189, 223-32. doi: 10.1083/jcb.200911018.
36. Hunker, C. M., Galvis, A., Kruk, I., Giambini, H., Veisaga, M. L., Barbieri, M. A. (2006). Rab5-activating protein 6, a novel endosomal protein with a role in endocytosis. Biochem. Biophys. Res. Commun. 340, 967-975. doi: 10.1016/j.bbrc.2005.12.099.
37. Ioannou, M. S., Bell, E. S., Girard, M., Chaineau, M., Hamlin, J. N., Daubaras, M., et al. (2015). DENND2B activates Rab13 at the leading edge of migrating cells and promotes metastatic behavior. J. Cell Biol. 208, 629–648. doi: 10.1083/jcb.201407068.
38. Ishida, M., Oguchi, M. E., Fukuda, M. (2016). Multiple types of guanine nucleotide exchange factors (GEFs) for Rab small GTPases. Cell Struct. Funct. 41, 61-79. doi: 10.1247/csf.16008.
39. Itzen, A., Pylypenko, O., Goody, R. S., Alexandrov, K., Rak, A. (2006). Nucleotide exchange via local protein unfolding–structure of Rab8 in complex with MSS4. EMBO J. 25, 1445-1455. doi: 10.1038/ sj.emboj.7601044.
40. Jackson, T. R., Brown, F. D., Nie, Z., Miura, K., Foroni, L., Sun, J., et al. (2000). ACAPs are arf6 GTPase-activating proteins that function in the cell periphery. J. Cell Biol. 151, 627-638. doi: 10.1083/jcb.151.3.627.
41. Jenkins, M. L., Margaria, J. P., Stariha, J. T. B., Hoffmann, R. M., McPhail, J. A., Hamelin, D. J., et al. (2018). Structural determinants of Rab11 activation by the guanine nucleotide exchange factor SH3BP5. Nat. Commun. 9, 3772. doi: 10.1038/s41467-018-06196-z.
42. Kajiho, H., Fukushima, S., Kontani, K., Katada, T. (2012). RINL, guanine nucleotide exchange factor Rab5-subfamily, is involved in the EphA8-degradation pathway with odin. PLoS One 7, e30575. doi: 10.1371/journal.pone.0030575.
43. Kajiho, H., Saito, K., Tsujita, K., Kontani, K., Araki, Y., Kurosu, H., Katada, T. (2003). RIN3: a novel Rab5 GEF interacting with amphiphysin II involved in the early endocytic pathway. J. Cell Sci. 116, 4159-4168. doi: 10.1242/jcs.00718.
44. Kajiho, H., Sakurai, K., Minoda, T., Yoshikawa, M., Nakagawa, S., Fukushima, S., et al. (2011). Characterization of RIN3 as a guanine nucleotide exchange factor for the Rab5 subfamily GTPase Rab31. J. Biol. Chem. 286, 24364-24373. doi: 10.1074/jbc.M110.172445.
45. Kanno, E., Ishibashi, K., Kobayashi, H., Matsui, T., Ohbayashi, N., Fukuda, M. (2010). Comprehensive screening for novel rab-binding proteins by GST pull-down assay using 60 different mammalian Rabs. Traffic 11, 491–507. doi: 10.1111/j.1600-0854.2010.01038.x.
46. Kitano, M., Nakaya, M., Nakamura, T., Nagata, S., Matsuda, M. (2008). Imaging of Rab5 activity identifies essential regulators for phagosome maturation. Nature 453, 241-245. doi: 10.1038/nature06857.
47. Klinkert, K., Echard, A. (2016). Rab35 GTPase: a central regulator of phosphoinositides and F-actin in endocytic recycling and beyond. Traffic 17, 1063-1077. doi: 10.1111/tra.12422.
48. Knödler, A., Feng, S., Zhang, J., Zhang, X., Das, A., Peranen, J., Guo, W. (2010). Coordination of Rab8 and Rab11 in primary ciliogenesis. Proc. Natl. Acad. Sci. USA 107, 6346-6351. doi: 10.1073/pnas.1002401107.

49. Linford, A., Yoshimura, S., Nunes Bastos, R., Langemeyer, L., Gerondopoulos, A., Rigden, D. J., Barr, F. A. (2012). Rab14 and its exchange factor FAM116 link endocytic recycling and adherens junction stability in migrating cells. Dev. Cell 22, 952-966. doi: 10.1016/j.devcel.2012.04.010.
50. Lodhi, I. J., Chiang, S. H., Chang, L., Vollenweider, D., Watson, R. T., Inoue, M., et al. (2007). Gapex-5, a Rab31 guanine nucleotide exchange factor that regulates Glut4 trafficking in adipocytes. Cell Metab. 5, 59–72. doi: 10.1016/j.cmet.2006.12.006.
51. Macia, E., Chabre, M., Franco, M. (2001). Specificities for the small G proteins ARF1 and ARF6 of the guanine nucleotide exchange factors ARNO and EFA6. J. Biol Chem. 276, 24925-24930. doi: 10.1074/jbc.M103284200.
52. Macia, E., Partisani, M., Paleotti, O., Luton, F., Franco, M. (2012). Arf6 negatively controls the rapid recycling of the β2 adrenergic receptor. J. Cell Sci. 125, 4026-4035. doi: 10.1242/jcs.102343.
53. Marat, A. L., Dokainish, H., McPherson, P.S. (2011). DENN domain proteins: regulators of Rab GTPases. J. Biol. Chem. 286, 13791–13800. doi: 10.1074/jbc.R110.217067.
54. Marat, A. L., McPherson, P. S. (2010). The connecdenn family, Rab35 guanine nucleotide exchange factors interfacing with the clathrin machinery. J. Biol. Chem. 285, 10627–10637. doi: 10.1074/jbc.M109.050930.
55. Matsuya, S., Sakagami, H., Tohgo, A., Owada, Y., Shin, H. W., Takeshima, H., et al. (2005). Cellular and subcellular localization of EFA6C, a third member of the EFA6 family, in adult mouse Purkinje cells. J. Neurochem. 93, 74–85. doi: 10.1111/j.1471-4159.2005.03072.x.
56. Miserey-Lenkei, S., Couëdel-Courteille, A., Del Nery, E., Bardin, S., Piel, M., Racine, V., et al. (2006). A role for the Rab6A' GTPase in the inactivation of the Mad2-spindle checkpoint. EMBO J. 25, 278-289. doi: 10.1038/sj.emboj.7600929.
57. Müller, M. P., Goody, R. S. (2018). Molecular control of Rab activity by GEFs, GAPs and GDI. Small GTPases 9, 5-21. doi: 10.1080/21541248.2016.1276999.
58. Murga-Zamalloa, C. A., Atkins, S. J., Peranen, J., Swaroop, A., Khanna, H. (2010). Interaction of retinitis pigmentosa GTPase regulator (RPGR) with RAB8A GTPase: implications for cilia dysfunction and photoreceptor degeneration. Hum. Mol. Genet. 19, 3591-3598. doi: 10.1093/hmg/ddq275.
59. Nishikimi, A., Ishihara, S., Ozawa, M., Etoh, K., Fukuda, M., Kinashi, T., Katagiri, K. (2014). Rab13 acts downstream of the kinase Mst1 to deliver the integrin LFA-1 to the cell surface for lymphocyte trafficking. Sci. Signal. 7, ra72. doi: 10.1126/scisignal.2005199.
60. Nookala, R. K., Langemeyer, L., Pacitto, A., Ochoa-Montaño, B., Donaldson, J. C., Blaszczyk, B. K., Chirgadze, D. Y., et al. (2012). Crystal structure of folliculin reveals a hidDENN function in genetically inherited renal cancer. Open Biol. 2, 120071. doi: 10.1098/rsob.120071.
61. Nottingham, R. M., Pusapati, G. V., Ganley, I. G., Barr, F. A., Lambright, D. G., Pfeffer, S. R. (2012). RUTBC2 protein, a Rab9A effector and GTPase-activating protein for Rab36. J. Biol. Chem. 287, 22740-22748. doi: 10.1074/jbc.M112.362558.
62. Padovani, D., Folly-Klan, M., Labarde, A., Boulakirba, S., Campanacci, V., Franco, M., et al. (2014). EFA6 controls Arf1 and Arf6 activation through a negative feedback loop. Proc. Natl. Acad. Sci. USA. 111, 12378-12383. doi: 10.1073/pnas.1409832111.
63. Pusapati, G. V., Luchetti, G., Pfeffer, S. R. (2012). Ric1-Rgp1 complex is a guanine nucleotide exchange factor for the late Golgi Rab6A GTPase and an effector of the medial Golgi Rab33B GTPase. J. Biol. Chem. 287, 42129-42137. doi: 10.1074/jbc.M112.414565.
64. Pylypenko, O., Hammich, H., Yu, I. M., Houdusse, A. (2018). Rab GTPases and their interacting protein partners: Structural insights into Rab functional diversity. Small GTPases 9, 22-48. doi: 10.1080/21541248.2017.1336191.
65. Qu, F., Lorenzo, D. N., King, S. J., Brooks, R., Bear, J. E., Bennett, V. (2016). Ankyrin-B is a PI3P effector that promotes polarized α5β1-integrin recycling via recruiting RabGAP1L to early endosomes. Elife. 5. pii, e20417. doi: 10.7554/eLife.20417.
66. Rahajeng, J., Giridharan, S. S., Cai, B., Naslavsky, N., Caplan, S. (2012). MICAL-L1 is a tubular endosomal membrane hub that connects Rab35 and Arf6 with Rab8a. Traffic 13, 82–93. doi: 10.1111/j.1600-0854.2011.01294.x.

67. Roach, W. G., Chavez, J. A., Mîinea, C. P., Lienhard, G. E. (2007). Substrate specificity and effect on GLUT4 translocation of the Rab GTPase-activating protein Tbc1d1. Biochem. J. 403, 353-358. doi: 10.1042/BJ20061798.
68. Saegusa, S., Fukaya, M., Kakegawa, W., Tanaka, M., Katsumata, O., Sugawara, T. (2019). Mice lacking EFA6C/Psd2, a guanine nucleotide exchange factor for Arf6, exhibit lower Purkinje cell synaptic density but normal cerebellar motor functions. PLoS One 14, e0216960. doi: 10.1371/journal.pone.0216960.
69. Saito, K., Murai, J., Kajiho, H., Kontani, K., Kurosu, H., Katada, T. (2002). A novel binding protein composed of homophilic tetramer exhibits unique properties for the small GTPase Rab5. J. Biol. Chem. 277, 3412-3418. doi: 10.1074/jbc.M106276200.
70. Sakagami, H., Suzuki, H., Kamata, A., Owada, Y., Fukunaga, K., Mayanagi, H., et al. (2006). Distinct spatiotemporal expression of EFA6D, a guanine nucleotide exchange factor for ARF6, among the EFA6 family in mouse brain. Brain Res. 1093, 1–11. doi: 10.1016/j.brainres.2006.02.058.
71. Sakaguchi, A., Sato, M., Sato, K., Gengyo-Ando, K., Yorimitsu, T., Nakai, J., et al. (2015). REI-1 is a guanine nucleotide exchange factor regulating RAB-11 localization and function in C. elegans embryos. Dev. Cell 35, 211-221. doi: 10.1016/j.devcel.2015.09.013
72. Sato, M., Sato, K., Fonarev, P., Huang, C. J., Liou, W., Grant, B. D. (2005). Caenorhabditis elegans RME-6 is a novel regulator of RAB-5 at the clathrin-coated pit. Nat. Cell Biol. 7, 559-569. doi: 10.1038/ncb1261.
73. Smythe, E. (2015). Role of the rab5 guanine nucleotide exchange factor, Rme-6, in the regulation of clathrin-coated vesicle uncoating. Methods Mol. Biol. 1298, 283-293. doi: 10.1007/978-1-4939-2569-8_24.
74. Someya, A., Sata, M., Takeda, K., Pacheco-Rodriguez, G., Ferrans, V. J., Moss, J., et al. (2001). ARF-GEP(100), a guanine nucleotide-exchange protein for ADP-ribosylation factor 6. Proc. Natl. Acad. Sci. USA 98, 2413–2418. doi: 10.1073/pnas.051634798.
75. Sun, Y., Bilan, P. J., Liu, Z., Klip, A. (2010). Rab8A and Rab13 are activated by insulin and regulate GLUT4 translocation in muscle cells. Proc. Natl. Acad. Sci. USA 107, 19909-19914. doi: 10.1073/pnas.1009523107.
76. Tall, G. G., Barbieri, M. A., Stahl, P. D., Horazdovsky, B. F. (2001). Ras-activated endocytosis is mediated by the Rab5 guanine nucleotide exchange activity of RIN1. Dev. Cell 1, 73-82. doi: 10.1016/s1534-5807(01)00008-9.
77. Topp, J. D., Gray, N. W., Gerard, R. D., Horazdovsky, B. F. (2004). Alsin is a Rab5 and Rac1 guanine nucleotide exchange factor. J. Biol. Chem. 279, 24612-24623.
78. Vaibhava, V., Nagabhushana, A., Chalasani, M. L., Sudhakar, C., Kumari, A., Swarup, G. (2012). Optineurin mediates a negative regulation of Rab8 by the GTPase-activating protein TBC1D17. J. Cell Sci. 125, 5026-5039. doi: 10.1242/jcs.102327.
79. Wang, J., Deretic, D. (2015). The Arf and Rab11 effector FIP3 acts synergistically with ASAP1 to direct Rabin8 in ciliary receptor targeting. J. Cell Sci. 128, 1375-1385. doi: 10.1242/jcs.162925.
80. Westlake, C. J., Junutula, J. R., Simon, G. C., Pilli, M., Prekeris, R., Scheller, R. H., et al. (2007). Identification of Rab11 as a small GTPase binding protein for the Evi5 oncogene. Proc. Natl. Acad. Sci. USA 104, 1236–1241. doi: 10.1073/pnas.0610500104.
81. Woller, B., Luiskandl, S., Popovic, M., Prieler, B. E., Ikonge, G., Mutzl, M., et al. (2011). Rin-like, a novel regulator of endocytosis, acts as guanine nucleotide exchange factor for Rab5a and Rab22. Biochim. Biophys. Acta 1813, 1198-1210. doi: 10.1016/j.bbamcr.2011.03.005.
82. Yoshimura, S., Gerondopoulos, A., Linford, A., Rigden, D. J., Barr, F. A. (2010). Family-wide characterization of the DENN domain Rab GDP-GTP exchange factors. J. Cell Biol. 191, 367-381. doi: 10.1083/jcb.201008051.
83. Zhang, X.-M., Walsh, B., Mitchell, C. A., Rowe, T. (2005). TBC domain family, member 15 is a novel mammalian Rab GTPase-activating protein with substrate preference for Rab7. Biochem. Biophys. Res. Commun. 335, 154–161. doi: 10.1016/j.bbrc.2005.07.070.
84. Zheng, J., Duan, B., Sun, S., Cui, J., Du, J., Zhang, Y. (2017). Folliculin Interacts with Rab35 to Regulate EGF-Induced EGFR Degradation. Front. Pharmacol. 8, 688. doi: 10.3389/fphar.2017.00688. eCollection 2017.
