# Supplementary material for: Cytomegalovirus Generates Assembly Compartment in the Early Phase of Infection by Perturbation of Host-Cell Factors Recruitment at the Early Endosome/Endosomal Recycling Compartment/Trans-Golgi Interface
Source: Front Cell Dev Biol. 2020 Sep 11;8:563607. doi: 10.3389/fcell.2020.563607 (PMC7516400; doi:10.3389/fcell.2020.563607)
Supplement: Supplementary file 4 [file Data_Sheet_4.PDF]

Supplementary Material

Supplementary figures S1-S6

Transcriptome validation

Quality control of raw sequencing reads obtained from the core sequencing facility was performed using FastQC (https://www.bioinformatics.babraham.ac.uk/projects/fastqc/). In addition, FastQ Screen (Wingett and Andrews, 2018) and Bowtie 2 (Langmead and Salzberg, 2012) were used to screen all libraries against an in-home built database containing Bowtie 2 indices of the most common biological contaminants of laboratory mice (Pritchett-Corning et al., 2009) and cell cultures (Mahmood and Ali, 2017), as well as indices of technical contaminants, such as vectors, adapters and rRNA sequences (Cochrane and Galperin, 2010). Following pre-mapping quality control, STAR aligner v.2.7.3a (Dobin et al., 2015; Dobin and Gingeras, 2015; Dobin and Gingeras, 2016) was used to map raw sequencing reads to a custom genome generated by concatenating the GENCODE nucleotide sequence of the mouse GRCm38.p6 primary genome assembly (release M23) (Frankish et al., 2019), and the wild-type MCMV genome sequence, strain Smith (PubMed accession no. NC_004065.1). Obtained coordinate sorted alignment files in .bam format were indexed using SAMTools v1.9 (Li et al., 2009). To identify potential errors, outliers or other issues that could jeopardize differential expression analysis, visual inspection of mapping results and comprehensive post-mapping quality controls were then performed in IGV v2.7.2 (Robinson et al., 2017; Robinson et al., 2011; Thorvaldsdottir et al., 2013) and QoRTs v1.3.6 (Hartley and Mullikin, 2015). Outputs from all supported tools were systematized using MultiQC v1.7 (Ewels et al., 2016). Summarization of reads mapping to exons of mouse genes was performed using featureCounts v2.0 (Liao et al., 2014). Principal component analysis of the samples, gene-expression estimates, normalization of the expression data and differential expression analysis was performed using DESeq2 v1.26.0 (Love et al., 2014) in R programming environment v3.6.1 (RCoreTeam, 2019) using RStudio v.1.2.5019 (RStudioTeam, 2015) under Canonical Ubuntu v18.04 open-source operating system.

Supplementary references:

1. Wingett SW, Andrews S (2018). FastQ Screen: A tool for multi-genome mapping and quality control. F1000Res. **7**, 1338.
3. Langmead B, Salzberg SL (2012). Fast gapped-read alignment with Bowtie 2. Nat. Methods **9**, 357-359.
4. Pritchett-Corning KR, Cosentino J, Clifford CB (2009). Contemporary prevalence of infectious agents in laboratory mice and rats. Lab. Anim. **43**, 165-173.
5. Mahmood A, Ali S (2017). Microbial and viral contamination of animal and stem cell cultures: common contaminants, detection and elimination. J. Stem Cell Res. Therap. **2**, 149-155.
6. Cochrane GR, Galperin MY (2010). The 2010 Nucleic Acids Research Database Issue and online Database Collection: a community of data resources. Nucleic Acids Res. **38**, D1-4.
7. Dobin A, Davis CA, Schlesinger F, Drenkow J, Zaleski C, Jha S, et al. (2013). STAR: ultrafast universal RNA-seq aligner. Bioinformatics **29**, 15-21.
8. Dobin A, Gingeras TR (2015). Mapping RNA-seq Reads with STAR. Curr. Protoc. Bioinformatics **51**, 11 14 11-19.

9. Dobin A, Gingeras TR (2016). Optimizing RNA-Seq Mapping with STAR. Methods Mol. Biol. **1415**, 245-262.
10. Frankish A, Diekhans M, Ferreira AM, Johnson R, Jungreis I, Loveland J, et al. (2019). GENCODE reference annotation for the human and mouse genomes. Nucleic Acids Res. **47**, D766-D773
11. Li H, Handsaker B, Wysoker A, Fennell T, Ruan J, Homer N, et al. (2009). The Sequence Alignment/ Map format and SAMtools. Bioinformatics **25**, 2078-2079.
12. Robinson JT, Thorvaldsdottir H, Wenger AM, Zehir A, Mesirov JP (2017). Variant Review with the Integrative Genomics Viewer. Cancer Res. **77**, e31-e34.
13. Robinson JT, Thorvaldsdottir H, Winckler W, Guttman M, Lander ES, Getz G, Mesirov JP (2011). Integrative genomics viewer. Nat. Biotechnol. **29**, 24-26.
14. Thorvaldsdottir H, Robinson JT, Mesirov JP (2013). Integrative Genomics Viewer (IGV): high-performance genomics data visualization and exploration. Brief Bioinform **14**, 178-192.
15. Hartley SW, Mullikin JC (2015). QoRTs: a comprehensive toolset for quality control and data processing of RNA-Seq experiments. BMC Bioinformatics **16**, 224.
16. Ewels P, Magnusson M, Lundin S, Kaller M (2016). MultiQC: summarize analysis results for multiple tools and samples in a single report. Bioinformatics **32**, 3047-3048.
17. Liao Y, Smyth GK, Shi W (2014). featureCounts: an efficient general purpose program for assigning sequence reads to genomic features. Bioinformatics **30**, 923-930.
18. Love MI, Huber W, Anders S (2014). Moderated estimation of fold change and dispersion for RNA-seq data with DESeq2. Genome Biol**.** **15**, 550.
19. RCoreTeam (2019). R: A language and environment for statistical computing. *R Foundation for Statistical Computing, Vienna, Austria.*
20. RStudioTeam (2015). RStudio: Integrated Development for R. RStudio, Inc., Boston, MA URL.

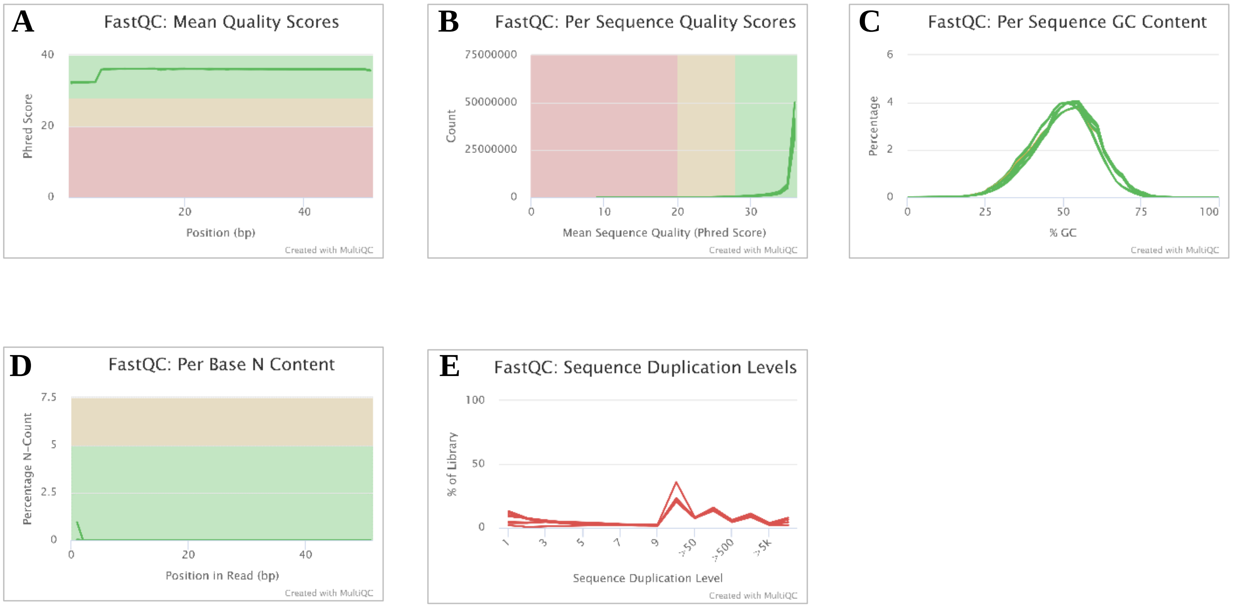

Figure S1. **An overview of the pre-mapping quality control results obtained with FastQC.** A total of 43.0M to 68.5M reads were obtained from the sequencing libraries of the mock-infected and MCMV infected DC2.4 cells. All reads in all the samples had equal size (51 bp) and; (**A**) a mean quality score higher than 30 (Q>30) along their lengths and, consistent with this observation (**B**) a large number of reads had very high sequence quality scores (distribution of average read quality was narrow at the upper-quality range). Base composition along the read length was consistent with a typical RNA-Seq experiment (data not shown), while the (**C**) distribution of reads GC content was consistent with the experimental setup and theoretical distributions. Additionally, (**D**) successful base call frequencies were very high for all the samples, and the levels of overrepresented sequences (data not shown) and (**E**) sequence duplication levels were consistent with an RNA sequencing experiment. Finally, not a single sample had an adapter contamination level higher than 0.1% (not shown).

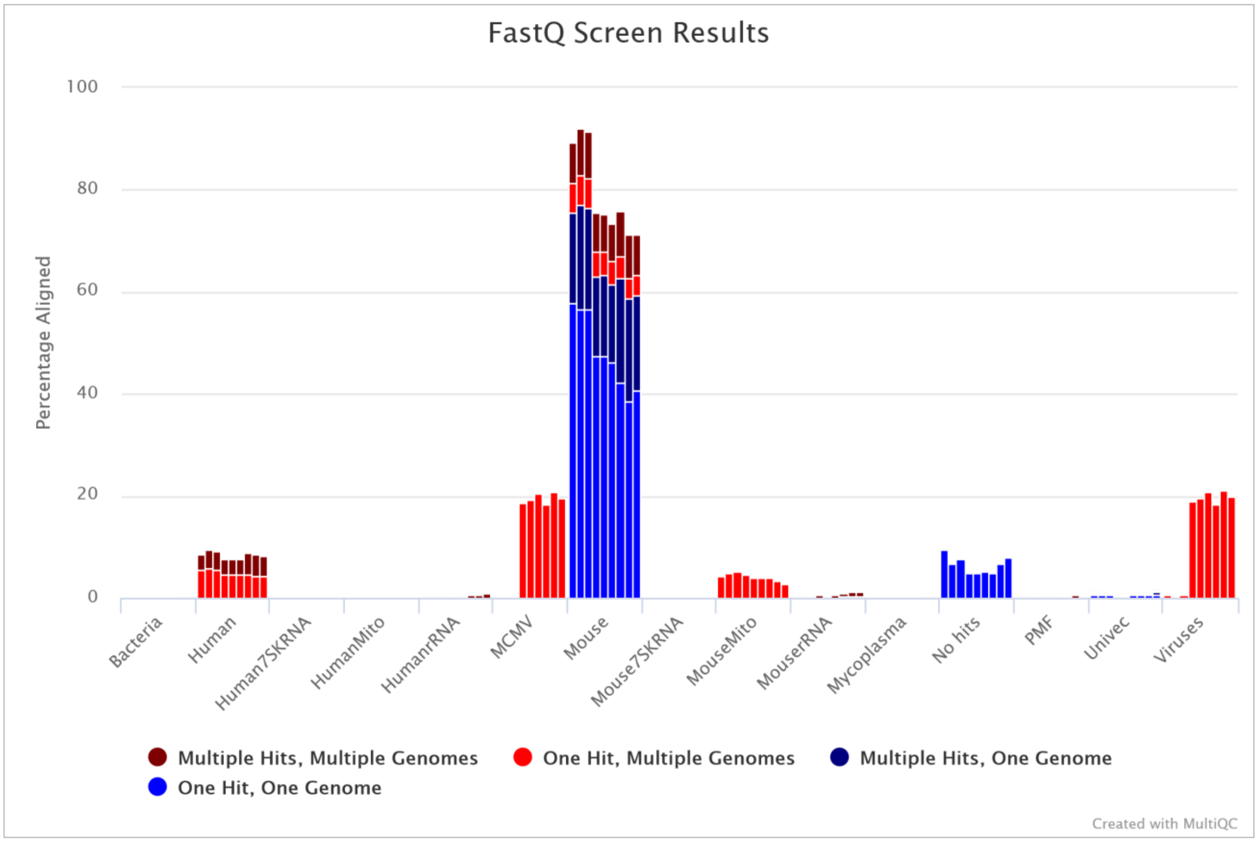

Figure S2. **Screening of sequencing libraries for the most common biological and technical contaminants of cell cultures arising in a typical mouse RNA sequencing experiment.** A total of 9 sequencing libraries (3 for each triplicate) were screened for contaminants using FastQ Screen and Bowtie 2 aligner, as described in Materials and methods. As expected, a substantial proportion of reads in each sample mapped exclusively to the mouse genome and, in six MCMV infected samples, to MCMV genome sequence as well. A small percentage of reads mapped to both human and mouse genomes, which is not unexpected due to the relatively close evolutionary relationship and homology between human and mouse genes. Importantly, close to none, or none of the reads mapped to genomic sequences of bacteria, mycoplasmas, protozoans, metazoans, and fungi, as well as to sequences of vectors and adapters from the UniVec database. In short, the composition of the sequencing libraries was in accordance with expectations and excluded the possibility of sample contamination with undesired or wrong target nucleic acid molecules. **Bacteria:** sequences of the *Brevibacillus brevis*, *Bacillus cereus*, *Bacillus coagulans*, *Bordetella bronchiseptica*, *Flexibacter litoralis*, *Fusobacterium hwasookii*, *Citrobacter rodentium*, *Corynebacterium kutscheri*, *Enterococcus casseliflavus*, *Escherichia* coli, *Enterococcus malodoratus*, *Helicobacter bilis*, *Helicobacter hepaticus*, *Klebsiella oxytoca*, *Klebsiella pneumoniae*, *Pasteurella multocida*, *Pasteurella pneumotropica*, *Salmonella enterica* subsp. *enterica serovar Typhi*, *Staphylococcus epidermidis*, *Staphylococcus felis*, *Staphylococcus aureus*, *Streptobacillus moniliformi*, *Streptococcus agalactiae*, *Streptococcus canis*, and *Streptococcus pneumoniae*; **Human:** human genome sequence; **Human7SKRNA:** human 7SK RNA sequence; **HumanMito:** human mitochondrial genome sequence; **Human rRNA:** human rRNA sequences; **MCMV:** wild-type mouse cytomegalovirus genome sequence; **Mouse:** mouse genome sequence; **Mouse7SKRNA:** mouse 7SK RNA sequence; **MouseMito:** mouse mitochondrial genome sequence; **Mouse rRNA:** mosue rRNA sequences; Mycoplasma: sequences of *Mycoplasma arginini*,

Mycoplasma fermentans, *Mycoplasma hominis*, *Mycoplasma hyorhinis*, *Mycoplasma orale*, and *Mycoplasma pulmonis*; **PMF:** sequences of protozoans, metazoans and fungi such as *Aspergillus flavus*, *Aspergillus niger*, *Botrytis cinerea*, *Candida albicans*, *Encephalitozoon cuniculi*, *Entamoeba histolytica*, *Giardia lamblia*, *Penicillium chrysogenum*, *Paecilomyces hepialid*, *Saccharomyces cerevisiae*, *Spironucleus salmonicida*, *Syphacia muris* and *Trichomonas vaginalis*. **UniVec:** 3039 sequences/6093 segments of vectors, adapters, primers and linkers commonly used in cloning experiments; **Viruses:** genome sequences of *Ectromelia virus*, *Hantaan virus*, *Hepatitis B virus*, *Human herpesvirus 5 strain Towne*, *Hepatitis C virus genotype 1*, *Hepatitis delta virus*, *Hepatitis E virus*, *Human immunodeficiency virus 1*, *Human immunodeficiency virus 2*, *Human papillomavirus type 18*, *Human T-lymphotropic virus 1*, *Human T-lymphotropic virus 2*, *Polyomavirus sp. isolate poly-CA1*, *Lymphocytic choriomeningitis virus*, *Mammalian orthoreovirus*, *Murid herpesvirus 1*, *Murine hepatitis virus strain S*, *Minute virus of mice*, *Murine norovirus GV/CR18/2005/DEU*, *Mouse parvovirus 1e*, *Mouse parvovirus 2*, *Murine polyomavirus strain BG*, *Murine adenovirus A*, *Murine adenovirus 2*, *Murine adenovirus 3*, *Murine pneumotropic virus isolate #6022*, *Murine type C retrovirus*, *Pneumonia virus of mice strain 15*, *Sendai virus*, *Simian virus 40*, *Theiler's encephalomyelitis virus*; **No hits:** reads that did not map to any of the genomes present in the FastQ Screen database.

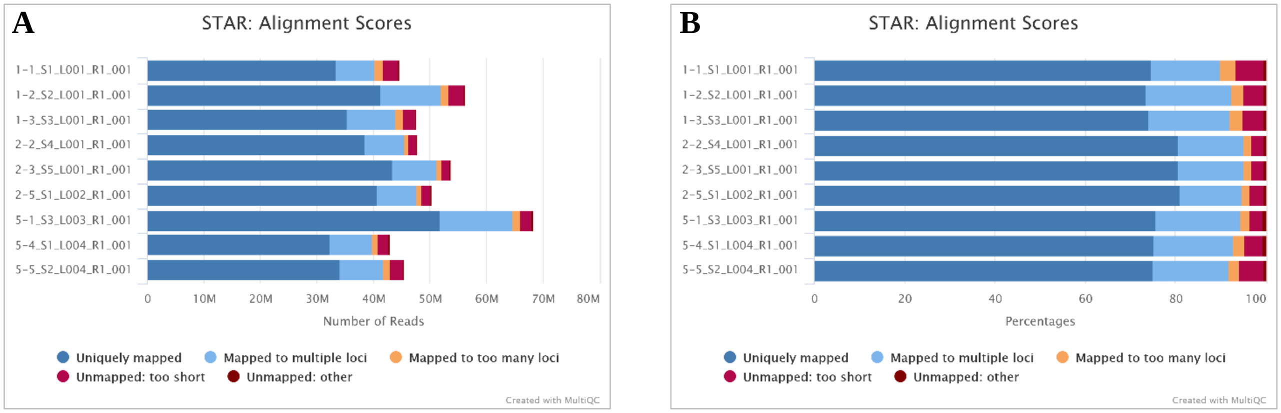

Figure S3. **Read mapping statistics.** (**A**) The absolute numbers of reads that mapped uniquely to mouse-MCMV genome sequences were, as expected, dependent on the depth to which each library was sequenced and varied from 32.3M-51.8M reads. However, consistent with pre-mapping quality control, (**B**) the proportions of sequencing reads that mapped exclusively to the mouse-MCMV genome sequences were uniform, high (73.5% - 81.0%), and in accordance with expectations for this type of experiment.

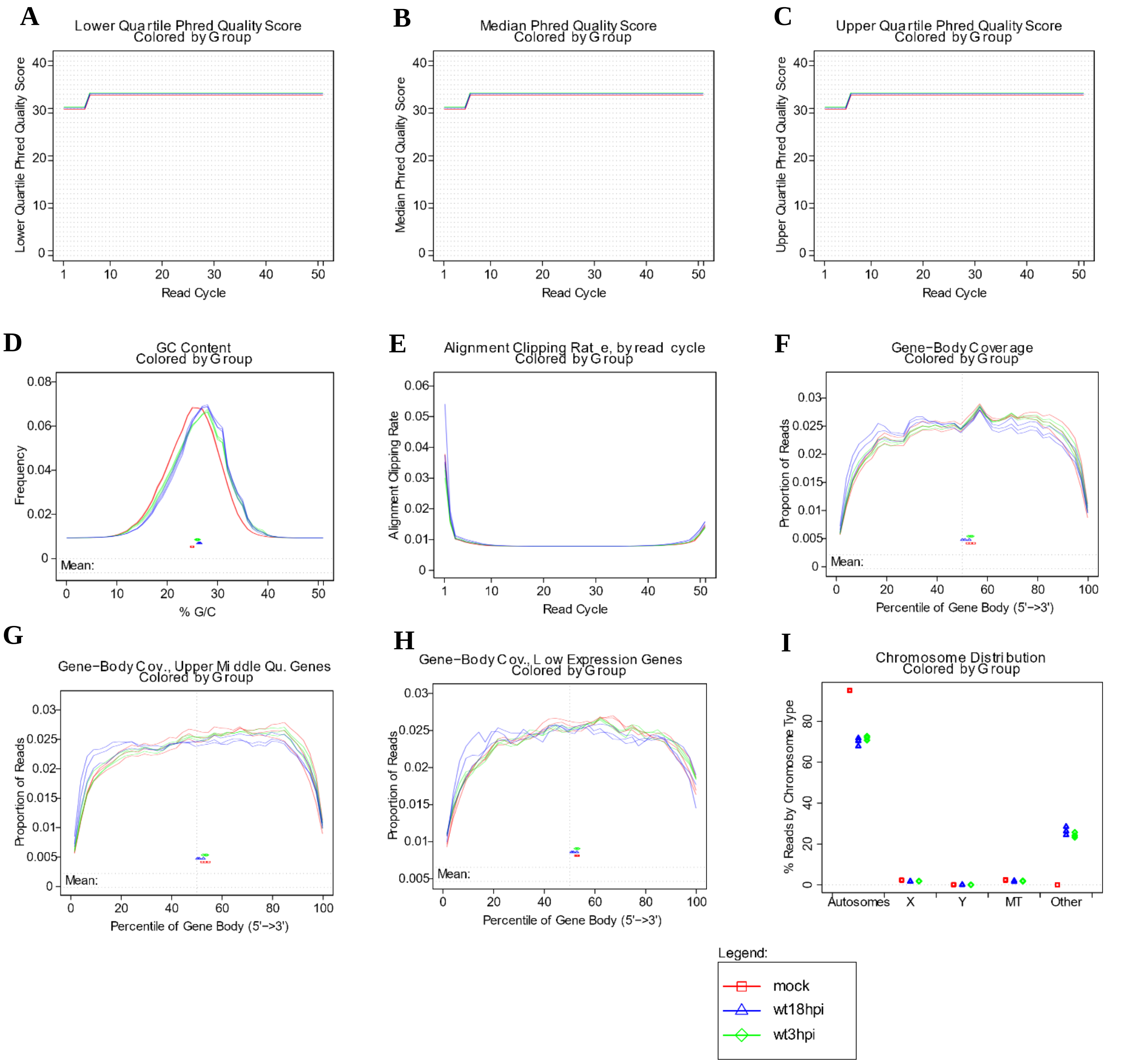

Figure S4. **Post-mapping quality control results.** To further establish the absence of potential errors, biases, and/or other issues in our RNASeq experiment, we have produced detailed post-mapping quality metrics on all samples used in this study using QoRTs. A selection of QC plots shown above demonstrated that (**A, B, C**) Lower, median and upper quartile Phred quality scores are high and consistent across all samples; (**D**) There were no apparent GC biases between samples as well as no visible differences in; (**E**) alignment clipping rates for different samples. In addition, (**F, G, H**) gene-body coverage for all, upper-middle quartile and low expression genes was consistent, and there were no apparent biases in read coverage at either 5' or 3'- ends of the transcripts in any of the samples. As expected, (**I**) distribution of mapped reads corresponded to the experimental setup, whereby a majority of reads mapped to mouse autosomes in both mock and wt-MCMV infected cells, with a significant proportion of reads mapping to MCMV genome in MCMV-infected samples. Additional QC plots produced by QoRTs (not shown) were either in complete agreement with data shown in Supplementary Figures 1-3 or failed to reveal any systematic biases in the RNASeq dataset.

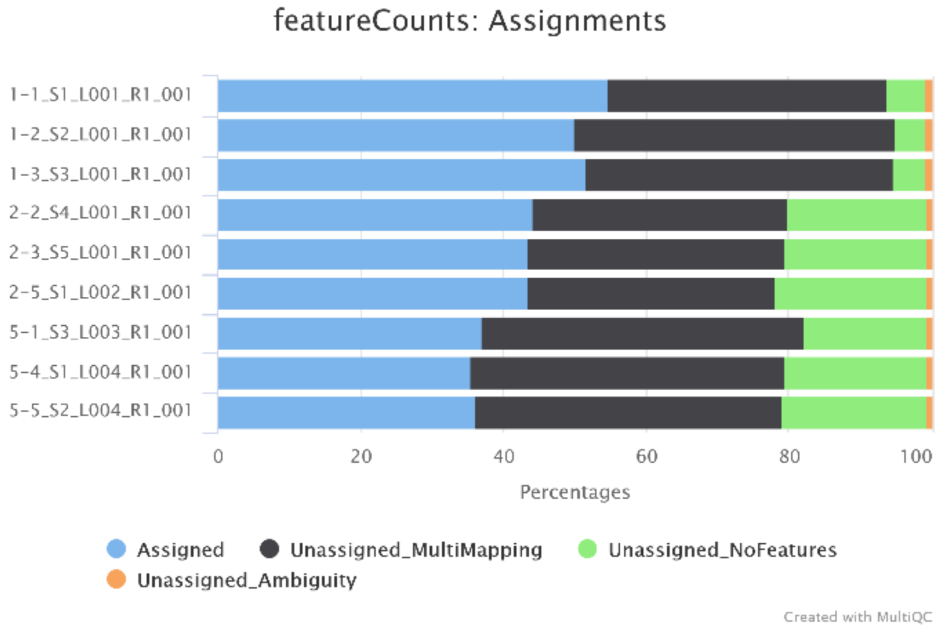

Figure S5. **Read summarization statistics.** Of all mapped reads, 35.5-54.7% mapped uniquely to mouse exons. As expected, the largest proportion of reads that mapped to the mouse genome was observed in mock-infected samples, since those sequencing libraries did not contain any MCMV transcripts. Furthermore, and consistent with time points post-infection chosen for RNA isolation and sequencing, smaller proportion of reads mapped uniquely to mouse genes in MCMV infected cells 3 hours post-infection, and even less in MCMV infected cells 18 hours post-infection. Only the numbers of uniquely mapped reads, as assigned by featureCounts, were used for differential gene expression analysis.

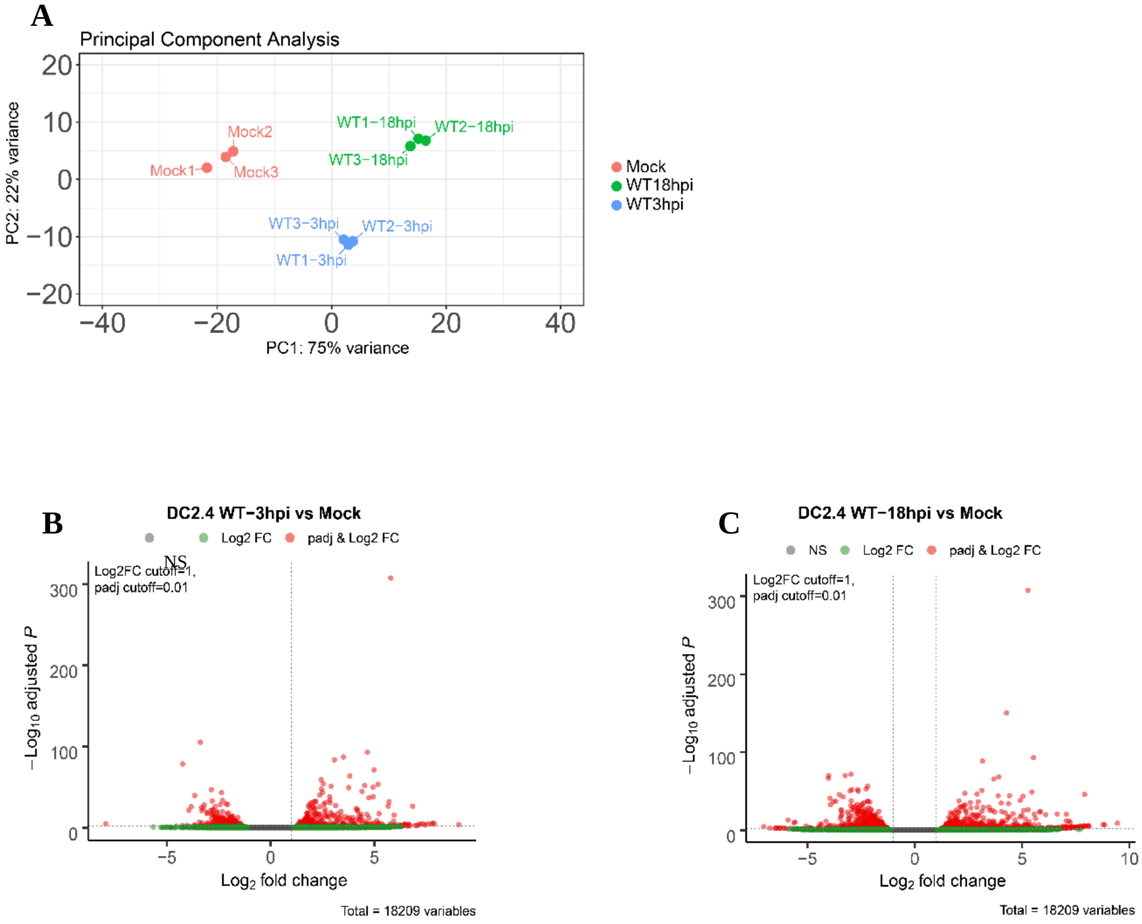

Figure S6. **PCA and overview of differentially expressed genes.** (**A**) Sample to sample distances were visualized by applying the PCA function of the DESeq2 package on vst normalized counts. As can be seen from the PCA plot, the similarities between samples are in full accordance with expectations from the experimental design. The differences in gene expression between (**B**) wt-MCMV infected cells at 3 hpi vs mock-infected cells; and (**C**) wt-MCMV infected cells at 18 hpi vs mock-infected cells were evaluated by running the differential expression pipeline of the DESeq2 package and visualized using the Enhanced Volcano package. Genes with false discovery rate threshold of padj<0.01 and log2FC threshold larger than 1 were considered to be significantly differentially expressed in MCMV-infected versus the mock-infected cells. Overall, at 3 hours-post-infection out of 519 DE genes, 282 were upregulated in MCMV-infected cells, while 237 were downregulated. At 18 hours post-infection, the number of significant DE genes increased almost twofold, to a total of 986 DE genes, 458 of which were upregulated and 528 of which were downregulated in MCMV infected cells.
