# Supplementary material for: Cytomegalovirus Generates Assembly Compartment in the Early Phase of Infection by Perturbation of Host-Cell Factors Recruitment at the Early Endosome/Endosomal Recycling Compartment/Trans-Golgi Interface
Source: Front Cell Dev Biol. 2020 Sep 11;8:563607. doi: 10.3389/fcell.2020.563607 (PMC7516400; doi:10.3389/fcell.2020.563607)
Supplement: Supplementary file 5 [file Data_Sheet_5.PDF]

Supplementary Material

Figure S7

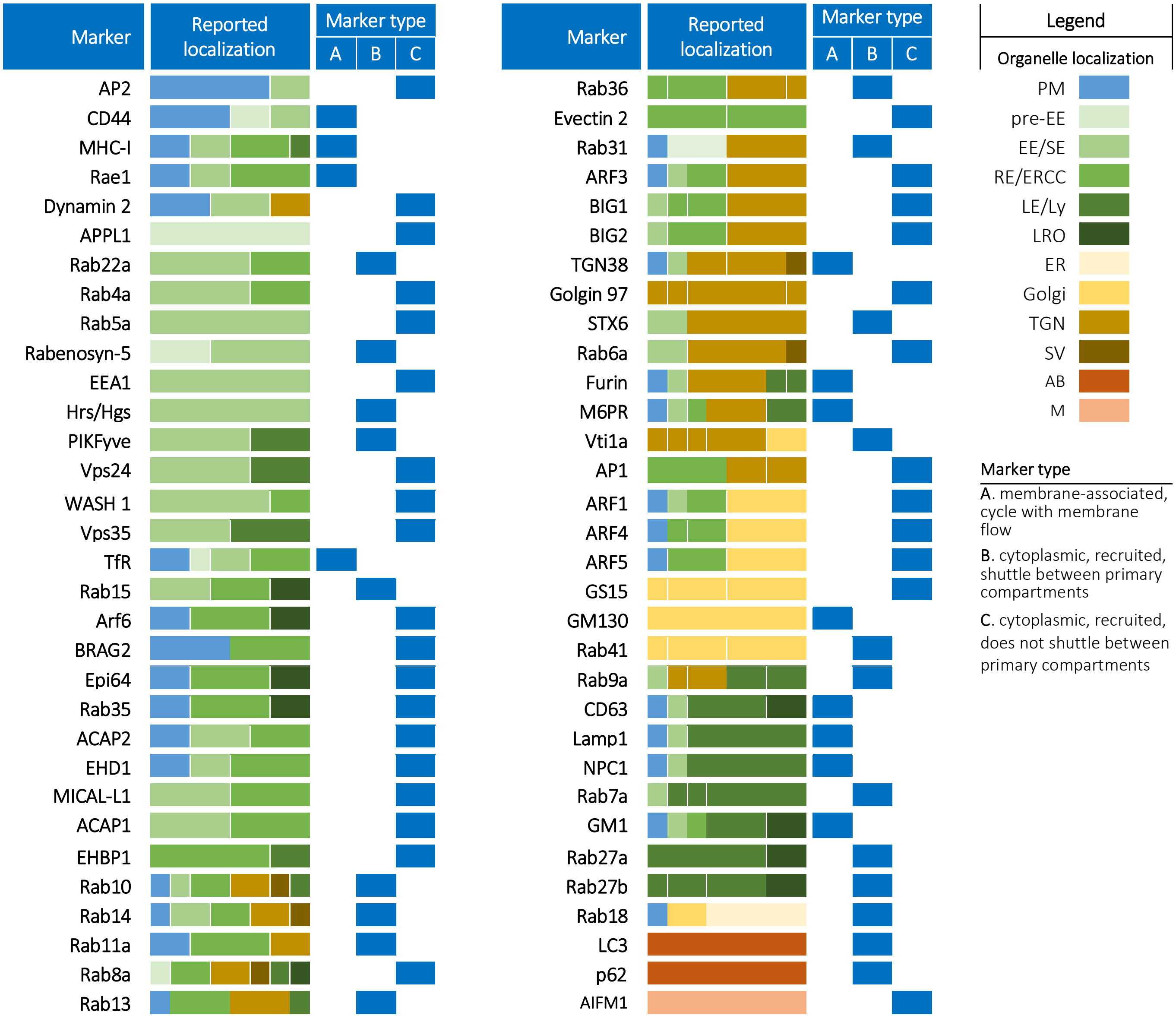

Figure S7. **Subcellular localization and types of markers of membranous organelles used in this study** (related to Fig. 1 and Fig. 9). Localizations reported in the literature are presented as an approximation of relative distribution. The detailed description of the markers is presented in Table S2. PM, plasma membrane; EE/SE, early/sorting endosome; RE/ERC, recycling endosomes/endosomal recycling compartment; LE/Ly, late endosome/lysosome; LRO, lysosome-related organelles; ER, endoplasmic reticulum; TGN, trans-Golgi network; SV, secretory vesicles; A, apoptotic bodies; M, mitochondria.
