# Supplementary material for: Cytomegalovirus Generates Assembly Compartment in the Early Phase of Infection by Perturbation of Host-Cell Factors Recruitment at the Early Endosome/Endosomal Recycling Compartment/Trans-Golgi Interface
Source: Front Cell Dev Biol. 2020 Sep 11;8:563607. doi: 10.3389/fcell.2020.563607 (PMC7516400; doi:10.3389/fcell.2020.563607)
Supplement: Supplementary file 6 [file Data_Sheet_6.PDF]

Supplementary figures S8-S17

Immunofluorescence experiments

Figure S8

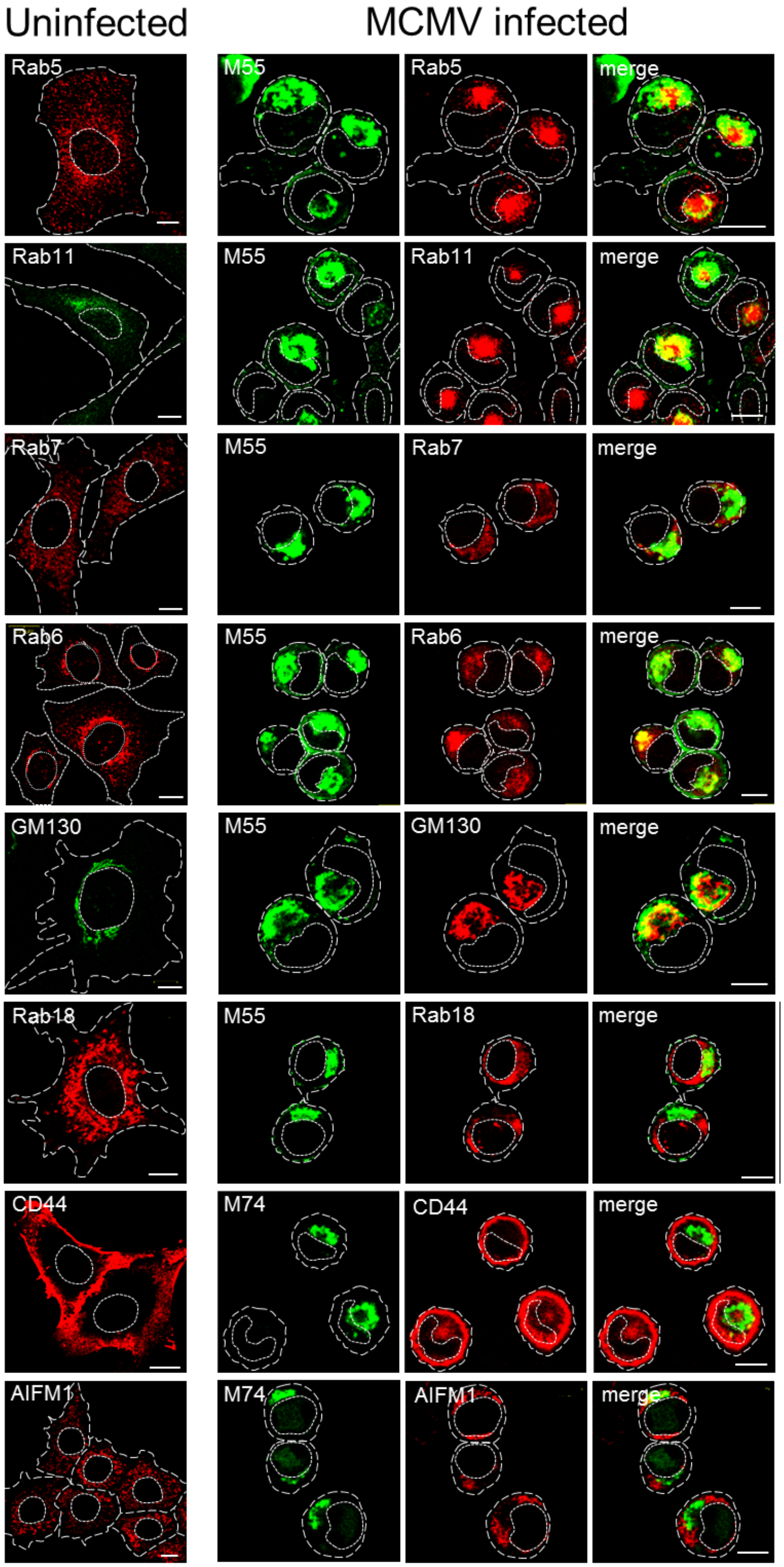
Uninfected
MCMV infected
Rab5
M55
Rab5
merge
Rab11
M55
Rab11
merge
Rab7
M55
Rab7
merge
Rab6
M55
Rab6
merge
GM130
M55
GM130
merge
Rab18
M55
Rab18
merge
CD44
M74
CD44
merge
AIFM1
M74
AIFM1
merge

Figure S8. **Subcellular distribution patterns of major membranous organelles in uninfected and 48 hrs MCMV-infected Balb 3T3 cells (related to Fig. 3).** Major organelles were visualized using Ab reagents to cellular proteins that characterize early endosomes (Rab5), endosomal recycling compartment (Rab11), late endosomes (Rab7), trans-Golgi network (Rab6), cis/medial-Golgi (GM130), endoplasmic reticulum (Rab18), cortical endomembrane system (CD44), and mitochondria (AIFM1). The sites of intracellular accumulation of viral envelope glycoproteins were visualized using mAb reagents to MCMV proteins M55 and M74. Antibody reagents used are listed in Table S1, and each marker described in Table S2. The primary Ab reagents were visualized using an appropriate combination of non-cross-reactive AF^{488}- and AF^{555}-conjugated secondary Ab reagents. Cell borders are indicated by fine dashed lines and nuclei by fine dotted lines. Bars, 10 µm.

Figure S9

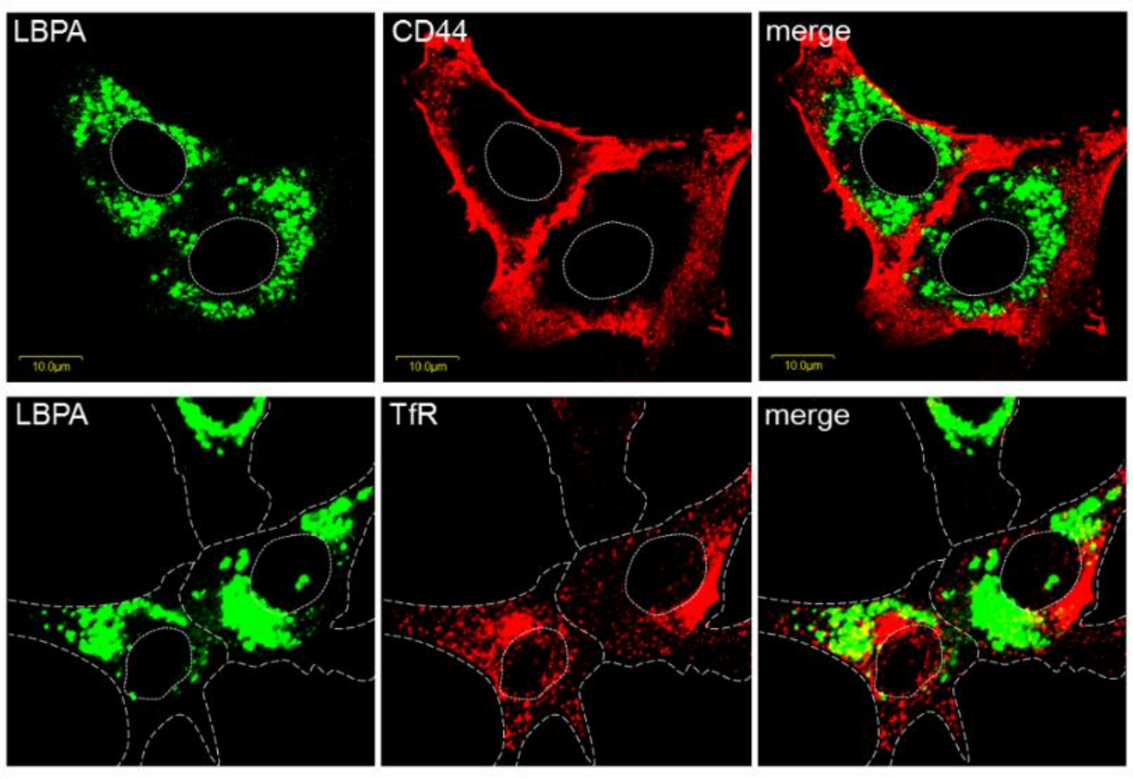

Figure S9. **Visualization of the main cytoplasmic zones confined by membranous organelles in uninfected Balb 3T3 cells** (related to Fig. 3). Plasma membrane and cortical zone membranous system can be confined by visualization of CD44, perinuclear zone membranous system by visualization of LEs using LBPA as a marker of luminal LE membranes (or CD63 and Lamp1, not shown), and juxtanuclear zone membranous system by visualization of the ERC using TfR as a marker. LBPA is absent from the juxtanuclear zone, whereas Lamp1 and CD63 can be found in the juxtanuclear zone. The primary Ab reagents (listed in Table S1) were visualized using an appropriate combination of non-cross-reactive AF^{488}- and AF^{555}-conjugated secondary Ab reagents. Cell borders are indicated by fine dashed lines and nuclei by fine dotted lines. Bars, 10 µm.

Figure S10

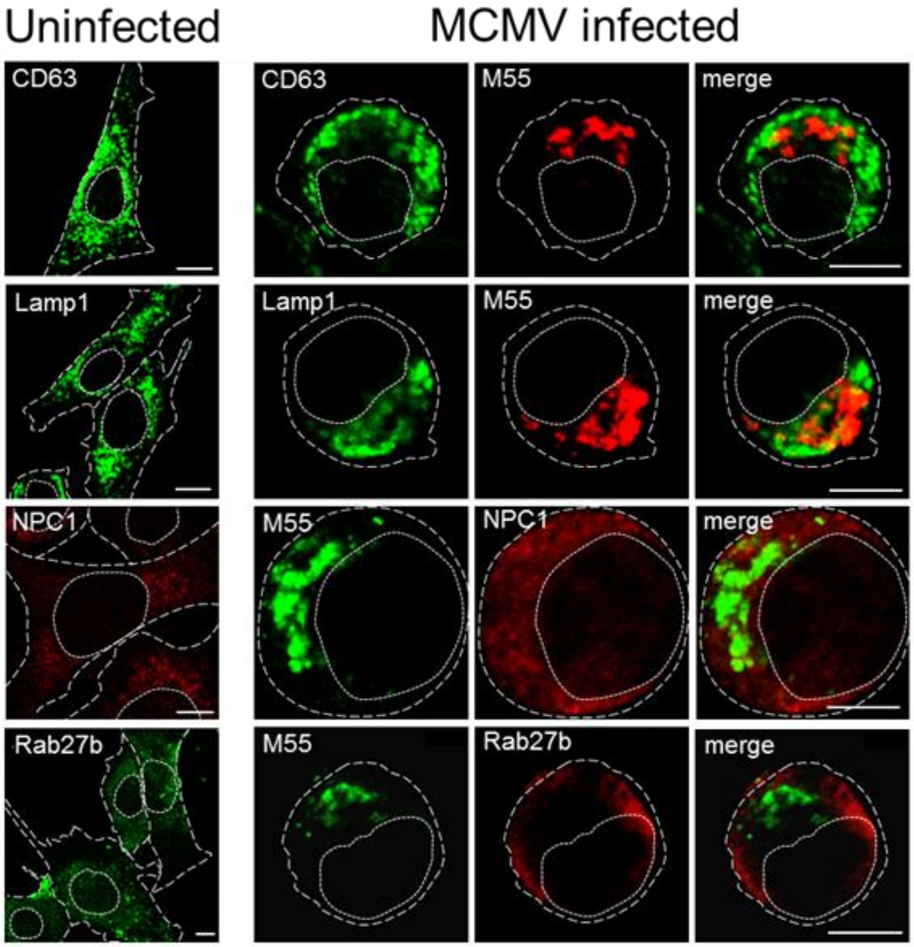

Figure S10. **The late endosomal (LE) system does not contribute to the AC** (related to Fig. 3)**.** The subcellular distribution of LEs membranes of uninfected and Δm138-MCMV infected (48 hpi) Balb 3T3 cells was visualized using Ab reagents to cellular proteins that distinguish subsets of LE membranes (Table S2). The sites of intracellular accumulation of viral envelope glycoproteins were visualized using mAb reagents to MCMV proteins M55. Antibody reagents used are listed in Table S1. The primary Ab reagents were visualized using an appropriate combination of non-cross-reactive AF^{488}- and AF^{555}-conjugated secondary Ab reagents. Cell borders are indicated by fine dashed lines and nuclei by fine dotted lines. Bars, 10 μm.

Figure S11

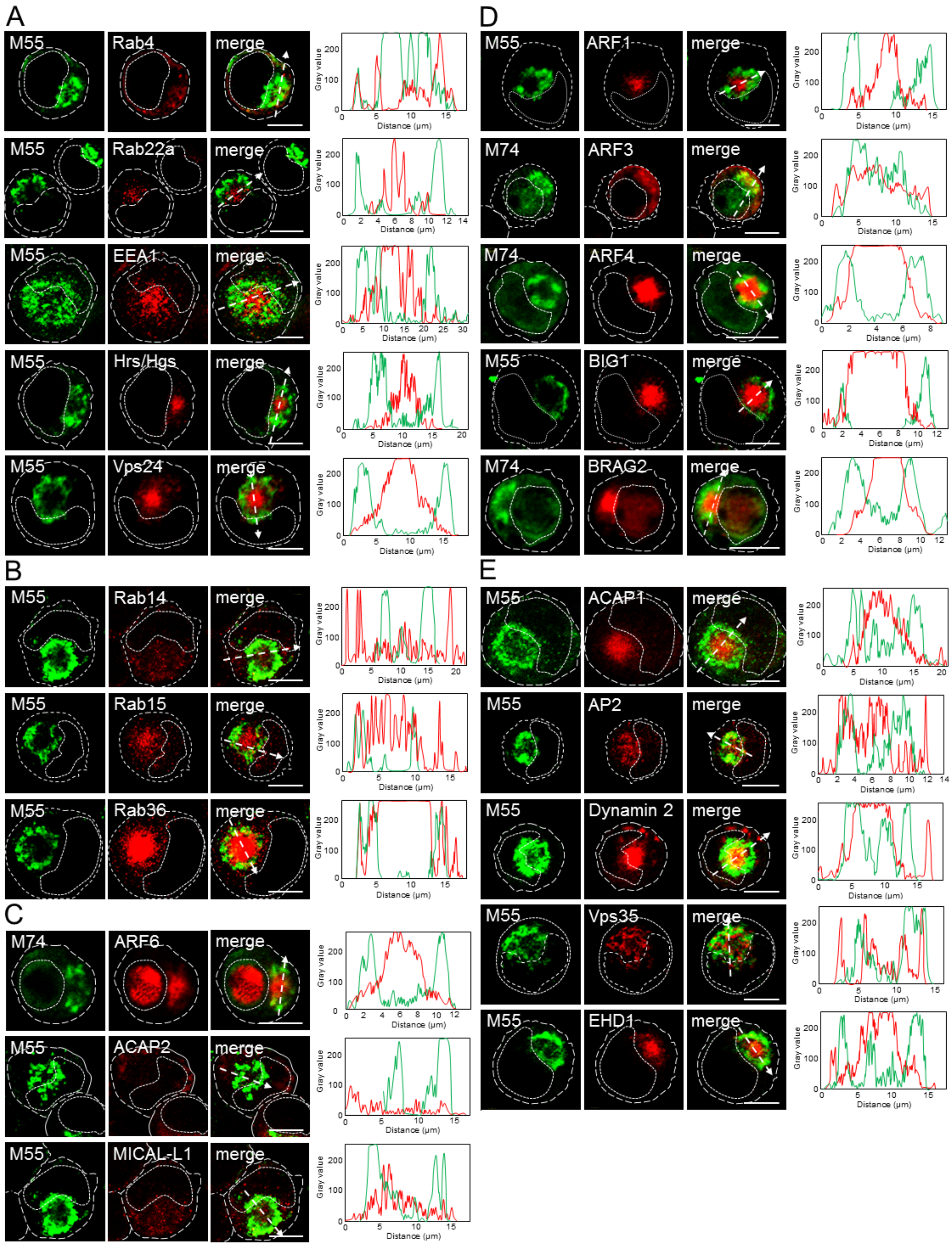
A
M55
Rab4
merge
Rab22a
EEA1
Hrs/Hgs
Vps24
B
Rab14
Rab15
Rab36
C
M74
ARF6
ACAP2
MICAL-L1
D
ARF1
ARF3
ARF4
BIG1
BRAG2
E
ACAP1
AP2
Dynamin 2
Vps35
EHD1
Gray value
Distance (μm)

Figure S11. **Subcellular distribution patterns of early endosomal (EE) and endosomal recycling compartment (ERC) markers and effector proteins at 48 hpi with MCMV** (related to Fig. 4)**.** Host-cell proteins (Table S2) and viral envelope glycoproteins (M55 and M74) of Δm138-MCMV infected (48 hpi) Balb 3T3 cells were stained using mAb reagents listed in Table S1. The primary Ab reagents were visualized using an appropriate combination of non-crossreactive AF^{488}- and AF^{555}-conjugated secondary Ab reagents. **(A)** EE proteins; **(B)** Small GTPases that recruit and may define different subsets of RE/ERC membranes; **(C)** Components of the ARF6-Rab35 axis that may act at the ERC; **(D)** Class I and class II ARFs and ARF-GEFs that may act at the RE/ERC; **(E)** Effector proteins that control scission at EE and ERC. Colocalization analysis was performed by plotting fluorescence intensity profiles along white dashed lines and shown in the right column. Cell borders are indicated by fine dashed lines and nuclei by fine dotted lines. Bars, 10 μm.

Figure S12

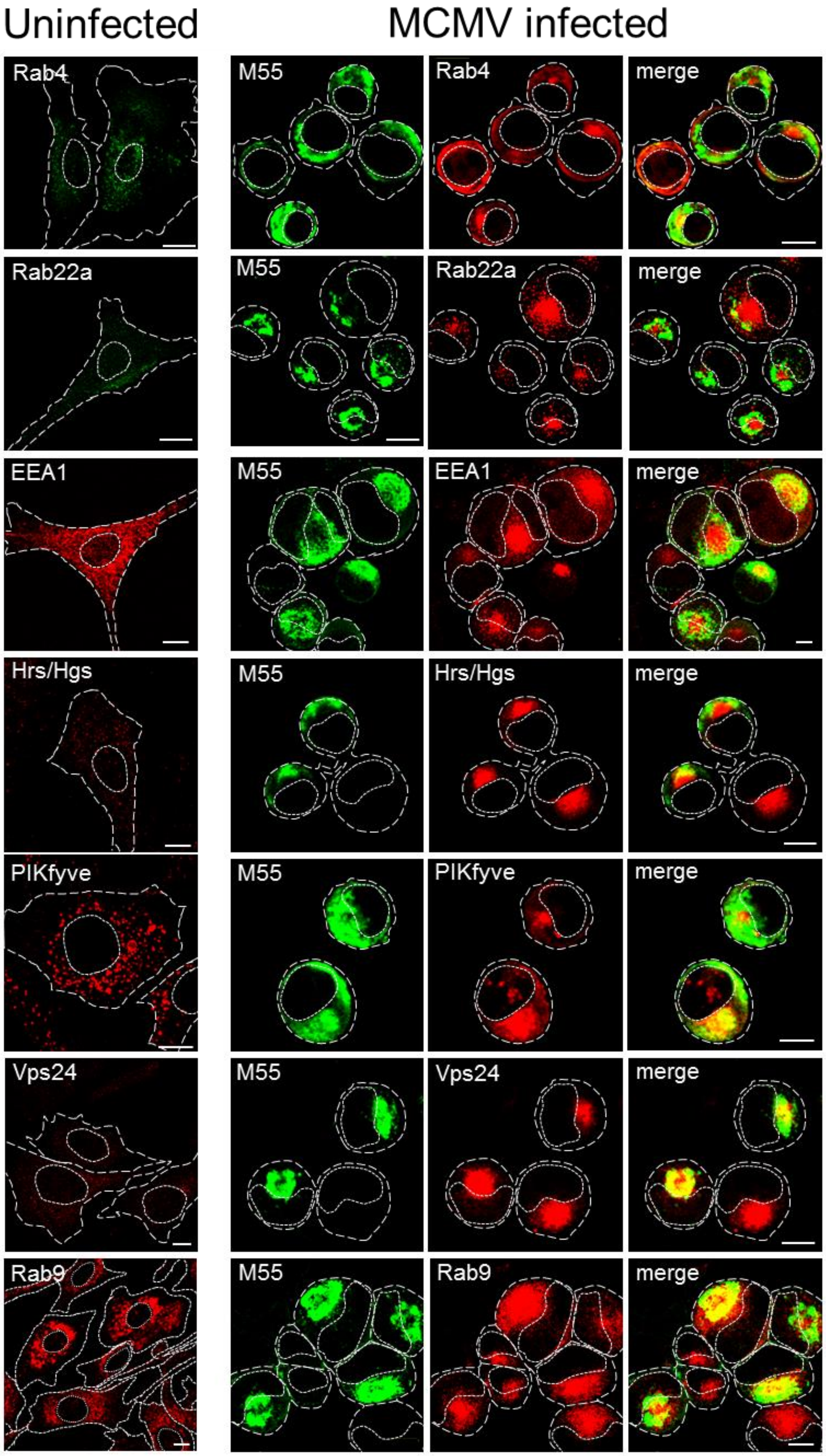
Uninfected
MCMV infected
Rab4
M55
Rab4
merge
Rab22a
M55
Rab22a
merge
EEA1
M55
EEA1
merge
Hrs/Hgs
M55
Hrs/Hgs
merge
PIKfyve
M55
PIKfyve
merge
Vps24
M55
Vps24
merge
Rab9
M55
Rab9
merge

Figure S12. **Contribution of the early endosomal (EE) system to the AC** (related to Fig. 4)**.** The subcellular distribution of EE membranes of uninfected and Δm138-MCMV infected (48 hpi) Balb 3T3 cells was visualized using Ab reagents to cellular proteins that distinguish subsets of EE membranes (Table S2). The sites of intracellular accumulation of viral envelope glycoproteins were visualized using mAb reagents to MCMV proteins M55. Antibody reagents used are listed in Table S1. The primary Ab reagents were visualized using an appropriate combination of non-cross-reactive AF^{488}- and AF^{555}-conjugated secondary Ab reagents. Cell borders are indicated by fine dashed lines and nuclei by fine dotted lines. Bars, 10 μm.

Figure S13

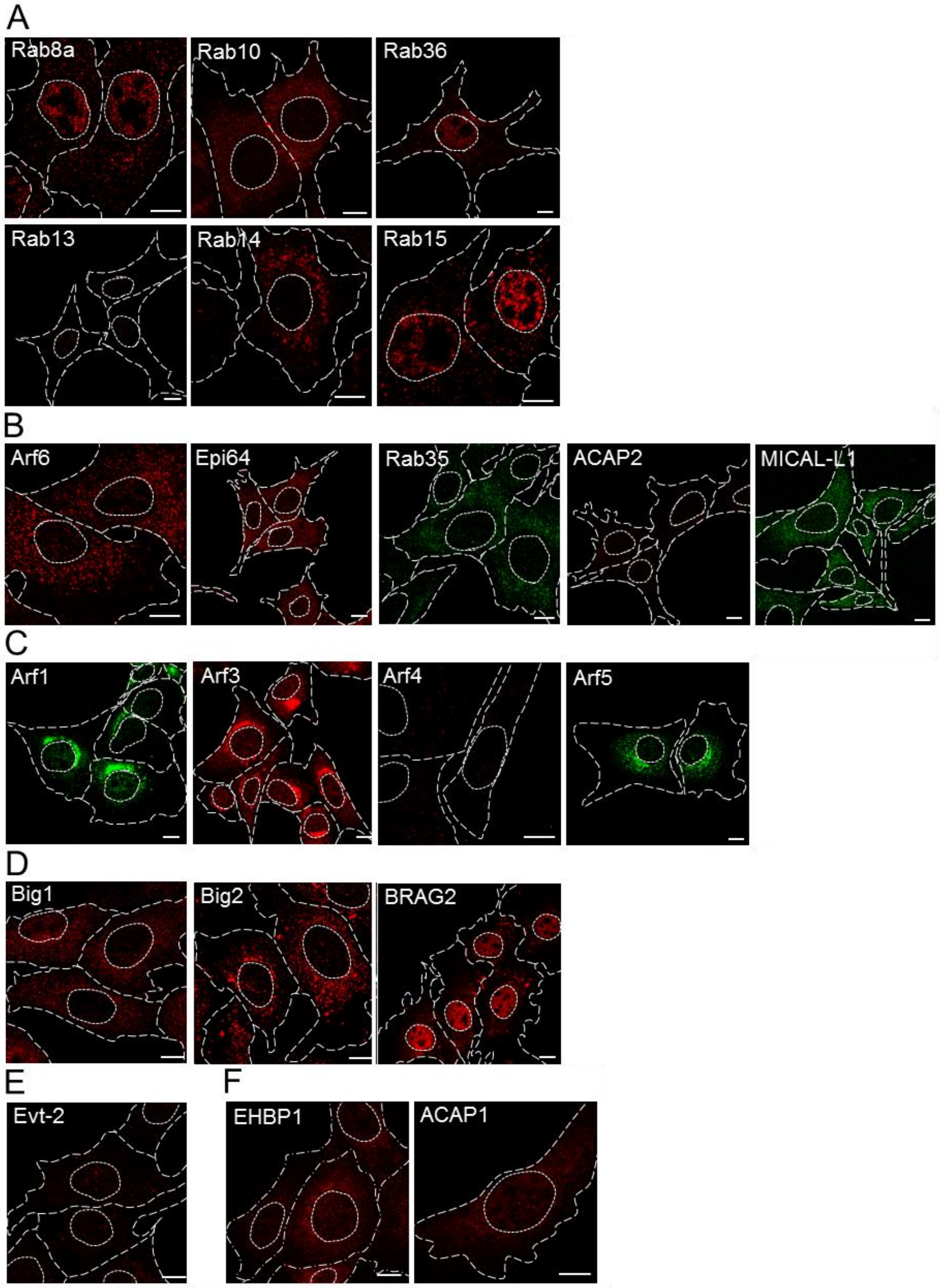

Figure S13. **Subcellular distribution of RE/ERC markers in uninfected Balb 3T3 cells** (related to Fig. 4). The subcellular distribution of RE/ERC markers (Table S2) was visualized using Ab reagents listed in Table S1. The primary Ab reagents were visualized using an appropriate combination of non-cross-reactive AF^{488}- or AF^{555}-conjugated secondary Ab reagents. Cell borders are indicated by fine dashed lines and nuclei by fine dotted lines. Bars, 10 µm.

Figure S14

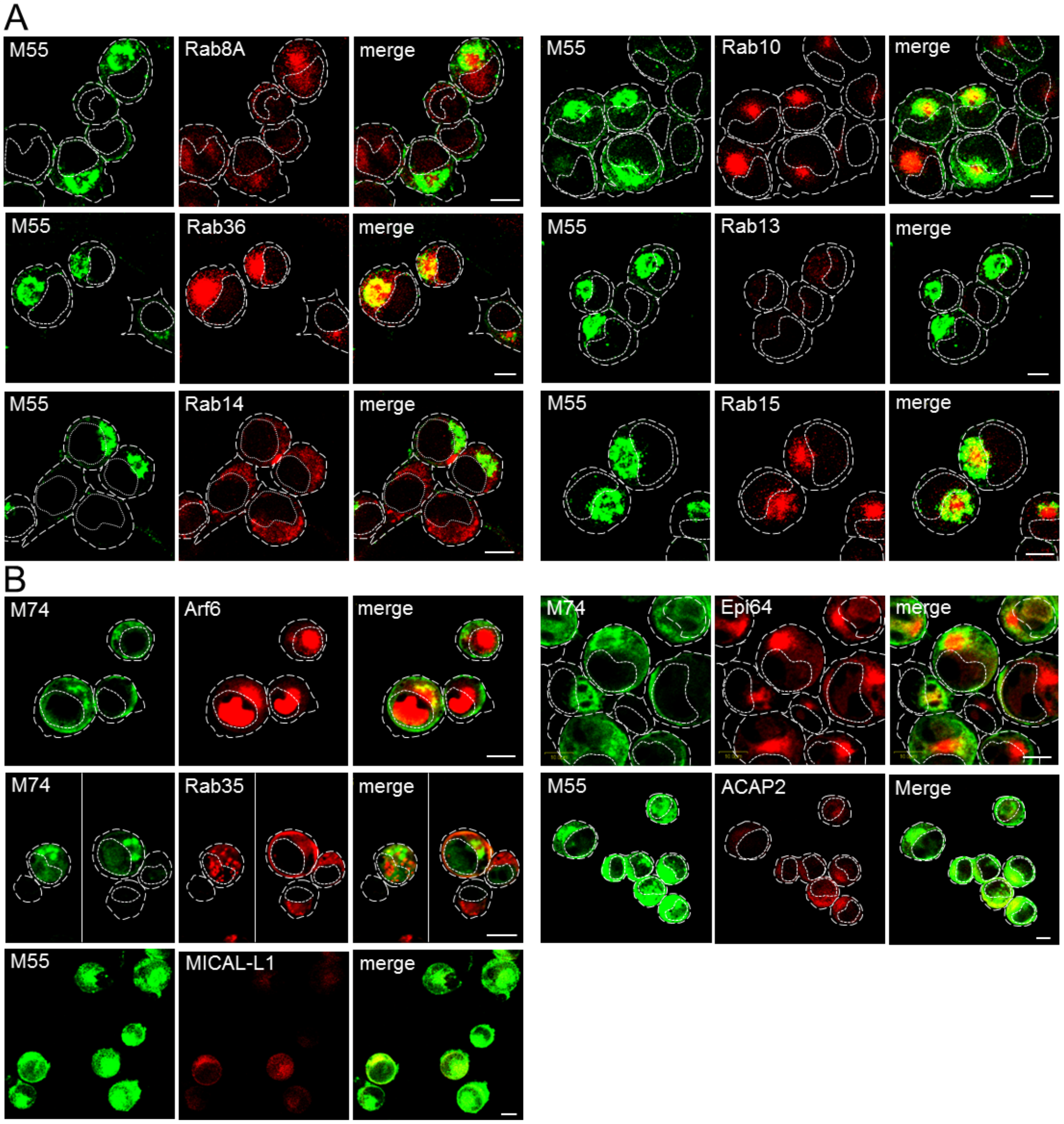
A
M55
Rab8A
merge
M55
Rab10
merge
M55
Rab36
merge
M55
Rab13
merge
M55
Rab14
merge
M55
Rab15
merge
B
M74
Arf6
merge
M74
Epi64
merge
M74
Rab35
merge
M55
ACAP2
Merge
M55
MICAL-L1
merge

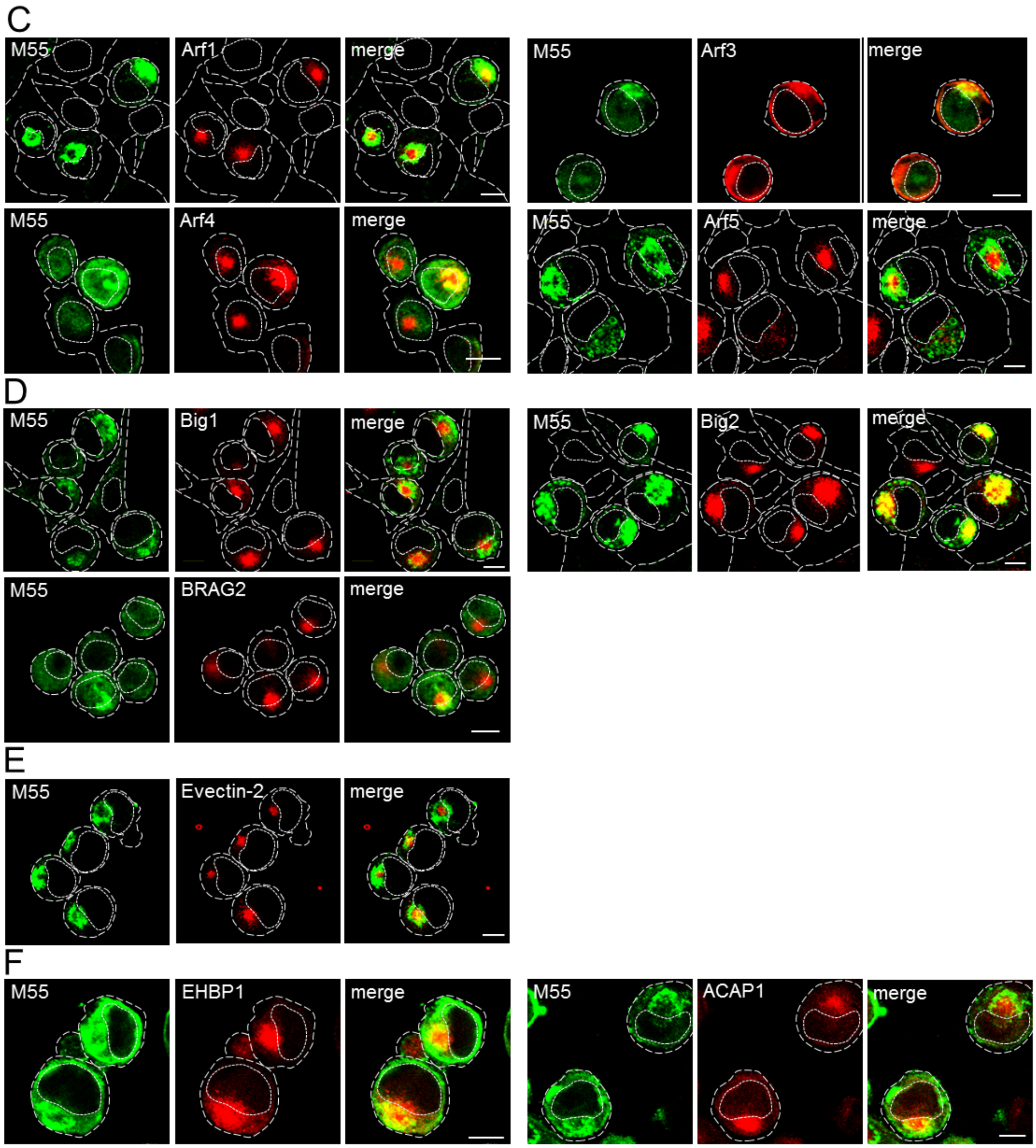

Figure S14. **Contribution of the recycling endosomes/endosomal recycling compartment (RE/ERC) to the AC (related to Fig 4).** The subcellular distribution of RE/ERC markers of Δm138-MCMV infected (48 hpi) Balb 3T3 cells was visualized using Ab reagents to cellular proteins (Table S2). The sites of intracellular accumulation of viral envelope glycoproteins were visualized using mAb reagents to MCMV proteins M55. Antibody reagents used are listed in Table S1. The primary Ab reagents were visualized using an appropriate combination of non-cross-reactive AF^{488}- and AF^{555}-conjugated secondary Ab reagents. (**A**) Small GTPases that recruit and may define different subsets of RE/ERC membranes. (**B**) ARF6-Rab35 axis that may act at the ERC. (**C**) Class I and class II ARFs. (**D**) ARF-GEFs that may act at the RE/ERC. (**E**) Evectin-2, phosphatidylserine-binding protein. (**F**) Rab10 effectors. Cell borders are indicated by fine dashed lines and nuclei by fine dotted lines. Bars, 10 μm.

Figure S15

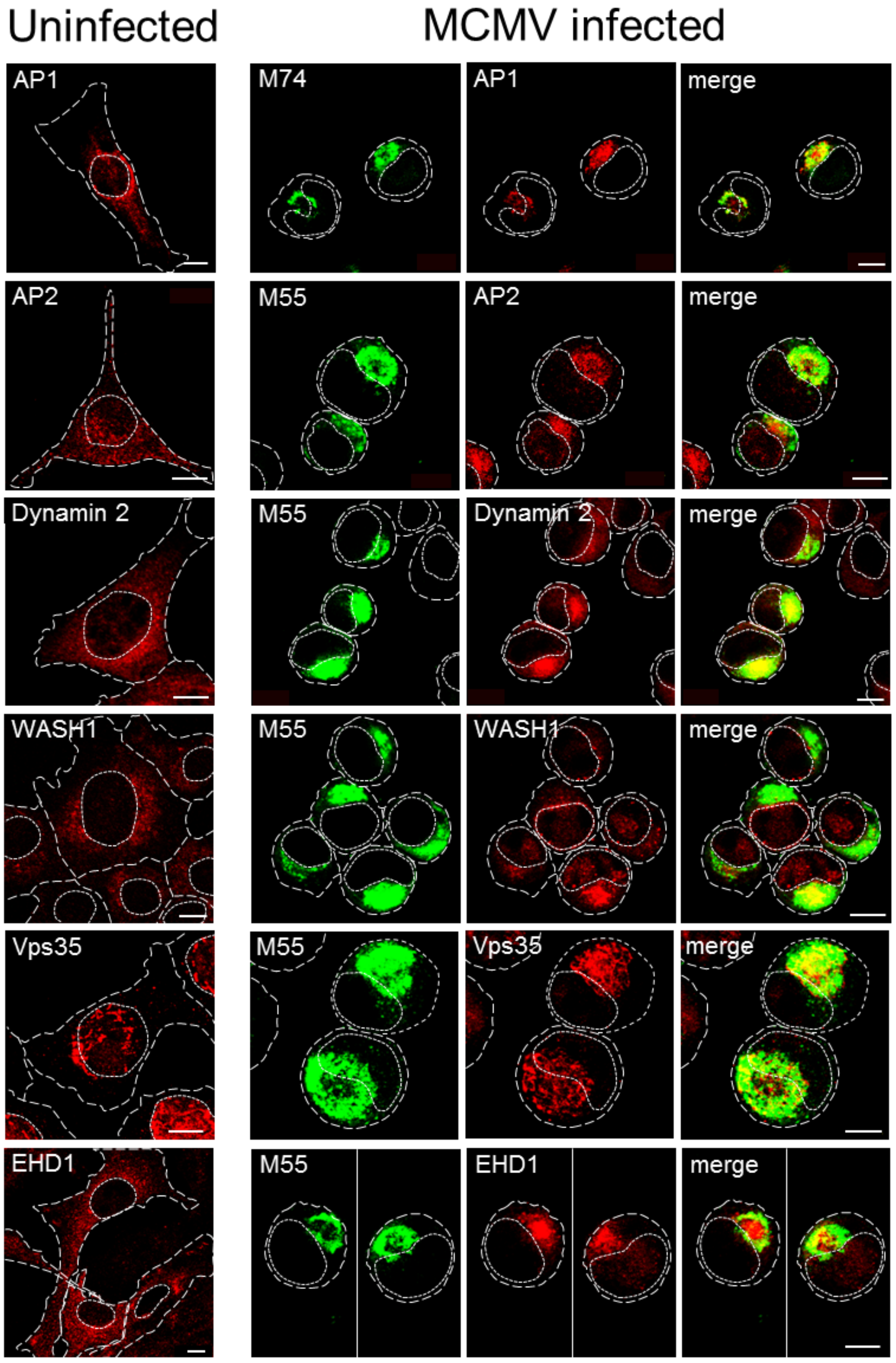

Figure S15. **Subcellular distribution of effector proteins that control scission at EE, ERC, and the TGN at 48 hpi with MCMV (related to Fig. 4).** Host-cell proteins (Table S2) and viral envelope glycoproteins (M55 and M74) were stained using mAb reagents listed in Table S1. The primary Ab reagents were visualized using an appropriate combination of non-cross-reactive AF^{488}- and AF^{555}-conjugated secondary Ab reagents. Colocalization analysis was performed by plotting fluorescence intensity profiles along white dashed lines and shown in the right column. Cell borders are indicated by fine dashed lines and nuclei by fine dotted lines. Bars, 10 µm.

Figure S16

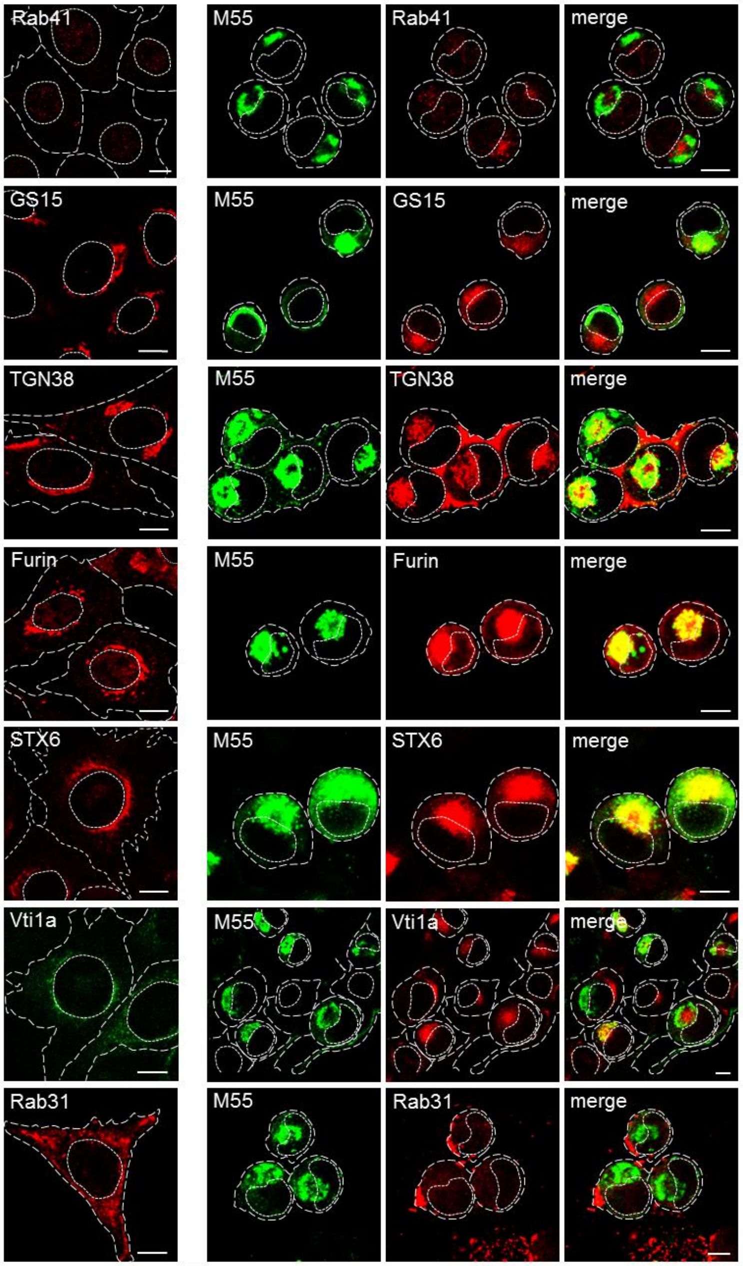

Figure S16. **Contribution of the Golgi and the trans-Golgi network (TGN) to the AC (**related to Fig. 5**).** The subcellular distribution of the Golgi and TGN markers in uninfected and Δm138-MCMV infected (48 hpi) Balb 3T3 cells was visualized using Ab reagents to cellular proteins (Table S2). Viral envelope glycoproteins were visualized using mAb reagents to MCMV proteins M55. Antibody reagents used are listed in Table S1. The primary Ab reagents were visualized using an appropriate combination of non-cross-reactive AF^{488}- and AF^{555}-conjugated secondary Ab reagents. Cell borders are indicated by fine dashed lines and nuclei by fine dotted lines. Bars, 10 μm.

Figure S17

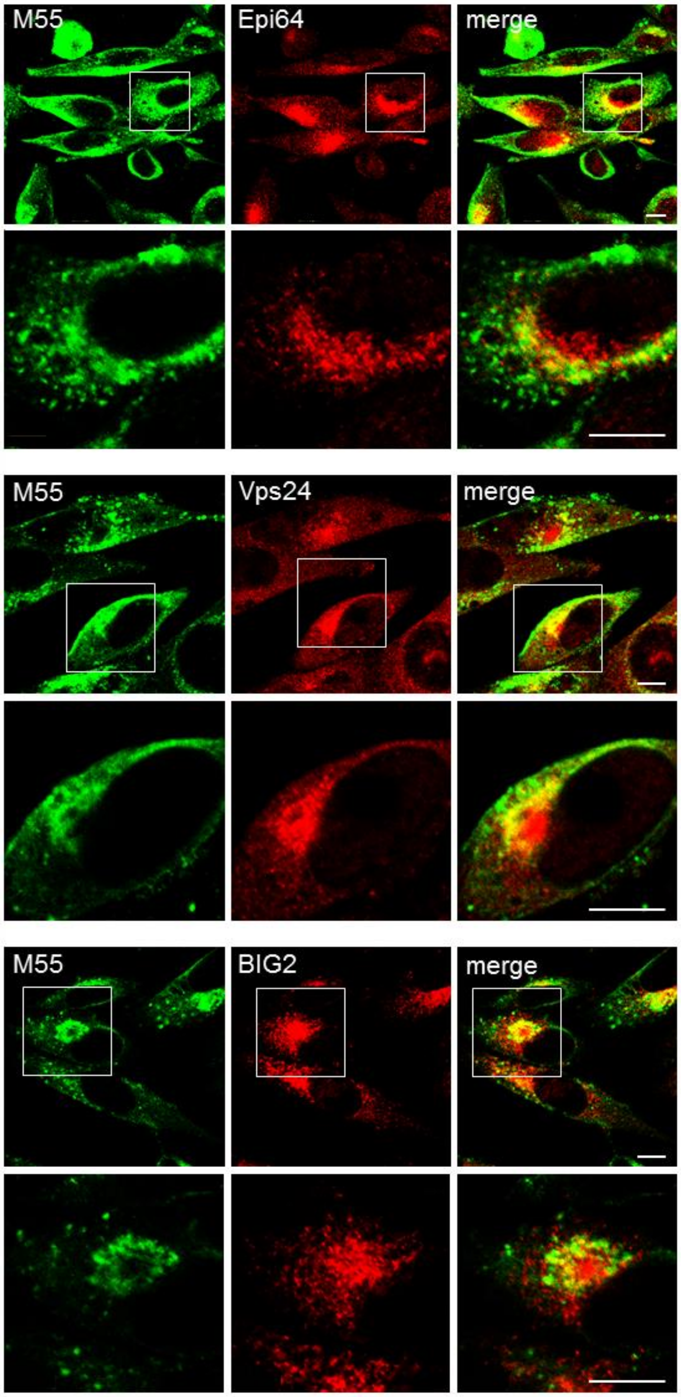

Figure S17. **Membranous organelle reorganization in cells infected with the Δ9-MCMV** (related to Fig. 8)**.** Balb 3T3 cells were infected with Δ9-MCMV (with deleted M23-M26 genes) and analyzed at 30 hpi for subcellular localization of selected EE and ERC markers (Epi64, Vps24, and BIG2) and M55 proteins. Lower panel images represent the boxed area acquired at higher magnification. Bars, 10 μm.

Figure S18

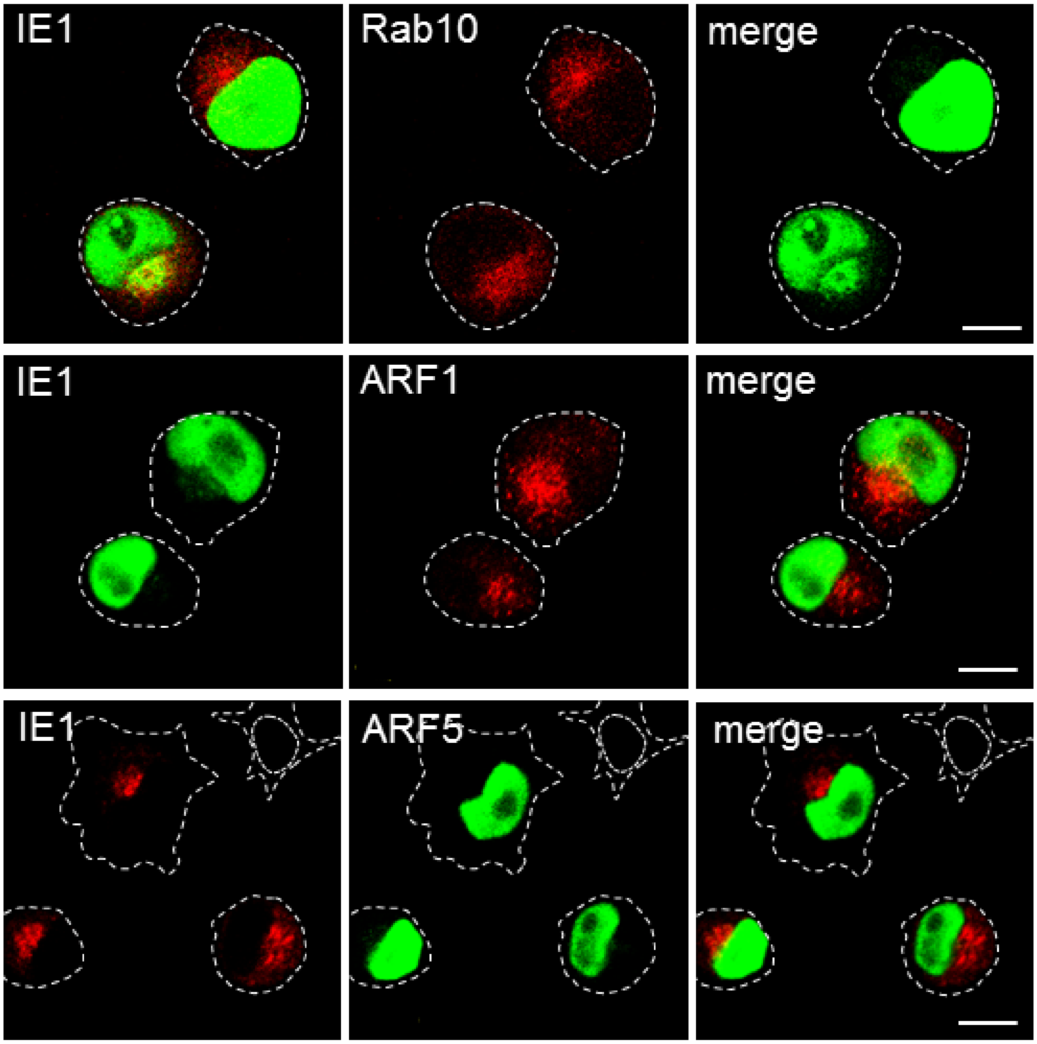

Figure S18. **Reorganization of membranous organelle system in DC2.4 cells (related to Fig. 9).** The subcellular distribution of Rab10, ARF1, and ARF5 were determined by immunofluorescence on DC2.4 cells infected with Δm138-MCMV infected at 18 hpi. Antibody reagents used are listed in Table S1. The primary Ab reagents were visualized using an appropriate combination of non-cross-reactive AF^{488}- and AF^{555}-conjugated secondary Ab reagents. Cell borders are indicated by fine dashed lines and nuclei by fine dotted lines. Bars, 10 µm.
