# Supplementary material for: Cytomegalovirus Generates Assembly Compartment in the Early Phase of Infection by Perturbation of Host-Cell Factors Recruitment at the Early Endosome/Endosomal Recycling Compartment/Trans-Golgi Interface
Source: Front Cell Dev Biol. 2020 Sep 11;8:563607. doi: 10.3389/fcell.2020.563607 (PMC7516400; doi:10.3389/fcell.2020.563607)
Supplement: Supplementary file 7 [file Data_Sheet_7.PDF]

Supplementary figure S19
Expression of marker genes in MCMV infected cells

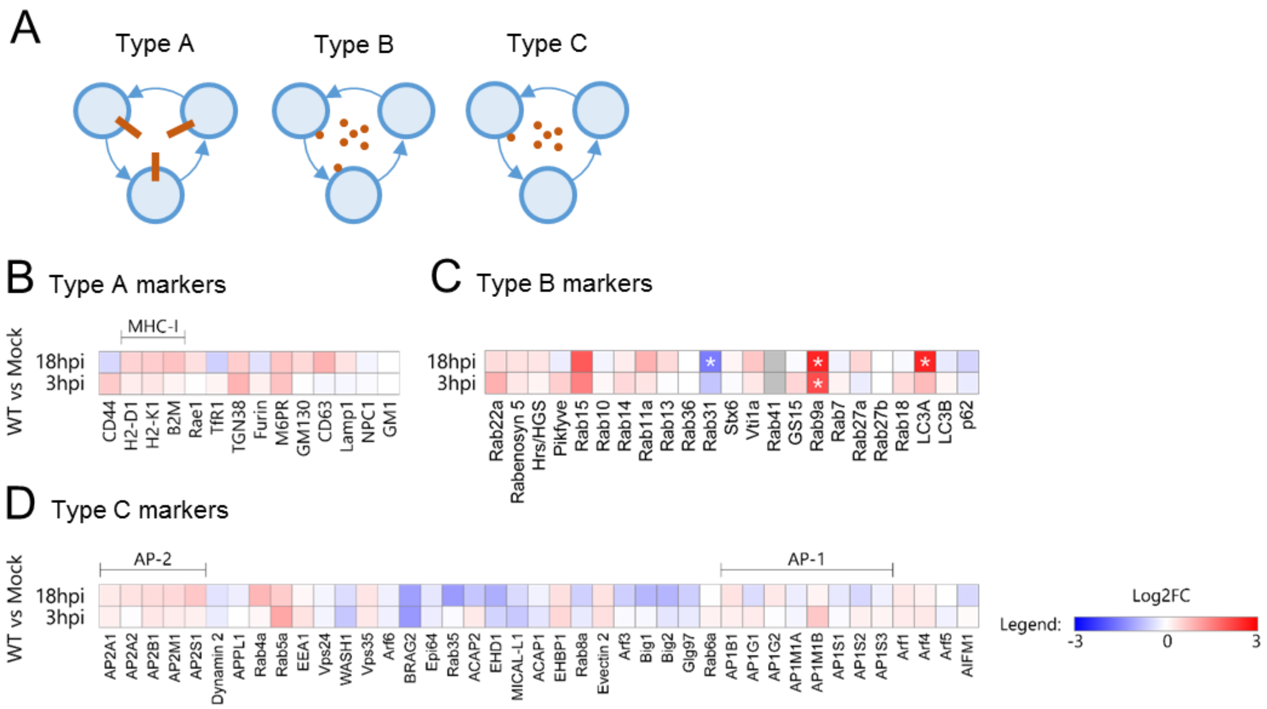

Figure S19. **Effect of MCMV infection on transcriptional activity of genes that encode host-cell proteins used as markers in immunofluorescence studies (**related to Fig. 9**).** (**A**) Schematic presentation of the intracellular itinerary of classified markers. (**B-D**) Transcriptional activity (transcriptome analysis) of genes that encode type A, B, and C markers at the beginning (3 hpi) and the end (18 hpi) of the early phase of MCMV infection. The data represent the fold change (log2) of gene expression at the beginning (3 hpi) and the end (18 hpi) of the early phase of MCMV infection relative to the mock-infected cells. Statistically significant genes are designated with an asterisk (*).
