# Supplementary material for: Cytomegalovirus Generates Assembly Compartment in the Early Phase of Infection by Perturbation of Host-Cell Factors Recruitment at the Early Endosome/Endosomal Recycling Compartment/Trans-Golgi Interface
Source: Front Cell Dev Biol. 2020 Sep 11;8:563607. doi: 10.3389/fcell.2020.563607 (PMC7516400; doi:10.3389/fcell.2020.563607)
Supplement: Supplementary file 8 [file Data_Sheet_8.DOCX]

Study primary accesion number: PRJEB39860

Study secondary accesion number: ERP123425

Samples accesion numbers:

| **Primary Accession** | **Secondary Accession** | **Title** | **Tax ID** | **Scientific Name** | **Common Name** | **Submission Date** | **Status** |
| --- | --- | --- | --- | --- | --- | --- | --- |
| ERS4945339 | SAMEA7185098 | DC2.4_mock_3h_replicate_1 | 10090 | Mus musculus | house mouse | 13-Aug-2020 | Public |
| ERS4945340 | SAMEA7185099 | DC2.4_mock_3h_replicate_2 | 10090 | Mus musculus | house mouse | 13-Aug-2020 | Public |
| ERS4945341 | SAMEA7185100 | DC2.4_mock_3h_replicate_3 | 10090 | Mus musculus | house mouse | 13-Aug-2020 | Public |
| ERS4945342 | SAMEA7185101 | DC2.4_wtMCMV_3h_replicate_1 | 10090 | Mus musculus | house mouse | 13-Aug-2020 | Public |
| ERS4945343 | SAMEA7185102 | DC2.4_wtMCMV_3h_replicate_2 | 10090 | Mus musculus | house mouse | 13-Aug-2020 | Public |
| ERS4945344 | SAMEA7185103 | DC2.4_wtMCMV_3h_replicate_3 | 10090 | Mus musculus | house mouse | 13-Aug-2020 | Public |
| ERS4945345 | SAMEA7185104 | DC2.4_wtMCMV_18h_replicate_1 | 10090 | Mus musculus | house mouse | 13-Aug-2020 | Public |
| ERS4945346 | SAMEA7185105 | DC2.4_wtMCMV_18h_replicate_2 | 10090 | Mus musculus | house mouse | 13-Aug-2020 | Public |
| ERS4945347 | SAMEA7185106 | DC2.4_wtMCMV_18h_replicate_3 | 10090 | Mus musculus | house mouse | 13-Aug-2020 | Public |
